# Natural Selection Equally Supports the Human Tendencies in Subordination and Domination: A Genome-Wide Study With *in silico* Confirmation and *in vivo* Validation in Mice

**DOI:** 10.3389/fgene.2019.00073

**Published:** 2019-02-20

**Authors:** Irina Chadaeva, Petr Ponomarenko, Dmitry Rasskazov, Ekaterina Sharypova, Elena Kashina, Maxim Kleshchev, Mikhail Ponomarenko, Vladimir Naumenko, Ludmila Savinkova, Nikolay Kolchanov, Ludmila Osadchuk, Alexandr Osadchuk

**Affiliations:** ^1^Institute of Cytology and Genetics, Siberian Branch of Russian Academy of Sciences, Novosibirsk, Russia; ^2^Novosibirsk State University, Novosibirsk, Russia; ^3^University of La Verne, La Verne, CA, United States

**Keywords:** gene, promoter, TBP, TATA-box, SNP, expression change, social hierarchy, candidate SNP marker

## Abstract

We proposed the following heuristic decision-making rule: “IF {an excess of a protein relating to the nervous system is an experimentally known physiological marker of low pain sensitivity, fast postinjury recovery, or aggressive, risk/novelty-seeking, anesthetic-like, or similar agonistic-intolerant behavior} AND IF {a single nucleotide polymorphism (SNP) causes overexpression of the gene encoding this protein} THEN {this SNP can be a SNP marker of the tendency in dominance} WHILE {underexpression corresponds to subordination} AND *vice versa*.” Using this decision-making rule, we analyzed 231 human genes of neuropeptidergic, non-neuropeptidergic, and neurotrophinergic systems that encode neurotrophic and growth factors, interleukins, neurotransmitters, receptors, transporters, and enzymes. These proteins are known as key factors of human social behavior. We analyzed all the 5,052 SNPs within the 70 bp promoter region upstream of the position where the protein-coding transcript starts, which were retrieved from databases Ensembl and dbSNP using our previously created public Web service SNP_TATA_Comparator (http://beehive.bionet.nsc.ru/cgi-bin/mgs/tatascan/start.pl). This definition of the promoter region includes all TATA-binding protein (TBP)-binding sites. A total of 556 and 552 candidate SNP markers contributing to the dominance and the subordination, respectively, were uncovered. On this basis, we determined that 231 human genes under study are subject to natural selection against underexpression (significance *p* < 0.0005), which equally supports the human tendencies in domination and subordination such as the norm of a reaction (plasticity) of the human social hierarchy. These findings explain vertical transmission of domination and subordination traits previously observed in rodent models. Thus, the results of this study equally support both sides of the century-old unsettled scientific debate on whether both aggressiveness and the social hierarchy among humans are inherited (as suggested by Freud and Lorenz) or are due to non-genetic social education, when the children are influenced by older individuals across generations (as proposed by Berkowitz and Fromm).

## Introduction

Social dominance-subordination hierarchy is a set of structured relationships between individuals. These relationship ensure coexistence of individuals by reducing mutual aggression and increasing order in the competition for limited environmental resources as well as elevating their reproductive potential (Hinde, 1970; Rowell, 1974). In animals, such intraspecies hierarchy is a result of agonistic aggressive behavior defined by ethologists as an innate form of action to protect oneself, shelter, progeny, and territory (Lorenz, 2002). Artificial selection of animals for either aggressiveness (Kulikov et al., 2016) or domestication (Belyaev, 1979) has demonstrated the contribution of genetic factors to the phenotypic manifestation of aggressiveness (Ehrman and Parsons, 1981; Moore, 2013). Finally, a genome-wide search for genetic factors of both fear and aggressive behaviors has been conducted on model animals, e.g., in canines, which were artificially selected for both domestication and agonistic behavior (Zapata et al., 2016).

In humans, the reference genome (Colonna et al., 2014) and the full set of single-nucleotide polymorphisms (SNPs) available in the public databases Ensembl (Zerbino et al., 2015) and dbSNP (Sherry et al., 2001). In humans, genetic polymorphism exemplifies the results of natural selection rather than artificial one Dobzhansky (1963) concluded: “man is genetically specialized to be unspecialized,” meaning that human behavioral tolerance to social and environmental challenges is broad. The recent genome-wide comparison between humans and apes (Gunbin et al., 2018) indicated that the origin of human species coincided with a reliable increase in the plasticity of the transcription regulation of neuronal genes, while in apes the regulatory plasticity of these genes reduced. This observation points at the action of destabilizing (disruptive) natural selection rather than directional or stabilizing natural selection (Belyaev, 1979). Notably, comprehensive multifactorial regression analysis of healthy young athletes (i.e., boxers, kick boxers, and karate fighters) revealed a significant positive correlation between their aggression and anxiety rates, which helps to achieve top combat levels owing to the prevention of injuries under extreme conditions in the arena (Tiric-Campara et al., 2012). Finally, there is the century-old unsettled scientific dispute where one side – e.g., Freud (1921, 1930) and Lorenz (1964, 2002) – explains both human aggressiveness and social hierarchy as a consequence of their genetic predisposition, while the other side – e.g., Fromm (1941, 1973), Berkowitz (1962, 1993), and Skinner (Rogers and Skinner, 1956; Skinner, 1981) – explains this by the continuous non-genetic social education which continues from childhood to the oldest age (Markel, 2016).

Notably, the social dominance-subordination hierarchy in social species (e.g., humans) limits the permissible aggression range, which is under pressure of natural selection as a norm of a reaction (plasticity) to aggressive behavior (Eldakar and Gallup, 2011). Conditions, quality, and the lifespan of an individual depend on his∖her rank within the social hierarchy (Michopoulos et al., 2012). In murine micropopulations as combinations of inbred and hybrid individuals, manifestation of the social dominance phenotype reliably depends on some behavioral features taken together with a genotype (Serova et al., 1991). As for human aggressiveness as a target of some antipsychotic drugs [e.g., olanzapine (Ellingrod et al., 2005)], there are a number of biomedical SNP markers that represent statistically significant differences between the reference human genome and the individual genome of patients having either a certain psychiatric disease or resistance/susceptibility to certain treatments of this disease.

Each discovery of the SNP markers associated with the human phenotypic traits had been a unique success in the pregenomic era, whereas now, this task is one of the major aims of the largest scientific project: “1000 genomes” (Colonna et al., 2014). The main results of this project are publicly available within two regularly synchronized and updated databases Ensembl (Zerbino et al., 2015), which is the reference human genome consisting of the most frequent (ancestral) nucleotides at each DNA position, and dbSNP (Sherry et al., 2001) as the human variome containing all the carefully verified SNPs. Now these databases contain a carefully curated extract that summarizes information on more than 10000 individual human genomes and more than 100 million SNPs (Telenti et al., 2016). As for all the 8.58 billion possible human whole-genome SNPs, creation of a relevant database, dbWGFP, was already reported (Wu et al., 2016); this database is designed to compile all the available information about each of these SNPs to use it in the nearest future to handle the requests from the people who want to sequence their own individual genome and, then, get his/her individual benefits from it.

Because biomedical SNP markers may be used for diagnosis and selection of treatments for humans, there is only one acceptable approach to identify them: that is, to estimate the statistical significance of differences in the prevalence of a given SNP in the representative cohorts of individuals with the phenotypic trait of interest (Varzari et al., 2018). It is unlikely that this extremely time-consuming and expensive procedure is applicable to each of the 8.58 billion possible human SNPs (Abbas et al., 2006). Moreover, both Haldane’s dilemma (Haldane, 1957) and Kimura’s theory of neutral evolution (Kimura, 1968) predict neutrality of the absolute majority of human SNPs. These neutral SNPs should be discarded by computer-based calculations in order to reduce the total cost of biomedical SNP markers. Currently, there are many public Web services (e.g., Bendl et al., 2016), predicting candidate SNP markers and eliminating the most probable neutral SNPs while taking into account various similarity measures for genome-wide data during infections (Leschner et al., 2012) or diseases (Hu et al., 2013) as well as after treatment (Hein and Graver, 2013) and in health (Ni et al., 2012). The accuracy of these similarity-based predictions increases with the increase in diversity of available genome-wide data, in agreement with our predictions (Ponomarenko et al., 1999) based on Central Limit Theorem.

The best accuracy of these bioinformatics predictions corresponds to SNPs in the protein-coding regions owing to their reliable manifestation as protein damage, whereas in the case of SNPs in the regulatory regions of genes, none of the proteins is damaged (Amberger et al., 2015). Notably, the 70 bp promoter regions in front of the transcription start sites (TSSs) contain the majority of the clinically verified regulatory SNP markers (Ponomarenko et al., 2013) due to the TATA-binding protein (TBP)-binding site (e.g., TATA-box), which is obligatory for the primary initiation of gene transcription (Martianov et al., 2002). Finally, Mogno et al. (2010) experimentally found that the increase in TBP-binding affinity for the TBP-binding sites altered by SNPs causes overexpression of the appropriate genes whereas underexpression corresponds to a decrease in the affinity.

In our previous works, we created a public Web service SNP_TATA_Comparator (see text footnote 1) (Ponomarenko et al., 2015) for selecting the statistically significant SNP-caused alterations in TBP’s affinity for the promoter regions 70 bp upstream of the protein-coding TSSs. This Web service is based on our three-step model of the TBP–promoter binding to each other (Ponomarenko et al., 2008), namely: (i) TBP slides along DNA ↔ (ii) TBP stops at a putative TBP-binding site ↔ (iii) the TBP–promoter complex is fixed by the DNA bending at a right angle, as was experimentally discovered (Delgadillo et al., 2009). Using SNP_TATA_Comparator, we predicted candidate SNP markers – within TBP-binding sites of the human gene promoters – associated with obesity, chronopathology, aggressiveness, and autoimmune and Alzheimer’s diseases (for review, see Ponomarenko P. et al., 2017). Recently, we preliminarily studied (Chadaeva et al., 2017) the possibility to predict candidate SNP markers for social hierarchy using a short representative set of 21 human genes homologous to the animal genes encoding the known physiological markers of aggressiveness, which represent nervous, endocrine, immune, respiratory, vascular, muscular, and other systems of the human body.

In this work, due to our observation (Bragin et al., 2006) of domination of adult male BALB/cLac mice over CBA/Lac mice, we made a genome-wide prediction for the human tendencies dominance and subordination within the framework of the neuropeptidergic, non-neuropeptidergic, and neurotrophinergic systems and verified it using a mouse model of human inheritance. We discuss how our results fit both genetic (e.g., Freud and Lorenz) and non-genetic (e.g., Berkowitz and Fromm) irreconcilable sides of the century-old scientific debate about the origin of both aggressiveness and social hierarchy in humans.

## Materials and Methods

### Animals

This study was carried out in accordance with the recommendations of Directive 2010/63/EU of the European Parliament and of the Council of September 22, 2010, on the protection of animals used for scientific purposes. Manipulations of animals and experimental procedures were performed in compliance with the international rules according to the “Guidelines for the care and use of mammals in neuroscience and behavioral research”^1^. The research protocol was approved by the Interinstitutional Commission on Bioethics at the ICG SB RAS, 10 Lavrentyev Avenue, Novosibirsk, Russia.

Analysis of the inheritance of agonistic behavior indicators and social dominance levels was conducted on 230 adult male mice that are diallelic crosses of a set of five maternal inbred mouse strains (i.e., PT, DD, YT, A/He, and C57BL/6J) with two analytic inbred paternal strains (BALB/cLac and CBA/Lac) of the murine tendencies in dominance and subordination, respectively, as determined experimentally previously (Bragin et al., 2006).

All the mice were maintained under standard conditions of a conventional animal facility of the ICG SB RAS.

### Identification of Inheritance of the Mouse Tendencies in Dominance and Subordination

One can see all the 230 diallelic crosses in Table 1, where five rows and two columns present F1 males. In each row of this table, there are descendants of mothers of the same inbred strain. Thus, the maternal non-genetic (pre- and postnatal) and cytoplasmic effects are the same for males of the same row of this table. To exclude non-genetic paternal postnatal effects on offspring, pregnant female mice were isolated from male mice.

**Table 1:** The experimental design for identification of inheritance of the murine tendencies in dominance and subordination.

Paternal genotype Maternal genotype	BALB/cLac	CBA/Lac
PT	PT × BALB/cLac (31)	PT × CBA/Lac (31)
C57Bl/6J	C57Bl/6J × BALB/cLac (20)	C57Bl/6J × CBA/Lac (20)
YT	YT × BALB/cLac (21)	YT × CBA/Lac (21)
DD	DD × BALB/cLac (20)	DD × CBA/Lac (20)
A/He	A/He × BALB/cLac (23)	A/He × CBA/Lac (23)

*The number of male mice for each of the 10 F1 hybrids is indicated in parentheses.*

We made up groups of F1 hybrid male mice with the minimal society size, namely: two males each: one from each column of the same row of Table 1. In each pair, both male mice had identical age, weight, and body size, but visually differed from each other in color. This approach allowed us to estimate the influence of the paternal genotype on the social dominance level of the appropriate F1 crosses.

A total of 115 experimental pairs (230 F1 hybrids) were distributed into five groups, corresponding to the maternal inbred strains (see Table 1). For each mouse male pair tested, we performed 14 observations (20 min each) during 5 days. Each observation was recorded using a video camera in automatic mode with a fixed period. Next, we analyzed these video recordings using the protocols of software The Observer XT 7.0 (version: 7.0, Noldus Information Technology, license No. OB070-03670). This way, we identified the social rank for each male within the appropriate pair according to asymmetry in agonistic behavior, in particular, by means of attacks and submissive poses as described in the Supplementary Experiment (Supplementary File S6).

### The Basic Decision-Making Rule

Both domesticated and laboratory animals are artificially selected using the known target traits (Belyaev, 1979; Kulikov et al., 2016), which can help in any computer-based genome-wide analysis of these animals (e.g., Zapata et al., 2016) in contrast to the human genome, which is the result of natural selection in favor of unknown unspecializing target traits (Dobzhansky, 1963). Hence, on the basis of our preliminary work (Chadaeva et al., 2017), we proposed the following heuristic decision-making rule: “IF {an excess of a protein relating to the nervous system is an experimentally known physiological marker of low pain sensitivity, fast post-injury recovery, or aggressive, fearless, impulsive, anxious, exploratory, risk/novelty-seeking, anesthetic-like, or similar agonistic-intolerant behavior} AND IF {a given SNP can cause overexpression of a gene encoding this protein} THEN {this SNP can be a SNP marker of predisposition to social dominance} WHILE {the underexpression corresponds to subordination} AND *vice versa*.” This whole study is devoted to evaluation of this decision-making rule.

### DNA Sequences

Using the aforementioned basic decision-making rule (see subsection “The Basic Decision-Making Rule”), we analyzed all the 5052 SNPs retrieved from the dbSNP database (build 150, Sherry et al., 2001), which are found within the 70 bp promoter regions upstream of the protein-coding transcripts of all the 231 human genes of the neuropeptidergic, non-neuropeptidergic, and neurotrophinergic systems retrieved from database Ensembl (GRCh38/hg38 assembly, Zerbino et al., 2015), which are listed in the alphabetic order in the first columns of Supplementary Tables S1–S3, respectively (hereinafter: see Supplementary Files S1–S3, respectively). These genes encode proteins that are known as key factors altering human social behavior, namely, neurotrophic and growth factors, interleukins, neurotransmitters, receptors, transporters, and enzymes.

Using our public Web service SNP_TATA_Comparator (Ponomarenko et al., 2015), we compared the DNA sequences of the ancestral (wt) and minor (min) alleles of SNPs of the 70 bp promoter region of these genes. We applied it together with the public Web service UCSC Genome Browser (Haeussler et al., 2015) and two public databases dbSNP (Sherry et al., 2001) and ClinVar (Landrum et al., 2014), as described in the Supplementary Web-service (Supplementary File S5). As a result, we obtained two pairs of (–ln(K_D_^(wt)^) ± δ_(wt)_) and (–ln(K_D_^(min)^) ± δ_(min)_) values of TBP affinity for these alleles of the promoter being studied according to contextual, conformational, and physicochemical changes in its B-helical DNA under the influence of a given SNP, as described in the Supplementary Method (Supplementary File S4). Next, we calculated Fisher’s Z-score as follows: Z = abs[ln(K_D_^(min)^/K_D_^(wt)^)]/[δ^2^_(min)_+δ^2^_(wt)_]^1/2^, and in turn found the *p*-value of statistical significance of this score using package R (Waardenberg et al., 2015).

Finally, using this *p*-value, we discarded all the SNPs the effects of which were estimated as insignificant; otherwise, using decisions on the SNP-caused significant increase and decrease of the binding affinity of TBP for the analyzed promoters, we predicted the candidate SNP markers for over- or underexpression of the appropriate genes, respectively, as demonstrated experimentally (Mogno et al., 2010). Readers can find all our predictions within the columns “K_D_, nM, prediction” of Supplementary Tables S1–S3. Their subcolumns “wt” and “min” contain K_D_ values of TBP’s binding affinity for the ancestral and minor alleles of the appropriate promoters, respectively. Furthermore, subcolumns “Δ” and “α” correspond to the human gene expression alterations and their statistical significance levels α, which are equal to (1 -*p*). In addition, subcolumn “ρ” presents a heuristic rank of our predictions varying in alphabetical order from the “best” (A) to the “worst” (E). Finally, Table 2 contains total numbers of our predictions (N_RES_) as well as the numbers of the candidate SNP markers for either overexpression (N_>_) or underexpression (N_<_) of the human genes, as predicted by this work.

**Table 2:** Predictions of candidate SNP markers that can statistically significantly alter the TATA-binding protein (TBP)-binding sites of the human gene promoters of all the protein-coding transcripts relating to neuropeptidergic, non-neuropeptidergic, and neurotrophinergic systems.

				H_0_: social			H_0_: neutral		
Data studied: GRCh38, dbSNP 150			Result	status equivalence			natural selection		
Human body systems	N_GENE_	N_SNP_	N_RES_	N_↑_	N_↓_	*P*(N_↑_ ≡ N_↓_ ≡ N_RES_/2)	N_>_	N_<_	*P*(N _<_ ≡ 4N _>_ ≡ 4N_RES_/5)
Genome-wide estimate (1000 Genomes Project Consortium et al., 2012)	10^4^	10^5^	1000				200	800	>0.52
Clinical SNP markers of hereditary diseases within the TBP-binding sites (Ponomarenko et al., 2015)	33	203	51				14	37	>0.93
Candidate SNP markers within the TBP-binding sites of promoters of reproductivity-related genes (Chadaeva et al., 2018)	22	129	24				19	5	<0.000001
Candidate SNP markers within the TBP-binding sites of promoters of familial Alzheimer’s disease-related genes (Ponomarenko P. et al., 2017)	5	143	28				16	12	<0.000025
Candidate SNP markers within the TBP-binding sites of promoters of circadian clock core genes (Ponomarenko et al., 2016)	16	162	52				39	13	<0.000001
All: a representative set of genes (Chadaeva et al., 2017)	21	381	92	45	47	>0.9	66	26	<0.000001
Neuropeptidergic	27	395	97	51	46	>0.6	66	31	<0.000001
Non-neuropeptidergic	109	2226	505	240	265	>0.2	342	163	<0.000001
Neurotrophinergic	95	2431	506	265	241	>0.3	346	160	<0.000001
TOTAL	231	5052	1108	556	552	>0.9	754	354	<0.000001

*N_*GENE*_ and N_*SNP*_, total numbers of the human genes and their SNPs (single nucleotide polymorphisms) within the 70 bp promoter region for the protein-coding transcripts, respectively, in this study; N_*RES*_, the total number of the candidate SNP markers predicted in this work that can increase (N_>_) or decrease (N_<_) the TATA-binding protein (TBP) binding affinity for these promoters and, correspondingly, the expression of these genes; N_↑_ and N_↓_, the total numbers of the candidate SNP markers for the human tendencies in dominance and subordination, respectively; *P*(H_*0*_), the estimate of a probability for the acceptance of this H_*0*_ hypothesis, according to the binomial distribution.*

### The Keyword Search in the PubMed Database

**FIGURE 1:**
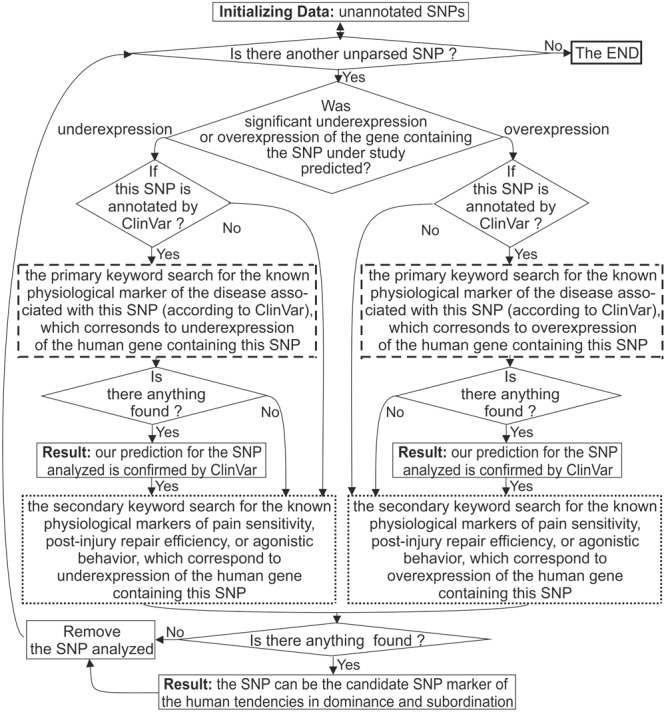
A flow chart of the keyword search for the SNPs of the human neuron-related genes. Dashed boxes depict the primary keyword search for the diseases associated with the analyzed SNP by database ClinVar (Landrum et al., 2014). The dotted boxes depict the secondary keyword search for the known physiological markers of human social behavior, which correspond to the alteration of the gene expression in the case of the SNP being studied.

For each candidate SNP marker predicted, we manually performed a two-step keyword search in the PubMed database (Lu, 2011) as shown in Figure 1.

As presented in this figure, we handled each candidate SNP marker independently of the others, one by one. First of all, we checked whether the SNP in question was annotated by database ClinVar (Landrum et al., 2014) as depicted in Supplementary Figure S1C (hereinafter: see Supplementary File S5 “Supplementary Web service”) and boldfaced in both the first and third rightmost columns of Supplementary Tables S1–S3.

When this database associated the SNP under study with the human diseases, we manually carried out a primary keyword search for the literature data on the known physiological marker of these diseases, which corresponds to the gene expression alteration predicted for this SNP as described elsewhere (Lu, 2011). Figure 1 depicts this procedure as two boxes consisting of dashed lines. In the case of a successful finding of such a publication, the clinical data taken from database ClinVar (Landrum et al., 2014) indicated the adequacy of our predictions for the SNP under consideration. These confirmations of our predictions are *italicized* in both the first and third rightmost column of Supplementary Tables S1–S3.

Finally, two dotted boxes in Figure 1 depict our secondary keyword search for the known physiological markers for pain sensitivity, postinjury repair efficiency, or agonistic behavior, which correspond to underexpression of the human gene containing this SNP. This way, we tested the basic decision-making rule of this work (hereinafter: see subsection “The Basic Decision-Making Rule” “Basic decision-making rule”). As the main bioinformatic results, we predicted the candidate SNP markers for the human tendencies in dominance and subordination, which are in both the first and third rightmost column of Supplementary Tables S1–S3. Table 2 contains the total number of these candidate SNP markers (N_↑_ and N_↓_, respectively).

The section “References” lists the articles cited in Supplementary Tables S1–S3 and in section “Supplementary Method.”

### Statistical Analysis

We analyzed dichotomies via the equiprobable binomial distribution and χ^2^ criteria taken from the standard statistical package Statistica (StatSoft^TM^, Tulsa, United States).

In the genome-wide study *in silico*, using only Fisher’s *Z*-score test, we predicted the candidate SNP markers, the numbers of which for the human gene overexpression and underexpression were compared with one another using the binomial distribution as well as in the case of the human tendencies in dominance and subordination.

During *in vivo* validation in mice, by means of the χ^2^ criterion, we compared the actual numbers of dominants and subordinates among male mice, which were the F1 hybrids of crossing females from inbred strains of an unknown tendency in social hierarchy with males from two inbred strains BALB∖cLac and CBA∖Lac of the previously experimentally identified tendencies in dominance and subordination, respectively (Bragin et al., 2006).

## Results and Discussion

**FIGURE 2:**
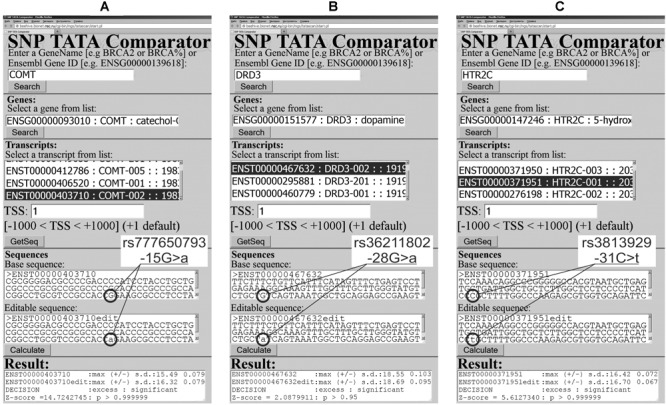
Examples of our predictions in this work in the case of the human genes encoding neuropeptidergic-system-unrelated proteins. **(A)** rs777650793; **(B)** rs36211802; and **(C)** rs3813929.

**FIGURE 3:**
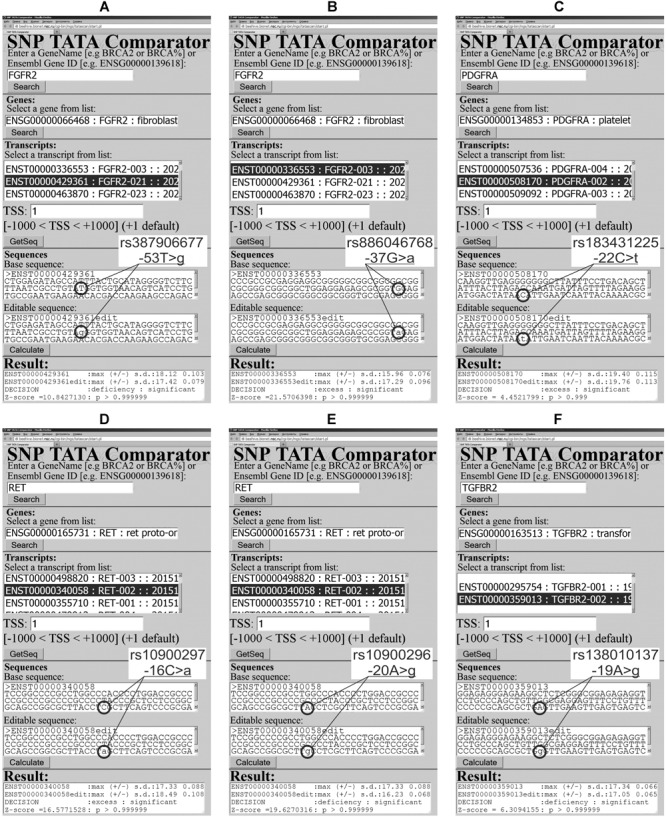
Examples of our predictions in this work in the case of human genes encoding neurotrophinergic-system-related proteins. **(A)** rs387906677; **(B)** rs886046768; **(C)** rs183431225; **(D)** rs10900297; **(E)** rs10900296; and **(F)** rs138010137.

Our analysis of 5052 SNPs of the TBP-binding regions of 231 human neuron-related genes uncovered 1108 candidate SNP markers for the human tendencies in dominance and subordination (Table 2). These predictions are shown in Supplementary Tables S1–S3 and exemplified in Figure 2, 3 and Supplementary Figure S1. For 36 of the 231 genes (16%), namely: *ADRA1B, ADRA2A, ADRA2B, ADRB1, AVP, AVPR1Â, CHRNB2, CNR2, FGF15, FGF16, FGF2, FGF23, FGF7, FIGF, FLT3, GABARAPL3, GABRA3, GABRA4, GABRQ, GMFA, GRIA3, GRIK4, GRIN2B, GRM6, IGF2R, IL27RA, KDR, LIF, MANF, MAOA, MAOB, NGF, OXT, TACR3, TGFBRAP1*, and *VEGFC*, no candidate SNP markers were found (data not shown). Let us focus our analysis of our results on the candidate SNP markers that have independent clinical information within database ClinVar (Landrum et al., 2014) to both verify and discuss their relevance to the human genes under study.

### Candidate SNP Markers Near TBP-Binding Sites in the Promoter of the Human Genes Encoding Neuropeptidergic-System-Related Proteins (e.g., Neurotransmitters)

We applied our experimentally verified public Web service (Ponomarenko et al., 2015) to analyze 395 SNPs in 70 bp proximal promoter regions of 27 human genes encoding neuropeptidergic-system–related proteins, namely: arginine vasopressin receptors (*AVPR*s), C-X-C motif chemokine receptors (*CXCR*s); neuropeptide Y and its receptors (*NPY*s), opioid growth factor receptor (*OGFR*), opioid receptors (*OPR*s), oxytocin and its receptor (*OXT*s), prodynorphin (*PDYN*), proenkephalin (*PENK*), prepronociceptin (*PNOC*), proopiomelanocortin (*POMC*), and tachykinins together with their precursors and receptors (*TAC*s). The results obtained can be found in Supplementary Table S1.

The human *PDYN* gene, i.e., the opioid polypeptide hormone prodynorphin, which is a basic building block of endogenous opioid neuropeptides, so-called endorphins, that can inhibit the pain signals peripherally and cause a feeling of euphoria (when acting in the brain) as neurotransmitters of happiness and joy. SNP rs886056538 of this gene’s promoter was annotated within database ClinVar (Landrum et al., 2014), where it is associated with spinocerebellar ataxia as shown in Supplementary Figure S1C. Supplementary Figure S1D illustrates our prediction for this SNP, which is the line “Decision: excess significant” accompanied by the line “*Z-*score = 2.51, *p* > 0.95” within the textbox “Result.” This outcome means that this SNP can statistically significantly cause overexpression of this gene. Our primary keyword search (hereinafter: two dashed boxes in Figure 1) produced an original experiment (Smeets et al., 2015) involving a mouse model of the human diseases, which has identified the prodynorphin excess as a physiological marker for spinocerebellar ataxia. As one can see, these *in vivo* experimental data independently support our prediction for SNP rs886056538 (Supplementary Figure S1). This observation indicates the suitability of our Web service (Ponomarenko et al., 2015) for computer-based analysis of the human genes encoding neuropeptidergic-system–related proteins as *italicized* in Supplementary Table S1.

After this validation, we manually conducted our secondary keyword search (hereinafter: two dotted boxes in Figure 1) and found the original experiment (Szklarczyk et al., 2012) in a mouse model of human behavior, which associated the prodynorphin excess with reduced conditioned fear. Using our basic decision-making rule within the limitations of the above experimental model of human behavior (Szklarczyk et al., 2012), we predicted that the analyzed SNP rs886056538 can be a candidate SNP marker for the human tendency in dominance (Supplementary Table S1).

Near this clinically characterized SNP marker, we found two unannotated SNPs (rs371345545 and rs557431815), which can also cause overexpression of the human *PDYN* gene (hereinafter: according to our predictions shown in Supplementary Tables S1–S3). That is why we suggest them as two candidate SNP markers of the same genetic tendencies, namely: spinocerebellar ataxia with limitations (Smeets et al., 2015) and social dominance within the framework of the model (Szklarczyk et al., 2012) as presented in Supplementary Table S1.

This way, we predicted 66 and 31 candidate SNP markers for excess and deficiency of the proteins of the human neuropeptidergic system, respectively, which are also 51 and 46 candidate SNP markers predicted by this work for the human tendencies in dominance and subordination (Table 2 and Supplementary Table S1). First of all, readers can see that the numbers of the candidate SNP markers predicted for the human tendencies in dominance and subordination markers are not statistically significantly different from one another according to equiprobable binomial distribution criterion (P(N_↑_ ≡ N_↓_ ≡ N_RES_/2) > 0.6). This finding is in agreement with our preliminary estimate (Chadaeva et al., 2017), namely: P(N_↑_ ≡ N_↓_ ≡ N_RES_/2) > 0.9.

On the contrary, the numbers of the candidate SNP markers predicted for excess and deficiency of the proteins of the human neuropeptidergic system are significantly different from one another according to the equiprobable binomial distribution criterion (P(N _>_ ≡ N _<_ ≡ N_RES_/2) < 0.0005) in line with our preliminary observations (Chadaeva et al., 2017), as presented in Table 2: N > = 66, N < = 26 (P(N _>_ ≡ N _<_ ≡ N_RES_/2) < 0.0005). According to a number of studies, various molecular phenomena can shift frequencies of mutations – e.g., influence of the nucleotide context on the occurrence and repair of pre-mutational damage to genomic DNA, gene conversion, pleiotropic and epistatic effects – Kasowski et al. (2010) first noticed that SNPs decreasing the protein–DNA affinity are much more frequent than SNPs increasing this affinity within the human genome. Next, the authors of ref. (1000 Genomes Project Consortium et al., 2012) quantitatively characterized this mutational shift, namely: there are ∼800 SNPs damaging the transcription factor binding sites and ∼200 SNPs improving these sites per random individual human genome as shown in Table 2. According to Haldane’s dilemma (Haldane, 1957) and neutral evolution theory (Kimura, 1968), this genome-wide estimate can correspond to the neutral mutational drift as a norm. Indeed, we observed 37 clinically proven SNP markers of the human hereditary diseases, which decrease the TBP–promoter affinity, and 14 such SNP markers increasing this affinity (Ponomarenko et al., 2015) in agreement with the above-mentioned genome-wide estimate (Table 2). This pattern matches the commonly accepted opinion on these diseases as a genetic load of the neutral mutational drift in the norm.

Nevertheless, in the case of human reproductive potential, which is considered the target of natural selection, we observed a diametrically opposite pattern, namely: five candidate SNP markers were decreasing the TBP–promoter affinity and 19 candidate SNP markers were increasing this affinity (Chadaeva et al., 2018). Besides, we found (Ponomarenko P. et al., 2017) only a minority (12 of 28) of candidate SNP markers of familial Alzheimer’s disease that can decrease the TBP–promoter affinity; this finding is consistent with natural selection for its very slow pathogenesis, whose clinical manifestation is observed only at the age of over 65 (Table 2). In addition, in the case of core genes of the circadian clock (Ponomarenko et al., 2016), which are naturally selected for continuous coordination between the functioning of systems of the human body and daily fluctuations of the environment, we found 13 candidate SNP markers that can decrease the TBP–promoter affinity and 39 candidate SNP markers increasing this affinity (Table 2).

Looking through Table 2, we noticed that our predictions for the neuropeptidergic gene system are more similar to those for natural selection cases than to those for neutral drift within the normal range. That is why here we predict that the human genes encoding neuropeptidergic-system-related proteins are under natural selection pressure, which equally supports the human tendencies in subordination and domination, as was preliminarily estimated elsewhere (Chadaeva et al., 2017). This way, we followed the semicentennial bioinformatic tradition to compare the actual frequencies of natural mutations within their various dichotomies [e.g., transitions versus transversions (Kimura, 1980) as well as synonymous versus non-synonymous changes (Li et al., 1985)].

### Candidate SNP Markers Near TBP-Binding Sites in the Promoter of the Human Genes Encoding Proteins Related to the Non-neuropeptidergic System (e.g., Receptors)

Using our public Web service (Ponomarenko et al., 2015), we analyzed 2226 SNPs located within the TBP-binding regions of 109 human genes encoding proteins that are related to the non-neuropeptidergic system, e.g., adenosine receptors (*ADOR*s), adrenoceptors (*ADR*s), muscarinic cholinergic receptors (*CHRM*s), nicotinic cholinergic receptors (*CHRN*s), central cannabinoid receptor 1 (*CNR1*), catechol-O-methyltransferase (*COMT*), dopamine D receptors (*DRD*s), GABA type A receptor-associated proteins (*GABARAP*s), γ-aminobutyric acid type B receptor subunits (*GABBR*s), γ-aminobutyric acid - type A receptor subunits (*GABR*s), G protein–coupled receptors (*GRP*s), glutamate ionotropic receptor AMPA–type subunits (*GRIA*s), glutamate ionotropic receptor NMDA-type subunits (*GRIN*s), glutamate metabotropic receptors (*GRM*s), 5-hydroxytryptamine (serotonin) receptors (*HTR*s), dopamine transporter DAT (*SLC6A3*), Na^+^/Cl^-^-dependent serotonin transporter SERT (*SLC6A4*), tyrosine hydroxylase (*TH*), and tryptophan hydroxylase 2 (*TPH2*). Table 2 and Supplementary Table S2 list the results.

The human *COMT* gene for catechol-O-methyltransferase has, in its promoter, a clinically annotated SNP, rs777650793, whose association with human cardiovascular disease was documented by database ClinVar (Landrum et al., 2014). Figure 2A presents our prediction for this SNP, which is an excess of this protein. As a non-statistical validation of this prediction, we manually performed our primary keyword search, which resulted in an experimental study (He et al., 2011) on a rat model of human pathologies, which has identified *COMT* overexpression as a physiological marker of cerebral vasospasm. This correspondence between our prediction (Figure 2A) and these experimental data (He et al., 2011) can support the suitability of the results of our Web service (Ponomarenko et al., 2015) in the case of a study of the human non-neuropeptidergic system as *italicized* in Supplementary Table S2.

As for our secondary keyword search, it resulted in an *in vivo* experiment in a rat model of human behavior (Wilhelm et al., 2013), where a catechol-O-methyltransferase excess was a physiological marker of depression. Within the framework of the behavioral animal model (Wilhelm et al., 2013), we predicted the candidate SNP marker of the human tendency in subordination (Supplementary Table S2).

The human *DRD3* gene (dopamine receptor D3) carries SNP rs36211802 annotated by database ClinVar (Landrum et al., 2014), which associates it with hereditary essential tremor. This SNP can cause an excess of this receptor, according to our prediction given in Figure 2B. We validated this prediction by our primary keyword search, which found the original experimental data (Kosmowska et al., 2016) on resistance to the high-dose DRD3-agonist treatment of tremor in a laboratory rat model of this human pathology as *italicized* in Supplementary Table S2.

In addition, our secondary search revealed (Supplementary Table S2) that a DRD3 excess reduced both motor activity and behavioral motivation in a mouse model of human motor activity (Ikeda et al., 2013). This finding allows us to predict rs36211802 as a candidate SNP marker of the human tendency in subordination (Supplementary Table S2).

The human *HTR2C* gene encodes 5-hydroxytryptamine (serotonin) receptor 2C and carries SNP rs3813929, manifestation of which is an abnormal response to olanzapine (antipsychotic) according to database ClinVar (Landrum et al., 2014). For this SNP, we predict an excess of this serotonin receptor as shown in Figure 2C. Our primary keyword search pointed to the clinical data (Ellingrod et al., 2005) on an HTR2C excess caused by this SNP, whose manifestation is a resistance to olanzapine-caused increase in body mass. It is noteworthy that Tecott et al. (1995) reported that knockout mice (5HT2C^(-)/(-)^) are obese, whereas Stahl (1998) observed eating behavior downregulation with a 5HT2C level increase. With this in mind, our prediction of the rs3813929-related 5HT2C excess (Figure 2C) fits the clinical observation of the rs3813929-related resistance to olanzapine-caused increase in body mass (Ellingrod et al., 2005). This agreement between our prediction shown in Figure 2C and the clinical observations (Tecott et al., 1995; Stahl, 1998; Ellingrod et al., 2005) is consistent with our verification of our predictions of this type by electrophoretic mobility shift assays (EMSAs) under equilibrium (Savinkova et al., 2013) and non-equilibrium conditions (Drachkova et al., 2014) *in vitro*. Besides, this result is in agreement with our verification of our predictions on this subject using biosensor ProteON^TM^ (Bio-Rad Lab, United States) (Drachkova et al., 2012) and stopped-flow spectrometer SX.20 (Applied Photophysics, United Kingdom) (Arkova et al., 2014, 2017) in real-time mode. In addition, it fits our verification of our analogous predictions using human cell lines transfected with the pGL 4.10 vector (Promega, United States) (for a review, Ponomarenko M. et al., 2017). Finally, it is in line with our verification of our predictions on this subject using independent data from 60 experiments (for a review, see Ponomarenko et al., 2010) and by means of 43 known clinical SNP markers of human diseases (Ponomarenko et al., 2009) and 38 known genetic SNP markers of the breeding traits of animals and plants (Suslov et al., 2010). All these verification data can be a reason for the applicability of our Web-service (Ponomarenko et al., 2015) when the human genes relating to the non-neuropeptidergic system are studied, as *italicized* in Supplementary Table S2.

Our secondary keyword search yielded empirical data on two laboratory rat strains, which were bred for 60 generations for the presence and absence of high levels of stress-evoked aggression toward humans (Popova et al., 2010). According to these data, increases in both mRNA and protein levels were seen in the brains of non-aggressive rats in comparison with the aggressive ones (Supplementary Table S2). On this basis, we propose the candidate SNP marker for human tendency in subordination (Supplementary Table S2).

In total, we predicted 342 and 163 candidate SNP markers that can increase and decrease, respectively, the expression of the human proteins related to the non-neuropeptidergic system. Besides, these 505 predictions can be clustered as 240 and 265 candidate SNP markers for the human tendencies in dominance and subordination (Table 2 and Supplementary Table S2). As readers can see in Table 2, these results are again consistent with our preliminary estimates (Chadaeva et al., 2017) that natural selection equally supports the human tendencies in dominance and subordination.

### Candidate SNP Markers Near TBP-Binding Sites in the Promoter of the Human Genes Encoding Neurotrophinergic-System-Related Proteins (e.g., Growth Factors, Receptors)

We applied our public Web service (Ponomarenko et al., 2015) to study 2431 SNPs in 70 bp regions in front of the TSSs of 95 human genes encoding neurotrophinergic-system–related proteins, namely, adenylate cyclase-activating polypeptide 1 and its receptor (*ADCYAP1*s), artemin (*ARTN*), brain-derived neurotrophic factor (*BDNF*), cerebral dopamine neurotrophic factor (*CDNF*), ciliary neurotrophic factor (*CNTF*), fibroblast growth factors and their receptors (*FGF*s), Fms-related tyrosine kinases and their ligand (*FLT*s), glial-cell-derived neurotrophic factor (*GDNF*), GDNF family receptors (*GFR*s), glia maturation factors (*GMF*s), insulin like growth factors and their receptors (*IGF*s), interleukins as well as their receptors and signal transducers (*IL*s), leukemia-inhibitory factor (IL6-family cytokine) and its receptor (*LIF*s), nerve growth factor and its receptor (*NGF*s), neuregulins (*NRG*s), neuropilins (*NRP*s), neurturin (NRTN), neurotrophins (*NTF*s), neurotrophic receptor tyrosine kinases (*NTRK*s), oncostatin M and its receptor (*OSM*s), platelet-derived growth factor subunits and receptors (*PDGF*s), placental growth factor (PGF), persephin (PSPN), Ret receptor tyrosine kinase (*RET*), transforming growth factors β, its receptors and associated protein 1 (*TGFB*s), and vascular endothelial growth factors (*VEGF*s). We show our results in Table 2 and Supplementary Table S3.

The human *FGFR2* gene (fibroblast growth factor receptor 2) contains two SNPs rs387906677 and rs886046768, which were clinically detected in patients with bent bone dysplasia syndrome and craniosynostosis, respectively, as documented by database ClinVar (Landrum et al., 2014). Readers can see in Figure 2B, 3A how we predicted the FGFR2 deficiency in the case of rs387906677, whereas rs886046768 corresponds to an FGFR2 excess.

At first, our primary keyword search revealed an experimental report (Merrill et al., 2012) on a mouse model of human embryonic development, which linked bent bone dysplasia with reduced levels of FGFR2. Next, in the same way, we found the original experiment (Mansukhani et al., 2000) on mouse osteoblast cell culture *ex vivo* that points to FGFR2 as an inducer of apoptosis in these cells and an inhibitor of their differentiation, hyperactivity of which causes craniosynostosis-linked alterations in cell culture. As depicted in the figures, these independent findings confirm the validity of our predictions (Figure 3A,B) in the case of the neurotrophinergic system analysis, as *italicized* in Supplementary Table S3.

After this validation, our secondary keyword search yielded an article (Meyer et al., 2012) on FGFR2 deficiency as a physiological marker of delayed post-injury skin wound healing. Analogously, we found a biomedical paper (Baatar et al., 2002) on the injections of recombinant human FGFR2 around ulcers, which have accelerated ulcer healing in rats as an animal model of the human pathologies. On this basis, we predicted rs387906677 and rs886046768 as candidate SNP markers of the human tendencies in subordination and dominance, respectively (Supplementary Table S3).

The human *PDGFRA* gene encodes platelet-derived growth factor receptor α and contains SNP rs183431225 annotated by database ClinVar (Landrum et al., 2014) in connection with both idiopathic hypereosinophilic syndrome and gastrointestinal stromal tumor. Figure 3C presents our prediction for this SNP: overexpression of this receptor. Our primary keyword search revealed two biomedical papers, one of which (Score et al., 2006) reports the PDGFRA excess as a marker of patients with hypereosinophilia, and another one (Hayashi et al., 2015) reveals reduced proliferation of gastrointestinal stromal tumor cells under the influence of a selective inhibitor of PDGFRA. Thus, these independent literature data support applicability of our predictions to the study of human genes encoding neurotrophinergic-system–related proteins as *italicized* in Supplementary Table S3.

Then, we did our secondary keyword search and found a mouse model of human behavior indicating that the PDGFRA overexpression causes oligodendrocyte-associated nociceptive hypersensitivity to neuropathic pain (Shi et al., 2016). That is why we assumed that rs183431225 is a candidate SNP marker of the human tendency in subordination (Supplementary Table S3).

The human *RET* gene codes for the Ret proto-oncogene, where two SNPs (rs10900297 and rs10900296) have been associated with three human diseases (renal adysplasia, Hirschsprung disease, and pheochromocytoma) as documented in database ClinVar (Landrum et al., 2014). As readers can see in Figure 3D,E, our predictions for these SNPs surprisingly correspond to over- and underexpression of this gene. Nevertheless, using our primary keyword search, we learned that both an excess (Sarin et al., 2014) and deficit (Bridgewater et al., 2008) of RET are known as physiological markers of renal adysplasia. In addition, both overexpression (Ishii et al., 2013) and underexpression (Zhan et al., 1999) of the *RET* gene can contribute to the pathogenesis of Hirschsprung disease. Finally, both increased (Huang et al., 2003) and decreased (Moore and Zaahl, 2012) levels of this proto-oncogene are often seen in pheochromocytoma. Thus, the above publications additionally validate our results (Figure 3D,E) as *italicized* in Supplementary Table S3.

Accordingly, we conducted a secondary keyword search and thus selected two animal models of human behavior. The rat model (Wang et al., 2017) associated the RET excess with hypersensitivity to neuropathic pain. In the mouse model (Golden et al., 2010), the RET deficit reduced epidermal innervation. Within the limitations of these models, we predicted two candidate SNP markers (rs10900297 and rs10900296) of the human tendency in subordination (Supplementary Table S3).

The human *TGFBR2* gene (transforming growth factor β receptor 2) contains SNP rs138010137, which occurs in patients with thoracic aortic aneurysm as documented in database ClinVar (Landrum et al., 2014). According to our prediction illustrated in Figure 3F, this SNP can reduce levels of receptor TGFBR2 in humans. Using a primary keyword search, we found an original work about the TGFBR2-deficient aortic aneurysm and aortic dissection as the specific forms of these pathologies (Angelov et al., 2017). As one can see, this is one more argument in favor of the applicability of our Web service (Ponomarenko et al., 2015) to research on the human genes related to the neurotrophinergic system as *italicized* in Supplementary Table S3.

Next, our secondary keyword search yielded a transgenic mouse model of human health (Martinez-Ferrer et al., 2010), in which the TGFBR2 deficit accelerates healing, closure, and resurfacing of skin wounds. For this reason, we suggest rs138010137 as a candidate SNP marker of the human tendency in dominance (Supplementary Table S3).

Summarizing all the above, we can see 506 candidate SNP markers predicted by this work in the case of human genes encoding the neurotrophinergic-system-related proteins (Table 2 and Supplementary Table S3). These predictions can be grouped into 346 and 160 candidate SNP markers of the excess and deficiency of these proteins, respectively, as well as into 265 and 241 candidate SNP markers of the human tendencies in dominance and subordination (Table 2). Notably, the first of these dichotomies of SNPs in the human genome is statistically significantly uneven, whereas the second one is uniform. This is one more actual piece of evidence for the pressure of natural selection on the human neuron-specific genes, which equally supports the human tendencies in dominance and subordination, in agreement with our preliminary estimates (Chadaeva et al., 2017) as well as with all the other predictions of this work.

### *In silico* Validation of All the Genome-Wide Predictions Made in This Work

Altogether, we analyzed 5052 SNPs within all the TBP-binding regions of all the promoters in front of all the protein-coding transcripts of all the 231 known human neuron-specific genes and selected 1108 candidate SNP markers that can significantly affect the affinity of TBP for these promoters (22%) as shown in the bottom row of Table 2. This result of our exhaustive whole-genome analysis of three systems of the human body (neuropeptidergic, non-neuropeptidergic, and neurotrophinergic) is consistent with both Haldane’s dilemma (Haldane, 1957) and Kimura’s neutral evolution theory (Kimura, 1968). Our *in silico* fivefold reduction in the number of unannotated SNPs for their subsequent *in vivo* studies is in line with the current need for reducing the cost of both experimental and clinical searches for valuable SNP markers in the human genome by trial and error through preliminary computer analysis of the known SNPs (Deplancke et al., 2016).

With this in mind, we selected all the 10 among 1,108 candidate SNP markers predicted in this work (Figure 2, 3 and Supplementary Figure S1), which are currently linked to the human diseases by public database ClinVar (Landrum et al., 2014). As described above, we non-statistically validated this set of our selected predictions by our primary keyword search in the public PubMed database (Lu, 2011). Essentially, this match between our 10 selected predictions and the found literature data is statistically significant at the level of α < 0.001 according to the criterion of the equiprobable binomial distribution.

It is important to note that most of the candidate SNP markers that were marked in database ClinVar (Landrum et al., 2014) had a “Clinically insignificant” label because the number of patients with these candidate SNP markers varied from one to six, whereas for clinical significance it is necessary to use cohorts of several hundred patients. This observation supports subsequent verification (using clinical protocols) of the candidate SNP markers predicted by this work. In this way, genotyping for the elite combat athletes in addition to the widely used textual psychological questionnaires for them (Tiric-Campara et al., 2012) could enrich personalized sports medicine.

In addition, we used the semicentennial bioinformatic tradition of comparing the actual frequencies of mutations for their various dichotomies [transitions versus transversions (Kimura, 1980), synonymous versus non-synonymous changes (Li et al., 1985), etc.]. To this end, we grouped all the 1108 predictions into 754 and 354 candidate SNP markers for the increase and decrease in the TBP binding affinity for promoters of the human neuron-related proteins, respectively (Table 2: N_RES_, N_>_ and N_<_). This dichotomy contradicts the binomial distribution of the whole-genome ratio 4:1 of the SNPs reducing versus SNPs increasing affinity of the transcription factors for the human gene promoters (1000 Genomes Project Consortium et al., 2012) as neutral drift according to Haldane’s dilemma (Haldane, 1957) and neutral evolution theory (Kimura, 1968), Table 2: p(N _<_ = 4N _>_ = 4N_RES_/5) < 0.000001. This significant contradiction means the adaptive pressure of natural selection on the human neuron-specific genes is in line with the commonly accepted opinion about the adaptive role of both the nervous system and social behavior in the course of human origin and evolution. That is one more evolutionary argument for the reliability of our predictions made in this work.

Finally, by the same reasoning, we grouped all the 1,108 predictions into 556 and 552 candidate SNP markers for the human tendencies in dominance and subordination, respectively (Table 2: N_RES_, N_↑_, and N_↓_). In contrast to the above dichotomy, this one corresponds to the highly probable H_0_ hypothesis about the equiprobable binomial distribution of these candidate SNP markers for human social hierarchy [Table 2: p(H_0_: N_↑_ = N_↓_ = N_RES_/2) > 0.9]. This correspondence means that the pressure of natural selection proven above equally supports the human tendencies in dominance and subordination.

Notably, so that natural selection can control the human tendencies in dominance and subordination, it is necessary that this human tendencies can be inherited from generation to generation from parents to offspring. That is why, we *in vivo* validated our *in silico* predictions of this work in a mouse model of human inheritance as described below.

### *In vivo* Validation of Our Predictions Using a Mouse Model of Human Inheritance

**FIGURE 4:**
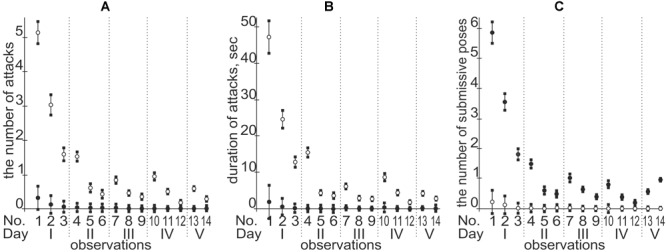
A temporal pattern of both formation and maintenance of the social hierarchy in mouse pairs. Legend: ∘ and •, dominant and subordinate male mice, respectively; **(A)** the number of attacks; **(B)** duration of attacks (second); **(C)** the number of submissive poses; the circle and error bar denote the arithmetic mean and SD for 115 observations, respectively.

Each public Web service addresses a specific sort of regulatory SNP analysis (e.g., Bendl et al., 2016), and each has its specific advantages and disadvantages. Therefore, a comparison between the particular predictions and experimental data as an independent commonly accepted uniform platform (rather than between predictions of various Web services) needs to be a necessary step for prediction of candidate SNP markers *in silico* (Yoo et al., 2015; Ponomarenko M. et al., 2017). Keeping this in mind, we *in vivo* validated our *in silico* predictions on the equal natural-selection support of the human tendencies in dominance and subordination using a mouse model of human inheritance as described in the section “Materials and Methods.” The obtained results are given in Figure 4 and Table 3.

**Table 3:** The results of identification of inheritance of the murine tendencies in dominance and subordination.

Paternal genotype Maternal genotype	BALB/cLac	CBA/Lac	*χ*^2^ criterion	
			*χ*^2^	Significance, α
**PT**	**21**	**10**	**3**.**90**	**0**.**05**
**DD**	**16**	**4**	**7**.**20**	**0**.**01**
C57BL/6J	13	7	1.80	>0.1
**YT**	**16**	**5**	**5**.**76**	**0**.**025**
A/He	13	10	0.39	>0.5
**TOTAL**	**79**	**36**	**16**.**08**	**0**.**001**

*The number of male mice – that dominated over their neighbors within the framework of their pair – is indicated; statistically significant results are boldfaced.*

Figure 4 indicates that we completely reproduced the temporal pattern of both formation and maintenance of the social hierarchy in mouse pairs by means of both the number and duration of attacks and submissive poses.

As one can see in the first row “PT” of this table, 21 of 31 mouse males of the F1 hybrids carrying the PT × BALB∖cLac genotype dominated over the male F1 hybrids of the PT × CBA/Lac genotype, and 10 mouse males of the PT × CBA/Lac genotype were dominant in the remaining pairs of the same combination. This actual difference between the F1 male hybrids PT × BALB/cLac and PT × CBA∖Lac is characterized by the χ^2^-score equal to 3.9, which is statistically significant at the level of α < 0.05. In addition, we observed the same significant dominance of the BALB/cLac-related F1 hybrids over the CBA/Lac-related ones, when the maternal inbred strains were DD and YP (Table 3). In addition, in the cases of maternal inbred strains C57BL/6J and A/He, we found only a tendency for the same dominance, which was insignificant, possibly because of the insufficient number of the appropriate mouse male pairs studied regarding these maternal genotypes. Finally, the last row of Table 3 represents the final result: the statistically significant majority of 79 among 115 BALB/cLac-related male hybrids achieved their dominant social status within their pairs with the CBA∖Lac-related males of the same maternal inbred strains. This finding means that this mouse model of human inheritance reveals an ability of the tendencies in dominance and subordination to be inherited from generation to generation from parents to offspring and, therefore, to be an object of natural selection. This is the main genetic *in vivo* argument in favor of the reliability of our *in silico* predictions in this work.

Finally, looking through Figure 4, one can see that, in contrast to the first day of microsocial observation of a pair of adult male mice, which was characterized by numerous and lasting attacks of one mouse on the other, by the end of the second day a social hierarchy is established, with rare short-term ritualized attacks of dominant and/or ritualized submissive poses of a subordinate without any injuries and dangers for their lives and health (Lorenz, 2002). This is the main ecological benefit of establishing and maintaining social hierarchy, as a result of which natural selection equally supports the human tendencies for both dominance and subordination.

## Conclusion

In this work, we analyzed only how SNPs can alter TBP’s binding affinity for the human gene promoters, whereas more than 2500 human DNA-binding proteins are already known (Babu et al., 2004). Consequently, now there is a huge variety of Web services for studying the effects of SNPs on the binding affinity of the human gene promoters for these proteins and the respective phenotypic manifestations (e.g., Bendl et al., 2016). Their use can significantly expand the research capabilities in comparison with the use of our Web service alone (Ponomarenko et al., 2015).

The main finding of this work is that natural selection equally supports the human tendencies in dominance and subordination, which can be inherited from parents to offspring. The results of current study could be seen as an argument in favor of the genetic side within the century-old irreconcilable scientific debate on the nature of both aggressiveness and social hierarchy in humans [e.g., Freud (1921, 1930) and Lorenz (1964, 2002)]. Nevertheless, in the case of a random individual, these human tendencies can define the possible ranges (plasticity) of his/her aggressiveness and social rank rather that their actual levels, which depend on his/her continuous non-genetic social education from childhood to the oldest age (Markel, 2016). Certainly, this one is an argument in favor the other (non-genetic) side of the debate in question [e.g., Fromm (1941, 1973), Berkowitz (1962, 1993), Skinner (Rogers and Skinner, 1956; Skinner, 1981)]. According to recent reports on epigenetics (e.g., Merkulov et al., 2017), various stressors may cause epigenetic reprogramming of the individual genome and, in this way, modulate the actual levels of both individual aggressiveness and social status. Moreover, this reprogrammed pattern of the human genome is inherited from parents to offspring across at least two generations. Definitely, this notion equally supports both sides of the above debate as does our main finding in this work.

Finally, there are social mechanisms of transfer of the hierarchy status from parents to their offspring, previously described in macaques (Prud’Homme and Chapais, 1993), deer (Dusek et al., 2007), and hyenas (Engh et al., 2000). Clearly, the real effects of inherited genotypes on the human social hierarchy are much more complex, diverse, richer, brighter, and more interesting than our maximally simplified decision-making rule (see subsection “The Basic Decision-Making Rule” “Basic decision-making rule”). Nevertheless, at least a somewhat valid decision-making rule is necessary for application of the bioinformatic calculations to the genome-wide analysis *in silico*. In any case, as a computer-based prediction, each candidate SNP marker of the human tendencies in dominance and subordination predicted by this work should be experimentally verified in the studies of large human cohorts.

## Author Contributions

NK contributed to concept. DR and PP contributed to software. IC contributed to data compilation. ES and LS contributed to data analysis. MK, EK, LO, and AO performed the *in vivo* experiment. MP wrote the manuscript. VN performed the revised manuscript study design.

## Conflict of Interest Statement

The authors declare that the research was conducted in the absence of any commercial or financial relationships that could be construed as a potential conflict of interest.

## Footnotes

1 http://beehive.bionet.nsc.ru/cgi-bin/mgs/tatascan/start.pl

1 https://grants.nih.gov/grants/olaw/National_Academies_Guidelines_for_Use_and_Care.pdf
