# Supplementary material for: Natural Selection Equally Supports the Human Tendencies in Subordination and Domination: A Genome-Wide Study With in silico Confirmation and in vivo Validation in Mice
Source: Front Genet. 2019 Feb 20;10:73. doi: 10.3389/fgene.2019.00073 (PMC6404730; doi:10.3389/fgene.2019.00073)
Supplement: Supplementary file 1 [file Data_Sheet_1.PDF]

frontiers

Supplementary Material

Natural selection equally supports the human tendencies in subordination and domination: a genome-wide study with *in silico* confirmation and *in vivo* validation in mice

Irina Chadaeva, Petr Ponomarenko, Dmitry Rasskazov, Ekaterina Sharypova, Elena Kashina, Maxim Kleshchev, Mikhail Ponomarenko*, Vladimir Naumenko, Ludmila Savinkova, Nikolay Kolchanov, Ludmila Osadchuk, Alexandr Osadchuk

***Correspondence:** Mikhail Ponomarenko (pon@bionet.nsc.ru)

Table S1. Candidate SNP markers predicted in this work near TBP-binding sites in the promoter of the human genes encoding neuropeptidergic-system–related proteins (e.g., precursors, neurotransmitters, receptors)

Gene, OMIM	dbSNP (Sherry et al., 2001)	5′ flank	wt	**mut**	3′ flank	K_D, nM, prediction						**Known physiological or** *candidate SNP markers*	Ss	**ClinVar** or *Reference*
						wt	**mut**	Δ	Z	α	ρ			
AVPR1A, 600821	rs576256624	gcgggacgct	*g*	**a**	ctccgggaga	*133*	**96**	>	6	10^{-6}	A	increased depression-like behavior	↓	Wang et al., 2008
	rs780371622	ctggggttct	*c*	**t*)**	gtgcctacgt	*30*	**15**	>	11	10^{-6}	A		↓	
AVPR2, 300538	rs782737290	gcccaggact	*g*	**a**	gccatactgc	*32*	**26**	>	3	10^{-2}	C	reduced tolerant behavior	↑	Wiren et al., 2013
	rs782247560	tctataaggg	*c*	**t**	tccagtccag	*6*	**7**	<	2	0.05	D	increased tolerant behavior	↓	
CXCR4, 162643	rs902195703	gagcgtgttt	*t*	**a**	tataaaagtc	*2*	**1**	>	7	10^{-6}	A	increased sensitivity to inflammatory pain	↓	Yang et al., 2015
	rs988917830	gcctctttgt	*g*	**a**	tgtatttttt	*7*	**4**	>	10	10^{-6}	A		↓	
	rs561969013	tggcctcttt	*g*	**t**	tgtgtatttt	*7*	**6**	>	4	10^{-3}	B		↓	
	rs575012225	gtgtttttat	*a*	**g**	aaagtccggc	*2*	**5**	<	13	10^{-6}	A	reduced behavioral motor coordination and balance	↓	Huang et al., 2014
NPY, 162640	rs139801169	ccgcaaggtg	*g*	**a, t**	tgctagccac	*89*	**61**	>	7	10^{-6}	A	low innate fear response as post-injury stress behavior	↑	Ragu Varman, Rajan, 2015
	rs1046716779	agcgtgactg	*c*	**a**	ccgaggcccc	*56*	**62**	<	2	0.05	D	increased behavioral despair linked to mood disorders	↓	Aoki et al., 2016
	rs528363143	tagccactcc	*t*	**c**	gggttctctc	*89*	**102**	<	3	0.05	D		↓	
	rs893676518	gaagctccat	*a*	**–**	aaagccctgt	*6*	**10**	<	10	10^{-6}	A		↓	
NPY1R, 162641	rs532918517	agtggcaccg	*a*	**g**	ctttttcaag	*24*	**21**	>	3	10^{-2}	C	increased fear behavior as post-injury stress disorders	↓	Hendriksen et al., 2012
	rs773501686	agagttattt	–	**attt**	gagcgtcaca	*9*	**5**	>	9	10^{-6}	A		↓	
	rs867271612	cgggatggca	*c*	**t**	acacaggcag	*23*	**13**	>	7	10^{-6}	A		↓	
	rs1009832720	caaattctaa	*c*	**a**	agcagagttt	*18*	**10**	>	8	10^{-6}	A		↓	
	rs1987077	accctccctt	*c*	**t**	caaattctaa	*18*	**10**	>	10	10^{-6}	A		↓	
	rs568594705	agttatttga	*g*	**a**	cgtcacaaaa	*9*	**12**	<	3	10^{-2}	C	increased anxiety-related behavior	↑	Longo et al., 2014
	rs202142221	gtgagagtta	*tttg*	**–**	agcgtcacaa	*9*	**18**	<	10	10^{-6}	A		↑	
	rs138613932	cgtgagagtt	*attt*	**–**	gagcgtcaca	*9*	**17**	<	10	10^{-6}	A		↑	
	rs200304866	gtgagagtta	*t*	**g**	ttgagcgtca	*9*	**16**	<	7	10^{-6}	A		↑	
	rs13447344	ggcgtgagag	*ttat*	**–**	ttgagcgtca	*9*	**17**	<	10	10^{-6}	A		↑	
	rs201866126	cgtgagagtt	*a*	**g**	tttgagcgtc	*9*	**17**	<	10	10^{-6}	A		↑	
	rs34284074	aggcagggga	–	**c**	catgtggagc	*23*	**27**	<	2	0.05	D		↑	

Notes: hereinafter, Alleles: wt, ancestral; mut, minor; "–", deletion; K_D, dissociation constant of TBP–DNA complex; α = 1 – p, significance (where p value is given in Figure 1); **Δ, changes**: excess (>) and deficit (<); **Ss, Social status**: dominance (↑) and subordination (↓); ρ, heuristic rank of candidate SNP markers varying in alphabetical order from the "best" (A) to the "worst" (E). *)This SNP also includes other neutral alleles. #)Distance from this SNP to a given alternative transcription start site (TSS) whose transcription activity is altered by this SNP. ClinVar, the database of clinical annotations of SNPs (Landrum et al., 2014); Reference, the *italicized references* found by our manual keyword search in the PubMed database (Figure S1, Supplementary file 1) the contents of which are *italicized* in the third rightmost column. **Genes**: *AVPR*s, arginine vasopressin receptors; CXCRs, C-X-C motif chemokine receptors; NPYs, neuropeptide Y and its receptors; *OGFR*, opioid growth factor receptor; *OPR*s, opioid receptors; *OXT*s, oxytocin and its receptor; *PDYN*, prodynorphin; *PENK*, proenkephalin; *PNOC*, prepronociceptin; *POMC*, proopiomelanocortin; *TAC*s, tachykinins as well as their precursors and receptors. **Deletion/insertion**, TAC4: 10 bp = gagaggggct.

Supplementary Material

Table S1. Continued

Gene, *OMIM*	dbSNP (Sherry et al., 2001)	5′ flank	wt	**mut**	3′ flank	K_D, nM, prediction: wt	**mut**	Δ	Z	α	ρ	**Known physiological or** candidate SNP markers	Ss	**ClinVar** or Reference
NPY2R, 162642	rs570646284	tctgggtagg	*g*	**a**	tctggctgag	*53*	**38**	>	5	10^{-6}	A	increased susceptibility to acute pain	↓	Arcourt et al., 2017
	rs796119959	accaaaactt	*ctc*	**–**	ctccagtccc	*79*	**46**	>	11	10^{-6}	A		↓	
NPY4R, 601790	rs28779793	caggcagcat	*c*	**a**	ggggcagcag	*55*	**38**	>	6	10^{-6}	A	reduced long-term anxiety-like behavior	↓	Painsipp et al., 2010
NPY5R, 602001	rs1026079743	ggcctgcacc	*g*	**a**	aggggccgtg	*128*	**66**	>	11	10^{-6}	A	increased anxiolytic behavior	↓	Sajdyk et al., 2002
	rs1013398651	gtaggcctgc	–	**a**	accgaggggc	*128*	**102**	>	3	10^{-3}	B		↓	
	rs896300674	gggtaggcct	*g*	**a**	caccgagggg	*128*	**31**	>	16	10^{-6}	A		↓	
	rs149127945	ccgcctccag	*g*	**a**	tcctgctccc	*111*	**64**	>	11	10^{-6}	A		↓	
	rs1026006586	ccctcttccc	*c*	**t**	accgccgcct	*111*	**55**	>	12	10^{-6}	A		↓	
OGFR, 606459	rs780240226	cctctgcttt	*c*	**a**	ggtttcgctt	*74*	**20**	>	23	10^{-6}	A	slow wound healing	↓	McLaughlin et al., 2012
	rs905576745	cccgcgcttc	*c*	**g**	tctgctttcg	*74*	**59**	>	4	10^{-3}	B		↓	
	rs115849595	tggggtctgg	*c*	**t**	tctgtcccac	*56*	**44**	>	5	10^{-3}	B		↓	
	rs565921570	ccccagggga	*g*	**a**	gtgcatgtca	*31*	**27**	>	3	10^{-2}	C		↓	
	rs530369219	ggggaggtgc	*a*	**c**	tgtcagccag	*31*	**37**	<	3	10^{-2}	C	fast wound healing	↑	
OPRD1, 165195	rs934282119	ccacgtggtg	*c*	**t**	gcgcggcggg	*64*	**51**	>	4	10^{-3}	B	offspring impulsive behavior during maternal high-fat diet	↑	Grissom et al., 2015
OPRK1, 165196	rs889475364:**t**	cgcaccagac	*g*	**t**	agctctccgc	*86*	**44**	>	11	10^{-6}	A	reduced sexual/agonistic aggressive male behaviors	↓	Riters et al., 2017
	rs889475364:**c**	cgcaccagac	*g*	**c**	agctctccgc	*86*	**101**	<	3	10^{-2}	C	increased sexual/agonistic aggressive male behaviors	↑	
OPRL1, 602548	rs955236849	gagtcgaggt	*g*	**t**	ctcatagtgg	*16*	**8**	>	10	10^{-6}	A	reduced fear behavior as post-traumatic stress disorder	↑	Andero et al., 2013
	rs544515289	gaggtgctca	*g*	**a**	agtggagccc	*16*	**52**	<	18	10^{-6}	A	increased fear behavior as post-traumatic stress disorder	↓	
OPRM1, 600018	rs201738594	ctcctgcagc	*g*	**a**	gtgcggggca	*114*	**80**	>	7	10^{-6}	A	increased voluntary locomotor motivation behavior	↑	Ruegsegger et al., 2015
	rs779909053	ccctcctgca	*g*	**a**	cggtgcgggg	*114*	**75**	>	8	10^{-6}	A		↑	
	rs199671121	catggcccac	*g*	**a**	ctcccctcct	*114*	**100**	>	2	0.05	D		↑	
	rs577434238	tccgaatccc	*g*	**a**$^{*)}$	catggcccac	*114*	**63**	>	11	10^{-6}	A		↑	
	rs199622216	agcctccgaa	*t*	**a**	cccgcatggc	*114*	**102**	>	2	0.05	D		↑	
	rs201221097	ttccagcctc	*c*	**t**$^{*)}$	gaatcccgca	*114*	**51**	>	15	10^{-6}	A		↑	
	rs754614875	cttggaaccc	*g*	**t**$^{*)}$	aaaagtctcg	*81*	**64**	>	4	10^{-3}	B		↑	
	rs201796075	tggcagcggc	*g*	**a**	aaaggaagcg	*81*	**61**	>	5	10^{-6}	A		↑	
	rs776522590	actggtttct	*g*	**a**	taagaaacag	*9*	**3**	>	16	10^{-6}	A		↑	
	rs918589189	gtatatttac	*c*	**t**	atatacaaca	*3*	**1**	>	8	10^{-6}	A		↑	
	rs17174638:61$^{\#)}$	ctgtaagaaa	*c*	**t**	agcaggagct	*81*	**63**	>	4	10^{-3}	B		↑	
	rs17174638:22$^{\#)}$	ctgtaagaaa	*c*	**t**	agcaggagct	*9*	**11**	<	2	0.05	D	reduced voluntary locomotor motivation behavior	↓	Ruegsegger et al., 2016
	rs114622967	cccctcctgc	*a*	**c**	gcggtgcggg	*114*	**136**	<	3	10^{-3}	B		↓	
	rs781653758	tctgtgaact	*a*	**g**	ctaaggtggg	*5*	**26**	<	27	10^{-6}	A		↓	
	rs200808543	gcctctgtga	*a*	**g**	ctactaaggt	*5*	**6**	<	4	10^{-3}	B		↓	
	rs1033909721	aagaaacagc	*a*	**g**	ggagctgtgg	*81*	**102**	<	4	10^{-3}	B		↓	
	rs1038298587	atgtatattt	*a*	**g**	ccatatacaa	*2.7*	**3.2**	<	2	0.05	D		↓	
OXTR, 167055	rs1031901090	cgcggcaggt	*g*	**a**	gatatgctga	*19*	**4**	>	21	10^{-6}	A	increased maternal aggressive behavior	↑	Naik, de Jong, 2017
	rs4564970	ggtcccgcgg	*c*	**t**$^{*)}$	aggtggatat	*19*	**15**	>	4	10^{-3}	B		↑	
	rs936633981	ccgcggcagg	*t*	**g**	ggatatgctg	*19*	**22**	<	2	0.05	D	increased intermale aggressive behavior	↑	Caldwell et al., 2017

Supplementary Material

Table S1. Continued

Gene, *OMIM*	dbSNP (Sherry et al., 2001)	5′ flank	wt	**mut**	3′ flank	K_D, nM, prediction: wt	**mut**	Δ	Z	α	ρ	**Known physiological or** candidate SNP markers	Ss	**ClinVar** or Reference
PDYN, 131340	rs886056538	tgctcagcaa	*g*	**a, c**	ggctgagcga	*107*	**94**	>	3	0.05	D	**spinocerebellar ataxia**	↑	**Landrum et al., 2014**
	rs371345545	agggctgagc	*g*	**a**	acaggggagg	*107*	**85**	>	5	10^{-3}	B	*predisposition to spinocerebellar ataxia* as well as reduced conditioned fear behavior	↑	*Smeets et al., 2015;* as well as Szklarczyk et al., 2012
	rs557431815	aagggctgag	*c*	**t**	gacaggggag	*107*	**59**	>	12	10^{-6}	A		↑	
PENK, 131330	rs866088096	cgaaggcgtc	*g*	**a**	gcgcggggct	*213*	**175**	>	4	10^{-3}	B	reduced conditioned fear behavior	↑	Szklarczyk et al., 2015
	rs991109165	ctgggggacc	*g*	**t**	acccctcccg	*213*	**61**	>	19	10^{-6}	A		↑	
	rs916429832	cgggtgctgg	*g*	**a**	ggaccgaccc	*213*	**189**	>	2	0.05	D		↑	
	rs949176062	gcgggtgctg	*g*	**a**	gggaccgacc	*213*	**165**	>	5	10^{-6}	A		↑	
	rs1046203213	agcggcgcgc	*g*	**t*)**	ggtgctgggg	*213*	**153**	>	6	10^{-6}	A		↑	
	rs996793753	cttcggtttg	*c*	**t**	ggctaattat	*29*	**22**	>	4	10^{-3}	B		↑	
	rs756068403	cctctctcct	*g*	**a, t**	gcagtccatg	*85*	**62**	>	6	10^{-6}	A		↑	
	rs745897254	aggcaggctc	*g*	**a**	aagcacgcgt	*85*	**50**	>	11	10^{-6}	A		↑	
	rs775737775	ccaggcaggc	*t*	**a**	cgaagcacgc	*85*	**51**	>	10	10^{-6}	A		↑	
	rs777120231	accccgttct	*g*	**a*)**	cgcccaggca	*85*	**36**	>	14	10^{-6}	A		↑	
	rs949123326	aggcaggcgc	*t*	**a**	cagagccccg	*84*	**63**	>	5	10^{-3}	B		↑	
	rs899413421	gggctaatta	*t*	**c**	aaagtggctc	*3*	**6**	<	11	10^{-6}	A	reduced depression-like behavior	↑	Melo et al., 2014
	rs903665007	cgtccctctc	*t*	**c**	cctcgcagtc	*85*	**106**	<	4	10^{-3}	B		↑	
PNOC, 601459	rs535246928	ggtctgatgg	*c*	**t**	agagccgggg	*40*	**23**	>	9	10^{-6}	A	increased pain sensitivity	↓	Kawashima et al., 2002
	rs567831289	gtggggatgt	*c*	**t**	acaggtctga	*40*	**12**	>	15	10^{-6}	A		↓	
	rs189847007	cttttttttct	*g*	**t**	acttgccaga	*23*	**11**	>	16	10^{-6}	A		↓	
	rs879443789	tttgactaca	*t*	**a**	actttcacat	*5*	**21**	<	14	10^{-6}	A	reduced pain sensitivity	↑	
POMC, 176830	rs894296524	ccctttccag	*c*	**t**	gcgtctcccc	*93*	**81**	>	2	0.5	D	reduced maternal aggressive behavior	↓	Saltzman et al., 2011
	rs573894982	cggcaagtat	*a*	**g**	taaggacaga	*1*	**2**	<	13	10^{-6}	A	increased acute aggressive response to social conflicts	↑	Vaanholt et al., 2003
	rs957071370	ctcggcaagt	*a*	**g**	tataaggaca	*1*	**3**	<	16	10^{-6}	A		↑	
	rs746793223	gctcggcaag	*t*	**c**	atataaggac	*1*	**3**	<	16	10^{-6}	A		↑	
	rs989925527	agctcggcaa	*g*	**a**	tatataagga	*0.9*	**1.2**	<	4	10^{-3}	B		↑	
TAC1, 162320	rs906048828	gggataaata	*c*	**a**	cgcaaggcac	*4*	**5**	<	2	0.05	D	reduced fear behaviors	↑	Bilkei-Gorzo et al., 2002
TAC3, 162330	rs961685802	ttggttttct	*g*	**a*)**	ttccctctcc	*24*	**11**	>	14	10^{-6}	A	increased grooming behavior	↓	Picard et al., 1994
	rs760324417	acttggtttt	*c*	**t**	tgttccctct	*24*	**19**	>	5	10^{-3}	B		↓	
	rs763817650	atgggcacta	*g*	**t**	acttggtttt	*24*	**7**	>	16	10^{-6}	A		↓	
	rs753615797	gggatgggca	*c*	**t*)**	tagacttggt	*24*	**19**	>	5	10^{-3}	B		↓	
TAC4, 607833	rs536155323	ggctcccaga	*g*	**a, t**	attccaggac	*37*	**29**	>	5	10^{-3}	B	increased anxiolytic and antidepressant-like behavior	↑	Borbely et al., 2017
	rs766654525	cttcaagctg	*a*	**t**	gaggggctcc	*37*	**29**	>	5	10^{-6}	A		↑	
	rs746670307	tcttcaagct	*10 bp*	**–**	cccagagatt	*38*	**44**	<	3	10^{-3}	B	hyperalgesia and increased scratching post-injury behavior	↓	Naono-Nakayama et al., 2010
TACR1, 162323	rs199873917	tttatattct	*g*	**a*)**	agcgccagtt	*3*	**2**	>	2	0.05	D	higher risks of hyperalgesia	↓	Borbely et al., 2013
TACR2, 162321	rs971537431	tgtctatcac	*g*	**a**	tggaaggatg	*17*	**13**	>	3	10^{-3}	B	increased depression-like behavior	↓	Bardelli et al., 2013

Supplementary Material

References

Andero, R., Brothers, S.P., Jovanovic, T., Chen, Y.T., Salah-Uddin, H., Cameron, M. et al. (2013) Amygdala-dependent fear is regulated by Oprl1 in mice and humans with PTSD. *Sci Transl Med*. **5**: 188ra73. doi: 10.1126/scitranslmed.3005656

Aoki, M., Watanabe, Y., Yoshimoto, K., Tsujimura, A., Yamamoto, T., Kanamura, N., and Tanaka, M. (2016) Involvement of serotonin 2C receptor RNA editing in accumbal neuropeptide Y expression and behavioural despair. *Eur J Neurosci*. **43**, 1219-1228. doi: 10.1111/ejn.13233

Arcourt, A., Gorham, L., Dhandapani, R., Prato, V., Taberner, F.J., Wende, H. et al. (2017) Touch receptor-derived sensory information alleviates acute pain signaling and fine-tunes nociceptive reflex coordination. *Neuron*. **93**, 179-193. doi: 10.1016/j.neuron.2016.11.027

Bardelli, C., Amoruso, A., Manzetti, E., Fresu, L.G., Valsesia, R., Zeppegno, P., and Brunelleschi, S. (2013) Recurrent major depressive disorder: Imbalance of neurokinin (NK)-1 and NK-2 receptor expression in monocytes. *Pharmacol Res*. **68**, 24-30. doi: 10.1016/j.phrs.2012.10.022

Bilkei-Gorzo, A., Racz, I., Michel, K., and Zimmer, A. (2002) Diminished anxiety- and depression-related behaviors in mice with selective deletion of the Tac1 gene. *J Neurosci*. **22**, 10046-10052. doi: 10.1523/jneurosci.22-22-10046.2002

Borbely, E., Hajna, Z., Sandor, K., Kereskai, L., Toth, I., Pinter, E. et al. (2013) Role of tachykinin 1 and 4 gene-derived neuropeptides and the neurokinin 1 receptor in adjuvant-induced chronic arthritis of the mouse. *PLoS One*. **8**: e61684. doi: 10.1371/journal.pone.0061684

Borbely, E., Hajna, Z., Nabi, L., Scheich, B., Tekus, V., Laszlo, K. et al. (2017) Hemokinin-1 mediates anxiolytic and anti-depressant-like actions in mice. *Brain Behav Immun*. **59**, 219-232. doi: 10.1016/j.bbi.2016.09.004

Caldwell, H.K., Aulino, E.A., Freeman, A.R., Miller, T.V., and Witchey, S.K. (2017) Oxytocin and behavior: Lessons from knockout mice. *Dev Neurobiol*. **77**, 190-201. doi: 10.1002/dneu.22431

Grissom, N.M., Herdt, C.T., Desilets, J., Lidsky-Everson, J., and Reyes, T.M. (2015) Dissociable deficits of executive function caused by gestational adversity are linked to specific transcriptional changes in the prefrontal cortex. *Neuropsychopharmacology*. **40**, 1353-1363. doi: 10.1038/npp.2014.313

Hendriksen, H., Bink, D.I., Daniels, E.G., Pandit, R., Piriou, C., Slieker, R. et al. (2012) Re-exposure and environmental enrichment reveal NPY-Y1 as a possible target for post-traumatic stress disorder. *Neuropharmacology*. **63**, 733-742. doi: 10.1016/j.neuropharm.2012.05.028

Huang, G.J., Edwards, A., Tsai, C.Y., Lee, Y.S., Peng, L., Era, T. et al. (2014) Ectopic cerebellar cell migration causes maldevelopment of Purkinje cells and abnormal motor behaviour in Cxcr4 null mice. *PLoS One*. **9**: e86471. doi: 10.1371/journal.pone.0086471

Kawashima, N., Fugate, J., and Kusnecov, A.W. (2002) Immunological challenge modulates brain orphanin FQ/nociceptin and nociceptive behavior. *Brain Res*. **949**, 71-78. doi: 10.1016/S0006-8993(02)02966-9

Landrum, M.J., Lee, J.M., Riley, G.R., Jang, W., Rubinstein, W.S., Church, D.M., et al. (2014). ClinVar: public archive of relationships among sequence variation and human phenotype. *Nucleic Acids Res*. 42, D980-D985. doi:10.1093/nar/gkt1113

Longo, A., Mele, P., Bertocchi, I., Oberto, A., Bachmann, A., Bartolomucci, A. et al. (2014) Conditional inactivation of neuropeptide Y Y1 receptors unravels the role of Y1 and Y5 receptors coexpressing neurons in anxiety. *Biol Psychiatry*. **76**, 840-849. doi: 10.1016/j.biopsych.2014.01.009

Supplementary Material

McLaughlin, P.J., Keiper, C.L., Verderame, M.F., and Zagon, I.S. (2012) Targeted overexpression of OGFr in epithelium of transgenic mice suppresses cell proliferation and impairs full-thickness wound closure. *Am J Physiol Regul Integr Comp Physiol*. **302**, R1084-R1090. doi: 10.1152/ajpregu.00670.2011

Melo, I., Drews, E., Zimmer, A., and Bilkei-Gorzo, A. (2014) Enkephalin knockout male mice are resistant to chronic mild stress. *Genes Brain Behav*. **13**, 550-558. doi: 10.1111/gbb.12139

Naik, R.R., and de Jong, T.R. (2017) Transient and persistent behavioral and molecular changes in primiparous female Wistar rats. *Eur J Neurosci*. **45**, 797-804. doi: 10.1111/ejn.13411

Naono-Nakayama, R., Sunakawa, N., Ikeda, T., Matsushima, O., and Nishimori, T. (2010) Subcutaneous injection of endokinin C/D attenuates carrageenan-induced inflammation. *Peptides*. **31**, 1767-1771. doi: 10.1016/j.peptides.2010.05.019

Painsipp, E., Herzog, H., and Holzer, P. (2010) Evidence from knockout mice that neuropeptide-Y Y2 and Y4 receptor signalling prevents long-term depression-like behaviour caused by immune challenge. *J Psychopharmacol*. **24**, 1551-1560. doi: 10.1177/0269881109348171

Picard, P., Regoli, D., and Couture, R. (1994) Cardiovascular and behavioural effects of centrally administered tachykinins in the rat: characterization of receptors with selective antagonists. *Br J Pharmacol*. **112**, 240-249.

Ragu Varman, D., and Rajan, K.E. (2015) Environmental enrichment reduces anxiety by differentially activating serotonergic and neuropeptide Y (NPY)-ergic system in indian field mouse (mus booduga): an animal model of post-traumatic stress disorder. *PLoS One*. **10**: e0127945. doi: 10.1371/journal.pone.0127945

Riters, L.V., Cordes, M.A., and Stevenson, S.A. (2017) Prodynorphin and kappa opioid receptor mRNA expression in the brain relates to social status and behavior in male European starlings. *Behav Brain Res*. **320**, 37-47. doi: 10.1016/j.bbr.2016.11.050

Ruegsegger, G.N., Toedebusch, R.G., Will, M.J., and Booth, F.W. (2015) Mu opioid receptor modulation in the nucleus accumbens lowers voluntary wheel running in rats bred for high running motivation. *Neuropharmacology*. **97**, 171-181. doi: 10.1016/j.neuropharm.2015.05.022

Ruegsegger, G.N., Brown, J.D., Kovarik, M.C., Miller, D.K., and Booth, F.W. (2016) Mu-opioid receptor inhibition decreases voluntary wheel running in a dopamine-dependent manner in rats bred for high voluntary running. *Neuroscience*. **339**, 525-537. doi: 10.1016/j.neuroscience.2016.10.020

Sajdyk, T.J., Schober, D.A., and Gehlert, D.R. (2002) Neuropeptide Y receptor subtypes in the basolateral nucleus of the amygdala modulate anxiogenic responses in rats. *Neuropharmacology*. **43**, 1165-1172. doi: 10.1016/S0028-3908(02)00234-4

Saltzman, W., Boettcher, C.A., Post, J.L., and Abbott, D.H. (2011) Inhibition of maternal behaviour by central infusion of corticotrophin-releasing hormone in marmoset monkeys. *J Neuroendocrinol*. **23**, 1139-1148. doi: 10.1111/j.1365-2826.2011.02153.x

Sherry, S.T., Ward, M.H., Kholodov, M., Baker, J., Phan, L., Smigielski, E.M., et al. (2001). dbSNP: the NCBI database of genetic variation. *Nucleic Acids Res*. **29**, 308–311. doi:10.1093/nar/29.1.308

Smeets, C.J., Jezierska, J., Watanabe, H., Duarri, A., Fokkens, M.R., Meijer, M. et al. (2015) Elevated mutant dynorphin A causes Purkinje cell loss and motor dysfunction in spinocerebellar ataxia type 23. *Brain*. **138**, 2537-2552. doi: 10.1093/brain/awv195

Supplementary Material

Szklarczyk, K., Korostynski, M., Golda, S., Solecki, W., and Przewlocki, R. (2012) Genotype-dependent consequences of traumatic stress in four inbred mouse strains. *Genes Brain Behav*. **11**, 977-985. doi: 10.1111/j.1601-183X.2012.00850.x

Szklarczyk, K., Korostynski, M., Cieslak, P.E., Wawrzczak-Bargiela, A., and Przewlocki, R. (2015) Opioid-dependent regulation of high and low fear responses in two inbred mouse strains. *Behav Brain Res*. **292**, 95-101. doi: 10.1016/j.bbr.2015.06.001

Vaanholt, L.M., Turek, F.W., and Meerlo, P. (2003) Beta-endorphin modulates the acute response to a social conflict in male mice but does not play a role in stress-induced changes in sleep. *Brain Res*. **978**, 169-176. doi: 10.1016/S0006-8993(03)02805-1

Wang, S.S., Kamphuis, W., Huitinga, I., Zhou, J.N., and Swaab, D.F. (2008) Gene expression analysis in the human hypothalamus in depression by laser microdissection and real-time PCR: the presence of multiple receptor imbalances. *Mol Psychiatry*. **13**, 786-799. doi: 10.1038/mp.2008.38

Wiren, A., Wright, D., and Jensen P. (2013) Domestication-related variation in social preferences in chickens is affected by genotype on a growth QTL. *Genes Brain Behav*. **12**, 330-337. doi: 10.1111/gbb.12017

Yang, F., Sun, W., Yang, Y., Wang, Y., Li, C.L., Fu, H. et al. (2015) SDF1-CXCR4 signaling contributes to persistent pain and hypersensitivity via regulating excitability of primary nociceptive neurons: involvement of ERK-dependent Nav1.8 up-regulation. *J Neuroinflammation*. **12**: 219. doi: 10.1186/s12974-015-0441-2
