# Supplementary material for: Natural Selection Equally Supports the Human Tendencies in Subordination and Domination: A Genome-Wide Study With in silico Confirmation and in vivo Validation in Mice
Source: Front Genet. 2019 Feb 20;10:73. doi: 10.3389/fgene.2019.00073 (PMC6404730; doi:10.3389/fgene.2019.00073)
Supplement: Supplementary file 2 [file Data_Sheet_2.PDF]

frontiers

Supplementary Material

Natural selection equally supports the human tendencies in subordination and domination: a genome-wide study with *in silico* confirmation and *in vivo* validation in mice

Irina Chadaeva, Petr Ponomarenko, Dmitry Rasskazov, Ekaterina Sharypova, Elena Kashina, Maxim Kleshchev, Mikhail Ponomarenko*, Vladimir Naumenko, Ludmila Savinkova, Nikolay Kolchanov, Ludmila Osadchuk, Alexandr Osadchuk

***Correspondence:** Mikhail Ponomarenko (pon@bionet.nsc.ru)

Table S2. Candidate SNP markers predicted in this work near TBP-binding sites in the promoter of the human genes encoding proteins, which relate to non-neuropeptidergic-system (e.g., receptors, enzymes, transporters)

Gene, OMIM	dbSNP (Sherry et al., 2001)	5′ flank	*wt*	**mut**	3′ flank	K_D, nM, prediction: *wt*	**mut**	Δ	Z	α	ρ	**Known physiological or** candidate SNP$ markers	Ss	**ClinVar** or Reference
ADORA1, 102775	rs75340567	cccttgcttc	*c*	**t**	ctgagcctgc	*72*	**56**	>	5	10^{-3}	B	reduced depression-like behavior	↑	Serchov et al., 2015
	rs527838054	gaagtccctt	*g*	**a**	cttccctgag	*72*	**22**	>	21	10^{-6}	A		↑	
	rs963541847	cggaggatga	*g*	**a**	gagggagggg	*50*	**31**	>	10	10^{-6}	A		↑	
	rs200802358	ggaggagacg	*g*	**a**	aggatgagga	*50*	**39**	>	5	10^{-6}	A		↑	
	rs201356416	gccctgtgac	*c*	**t**	ctccggaagt	*72*	**47**	>	7	10^{-6}	A		↑	
	rs563688288	tcctccacat	*a*	**g**	tggggaaatg	*7*	**19**	<	14	10^{-6}	A	hypersensitivity to pain	↓	Wu et al., 2005
	rs6664108	ttcctccaca	*t*	**c**	atggggaaat	*7*	**20**	<	16	10^{-6}	A		↓	
	rs577420345	ttcttcctcc	*c*	**t**	catatgggga	*7*	**10**	<	5	10^{-6}	A		↓	
	rs761937437	tgccgtacca	*t*	**a, c**	gtgattgctt	*15*	**22**	<	6	10^{-6}	A		↓	
	rs911566207	ggtgccgccg	−	**6 bp**	ccgccccccgc	*50*	**62**	<	4	10^{-3}	B		↓	
ADORA2A, 102776	rs200174085	cttcacgtga	*c*	**t**	gtgacgtggc	*45*	**32**	>	7	10^{-6}	A	improved survival in post-traumatic endotoxemia and sepsis	↑	Sullivan et al., 2004
	rs539804305	caatccttca	*c*	**t**	gtgacgtgac	*45*	**20**	>	15	10^{-6}	A		↑	
	rs200817204	ggtgccaatc	*c*	**a**	ttcacgtgac	*45*	**25**	>	13	10^{-6}	A		↑	
	rs199593618	tgcccgccat	*c*	**g**	tgagggaggt	*45*	**32**	>	7	10^{-6}	A		↑	
	rs932430866	cctgttgccc	*g*	**a**	ccatctgagg	*45*	**34**	>	6	10^{-6}	A		↑	
	rs143744701	ccgtccgtca	*gtcc*	−	gtccgtccgt	*100*	**47**	>	13	10^{-6}	A		↑	
	rs1039128064	gggcggggag	*16 bp*	−	cgtccgtccg	*100*	**47**	>	13	10^{-6}	A		↑	
	rs904568725	ggcggggagc	*12 bp*	−	gtccgtccgt	*100*	**47**	>	13	10^{-6}	A		↑	
	rs1036721475	gcgctccaag	*g*	**a**	ggcggggaag	*100*	**78**	>	5	10^{-6}	A		↑	
	rs932135939	tgccagggac	*c*	**t**	aaactcccgt	*22*	**12**	>	10	10^{-6}	A		↑	
	rs898102962	tcacgtgacg	*t*	−	gacgtggctc	*45*	**51**	<	3	10^{-2}	C	predisposition to fearfulness, helplessness, and fatigue	↓	Azzinnari et al., 2014
	rs201534466	aatccttcac	g	**c**	tgacgtgacg	*45*	**54**	<	4	10^{-3}	B		↓	
	rs561125568	gccgcaggta	g	**c**	cgggcgggga	*45*	**55**	<	2	0.05	D		↓	
ADORA2B (600446)	rs145053625	tgggacacag	*c*	**g**	ctccgagctc	*56*	**48**	>	3	0.05	D	increased voluntary physical inactivity behavior	↓	Budiono et al., 2016

Notes: hereinafter, Alleles: wt, ancestral; mut, minor; "−", deletion; K_D, dissociation constant of TBP–DNA complex; α = 1 – p, significance (where p value is given in Figure 1); **Δ, changes**: excess (>) and deficit (<); **Ss, Social status**: dominance (↑) and subordination (↓); ρ, heuristic rank of candidate SNP markers varying in alphabetical order from the "best" (A) to the "worst" (E). [*)]This SNP also includes other neutral alleles. **ClinVar**, the database of clinical annotations of SNPs (Landrum et al., 2014); Reference, the *italicized references* found by our manual keyword search in the PubMed database (Figure S1, Supplementary file 1) the contents of which are *italicized* in the third rightmost column. **Genes**: *ADOR*s, adenosine receptors; *ADR*s, adrenoceptors; *CHRM*s, muscarinic cholinergic receptors; *CHRN*s, nicotinic cholinergic receptors; *CNR1*, cannabinoid receptor 1 (central); *COMT*, catechol-O-methyltransferase; *DRD*s, dopamine D receptors; *GABARAP*s, GABA type A receptor–associated proteins; *GABBR*s, γ-aminobutyric acid type B receptor subunits; *GABR*s, γ-aminobutyric acid type A receptor subunits; *GRP*s, G protein–coupled receptors; *GRIA*s, glutamate ionotropic receptor AMPA type subunits, *GRIN*s, glutamate ionotropic receptor NMDA type subunits; *GRM*s, glutamate metabotropic receptors; *HTR*s, 5-hydroxytryptamine (serotonin) receptors; *SLC6A3*, dopamine transporter (DAT); *SLC6A4*, Na^+/Cl^--dependent serotonin transporter (SERT); *TH*, tyrosine hydroxylase; *TPH2*, tryptophan hydroxylase 2. **Deletion / insertion**, ADORA1: 6 bp = ccgccc; ADORA2A: 12 bp = gtccgtccgtca, 16 bp = cgtccgtccgtcagtc; CHRM2: 7 bp = aaccacg; CHRNA4: 5 bp = ttctc, 14 bp = cctgctccccctcc, 11 bp = cctgctccccc, 18 bp = agctccccctcctcccca; CHRNA5: 22 bp[a)] = cagagggaaataggggcggggc, 22 bp[b)] = gggcggggccagagggaaatag; CHRNB4: 10 bp = ggcggggctc; COMT: 12 bp[a)] = gcgccccgcgcc, 12 bp[b)] = gctgcgccccgc, 19 bp = tcaccaggggcgaggctca; DRD2: 10 bp = ccgccccgcc; 37 bp = gctccctcctgcccgcccgccccgcgcccggccccgc; GABRA2: 11 bp = tctctctctct; GABRA5: 12 bp = gcgggcgcgagt; GABRB3: 6 pb = tagggg, 25 bp = tcaggcgggaaagcctgggggtggg; GABRG3: 11 bp = gtgtgcgtcca; GRIA2: 8 bp = tgtgtgta; GRIN2C: 12 bp = cgccgccgccgc, 9 bp = cgccgccgc; GRINA: 25 bp = cccgccccgtcacaaggccccgctg, 29 bp = ccctacccccaagggggctacccacaggg; GRM2: 25 bp = gccgcgattgaaagcggcggggcga; GRM3: 6 bp = ccgccg; SLC6A3: 5 bp = taaga.

Supplementary Material

Table S2. Continued

Gene, *OMIM*	dbSNP (Sherry et al., 2001)	5′ flank	wt	**mut**	3′ flank	K_D, nM, prediction						**Known physiological or** candidate SNP$^{\$}$ markers	Ss	**ClinVar** or Reference
						wt	**mut**	Δ	Z	α	ρ			
ADORA3, 600445	rs764419607	agcttctgtg	g	**a, t**	cagttggtag	*46*	**32**	>	7	10^{-6}	A	reduced sensitivity to chronic pain	↑	Little et al., 2015
	rs778722569	aatcatgtgg	c	**t**	cctgagggac	*31*	**26**	>	3	10^{-2}	C		↑	
	rs746708794	cctaacagca	g	**t*)**	caatcatgtg	*31*	**16**	>	8	10^{-6}	A		↑	
	rs770545601	cccctaacag	c	**t**	accaatcatg	*31*	**10**	>	15	10^{-6}	A		↑	
	rs776212806	tcccctaaca	g	**a, t**	caccaatcat	*31*	**25**	>	3	10^{-3}	A		↑	
	rs763336527	cgccttctcc	c	**t**	ctaacagcac	*31*	**17**	>	9	10^{-6}	A		↑	
	rs760053434	ttggaccaat	g	**a**	gaagggtctc	*27*	**16**	>	8	10^{-6}	A		↑	
	rs41282524	accagttgga	c	**t**	caatggaagg	*27*	**19**	>	6	10^{-6}	A		↑	
	rs1056080027	gttctgagct	c	**g**	tgtacttcct	*14*	**11**	>	3	10^{-3}	B		↑	
	rs762899000	ctgtggcagt	t	**c**	ggtagagacc	*27*	**35**	<	4	10^{-3}	B	platelet deficit increases bleeding without coagulation in trauma	↓	Budiono et al., 2016
	rs563795796	gagctctgta	c	**t**	ttcctcttgg	*14*	**19**	<	3	10^{-3}	B		↓	
ADRA1A, 104221	rs866984610	tggtttgagg	*g*	**a, t**	agagactggc	*29*	**19**	>	8	10^{-6}	A	increased antidepressant-like behavior	↑	Doze et al., 2009
	rs527441244	gccggacctc	*g*	**a*)**	cccggccccg	*157*	**139**	>	2	0.05	D		↑	
	rs113731073	acagccggac	*c*	**t**	tcgcccggcc	*157*	**118**	>	5	10^{-6}	A		↑	
	rs764066485	gacagccgga	*c*	**a**	ctcgcccggc	*157*	**131**	>	3	10^{-3}	B		↑	
	rs199709396	ctggacagcc	*g*	**a**	gacctcgccc	*157*	**116**	>	5	10^{-6}	A		↑	
	rs761990725	cctggacagc	*c*	**a, t*)**	ggacctcgcc	*157*	**111**	>	6	10^{-6}	A		↑	
	rs879876802	ggaggtggcc	*c*	**t**	tggacagccg	*157*	**103**	>	7	10^{-6}	A		↑	
	rs201629138	ccgggaggtg	*g*	**t**	ccctggacag	*157*	**87**	>	9	10^{-6}	A		↑	
	rs192591538:**a**	ttggtttgag	*g*	**a**	gagagactgg	*29*	**19**	>	8	10^{-6}	A		↑	
	rs192591538:**c**	ttggtttgag	*g*	**c**	gagagactgg	*29*	**34**	<	3	10^{-3}	B	weakened manifestation of post-traumatic stress disorders	↑	Stojkov et al., 2013
	rs915074831	tcttttggtt	*t*	**c**	gagggagaga	*29*	**32**	<	2	0.05	D		↑	
ADRA1D, 104219	rs908080015	gccgcgctcc	*g*	**a**	cgtcacagga	*56*	**45**	>	4	10^{-3}	B	reduced muscle cell loss in trauma, disease, and ageing	↑	Girven et al., 2016
ADRA2C, 104250	rs1054405296	ggggccgcgc	*g*	**a**	aggaccccgg	*328*	**153**	>	14	10^{-6}	A	reduced nociceptive behavior	↑	Roh et al., 2010
	rs556140155	tctgcactta	*c*	**a**	acgctcggca	62	**33**	>	12	10^{-6}	A		↑	
ADRB2, 109690	rs764196915	ccgtacgtca	*c*	**t**	ggcgagggca	*21*	**26**	<	3	10^{-2}	C	hypersensitivity to neuropathic pain	↓	Yalcin et al., 2009
	rs896733341	gttcccgtac	*g*	**c**	tcacggcgag	*21*	**40**	<	9	10^{-6}	A		↓	
ADRB3, 109691	rs561595814	gaatcctttg	*c*	**t**	ttgactccag	*47*	**26**	>	11	10^{-6}	A	increased antidepressant-like behavior	↑	Claustre et al., 2008
CHRM1, 118510	rs532008081	tcttgtactg	*c*	**t**	gtcatagggg	*6*	**5**	>	4	10^{-3}	B	higher autoimmunity risks for chronic fatigue syndrome	↓	Tanaka, S. et al., 2003
	rs372160563	ctgggggctc	*t*	**c**	aaggttgggt	*27*	**69**	<	15	10^{-6}	A	higher risks for behavioral deficit because of cerebral ischemic states	↓	Kumaran et al., 2008
CHRM2, 118493	rs908253872	catactgaaa	*g*	**t**	aaaattgtaa	*10*	**7**	>	5	10^{-6}	A	increased antidepressant-like behavior	↑	Gibbons et al., 2016
	rs1043831801	ctccagtcta	*7 bp*	**–**	aacaggtcac	*17*	**12**	>	5	10^{-6}	A		↑	
	rs945458080	cagcgtcaat	*t*	**g**	taactccagt	*9*	**10**	<	2	0.05	D	worse spatio-temporal learning and memory	↓	Chauhan et al., 2016
CHRM3, 118494	rs762881034	ggattagact	*c*	**t**	aaagtaagct	*8*	**5**	>	8	10^{-6}	A	worse oligodendrocyte repair in post-injury brain and spinal cord	↓	Abiraman et al., 2015
CHRM4, 118495	rs778486025	gtagttcatg	*c*	**t**	ttttctctcc	*33*	**25**	>	5	10^{-6}	A	reduced long-term synaptic depression-like behavior	↑	Thomson et al., 2017
	rs760241570	tgtagttcat	*g*	**a**	cttttctctc	*33*	**9**	>	21	10^{-6}	A		↑	
	rs200436334	tagttcatgc	*c*	**t**	tttctctccc	*33*	**43**	<	5	10^{-6}	A	reduced pain sensitivity	↑	Mulugeta et al., 2003
CHRM5, 118496	rs993148565	ttgttacata	*g*	**t**	gagatgacag	*4*	**3**	>	3	10^{-2}	C	reduced fright behavior	↑	Garzon, Pickel, 2013
	rs142387857	agcaagagtg	*c*	**t**	aagaacatcc	*17*	**9**	>	10	10^{-6}	A		↑	
	rs746670103	tgaaaagttc	*g*	**a**	tatggagaaa	*17*	**10**	>	8	10^{-6}	A		↑	
	rs990622674	ggactgaaaa	*g*	**c**	ttcgtatgga	*17*	**14**	>	3	10^{-2}	C		↑	
	rs572918743	tgaatcttta	*a*	**g**	taagttctca	*5*	**7**	<	5	10^{-3}	B	increased chronic stress-induced BDNF-deficiency with aggressive behavior	↑	Sakata, Overacre, 2017

Table S2. Continued

Gene, OMIM	dbSNP (Sherry et al., 2001)	5′ flank	wt	**mut**	3′ flank	K_D, nM, prediction wt	**mut**	Δ	Z	α	ρ	**Known physiological or** candidate SNP[$] markers	Ss	**ClinVar** or Reference
CHRNA1, 100690	rs557281141	ctccagcaga	c	**a**	aagcacctcc	*37*	**21**	>	11	10^{-6}	A	higher risks for post-traumatic denervation	↓	Manzano et al., 2011
	rs562123768	tagctctagt	*g*	**a**	agccgactcg	*21*	**9**	>	12	10^{-6}	A		↓	
	rs561014664	acaggtggtg	t	**c**	aaaacaatag	*7*	**21**	<	19	10^{-6}	A	higher risks for axonal defects during neuromuscular hyperactivity	↓	Lefebvre et al., 2004
CHRNA2, 118502	rs56278204	gtgtgtgtgt	*g*	**a**	tgtatgtgtg	*18*	**12**	>	8	10^{-6}	A	increased epilepsy-like ictal fear behavior	↓	Aridon et al., 2006
	rs2435312	gtgtgtgtgt	*g*	**a**	tgtgtatgtg	*18*	**10**	>	12	10^{-6}	A		↓	
	rs906138524	tttgaccatg	*a*	**t**	aatgaagtga	*15*	**8**	>	10	10^{-6}	A		↓	
	rs371092293	gagggaggat	*c*	**g**	tgagaaagcc	*48*	**32**	>	8	10^{-6}	A		↓	
	rs958703537	agggaaggaa	c	**t**	gagtggggca	*48*	**31**	>	8	10^{-6}	A		↓	
	rs751097709	tttaggggct	*g*	**a**	aagggaagga	*48*	**24**	>	12	10^{-6}	A		↓	
	rs879866890	tgtccccacc	*g*	**a**	gacataggct	*19*	**13**	>	12	10^{-6}	A		↓	
CHRNA3, 118503	rs67112589	ccccggaaac	c	**t**	tgggacagaa	*27*	**31**	<	2	0.05	D	increased depression-like behavior	↓	Han et al., 2017
	rs912450652	atttaatagc	*a*	**g**	tgagatacac	*3*	**4**	<	2	0.05	D		↓	
CHRNA4, 118504	rs188041521	cccctcccct	*g*	**t**	ctccccctcc	*250*	**96**	>	18	10^{-6}	A	high risks of arrhythmias leading to congestive heart failure	↓	Andersson et al., 2006
	rs189766137	tccccccctcc	*c*	**a, t*)**	ctgctccccc	*250*	**107**	>	16	10^{-6}	A		↓	
	rs776226454	catcttttta	−	**5 bp**	ttcccccatt	*250*	**56**	>	28	10^{-6}	A		↓	
	rs745328736	ctccccctcc	*14 bp*	−	tccccccctcc	*250*	**27**	>	42	10^{-6}	A		↓	
	rs374693733	gctccccctc	−	**tt**	ctccccccctc	*250*	**64**	>	25	10^{-6}	A		↓	
	rs771923793	cctgctcccc	c	**g**	tcctcccccc	*250*	**203**	>	4	10^{-3}	B		↓	
	rs369122502	ctccccctcc	*11 bp*	−	tcctcccccc	*250*	**38**	>	7	10^{-6}	A		↓	
	rs372065917	ccctgctccc	−	**t**	cctcctcccc	*250*	**211**	>	3	10^{-2}	C		↓	
	rs375134688	ctccccctcc	−	**t**	cctgctcccc	*250*	**133**	>	12	10^{-6}	A		↓	
	rs767866571	ttccctcccc	−	**18 bp**	ctcccctgct	*250*	**182**	>	7	10^{-6}	A		↓	
	rs762020800	gttccctccc	*c*	**a**	ctcccctgct	*250*	**218**	>	2	0.05	D		↓	
	rs773622761	tctccgttcc	*c*	**t**	tccccctccc	*250*	**180**	>	6	10^{-6}	A		↓	
CHRNA5, 118505	rs543210727	tcaagactac	*g*	**a**	aaagatgggt	*10*	**6**	>	8	10^{-6}	A	hypersensitivity to mechanical pain	↓	Vincler, Eisenach, 2005
	rs79835149	ttcaagacta	*c*	**t*)**	gaaagatggg	*10*	**6**	>	8	10^{-6}	A		↓	
	rs201040941	gatttatttc	*c*	**t**	agactacgaa	*10*	**5**	>	11	10^{-6}	A		↓	
	rs74865777	ggtatttacc	*a*	**g**	tgatttttgt	*9*	**7**	>	2	0.05	D		↓	
	rs780560672	tggtatttac	*c*	**t**	atgatttttg	*9*	**5**	>	11	10^{-6}	A		↓	
	rs67624739	tgggcggggc	*22 bp*[a)]	−	taggcgccgg	*22*	**59**	<	18	10^{-6}	A	reduced mechanical pain sensitivity	↑	
	rs142774214	ggcgaggatt	*22 bp*[b)]	−	gggcggggct	*22*	**59**	<	18	10^{-6}	A		↑	
	rs201230605	agcttccaca	*t*	**c, g**	gcgtcccgag	*37*	**52**	<	6	10^{-6}	A		↑	
	rs199898580	gagcttccac	*a*	**g, t**	tgcgtcccga	*37*	**52**	<	9	10^{-6}	A		↑	
CHRNA6, 606888	rs114140075	catgagcttt	*a*	**g**	cctgtagttg	*13*	**19**	<	5	10^{-6}	A	maternal aggressive behavior during feeding of offspring	↑	Mann, 2014
	rs1023148339	agatgtgctt	*t*	**c**	gttaaacaaa	*7*	**10**	<	6	10^{-6}	A		↑	
CHRNA7, 118511	rs912076951	ggcgaggtgc	*c*	**t*)**	tctgtggccg	*74*	**46**	>	9	10^{-6}	A	behavioral and cognitive benefits	↑	Uteshev, 2012
	rs535530714	gcggcgaggt	*g*	**a*)**	cctctgtggc	*74*	**29**	>	16	10^{-6}	A		↑	
	rs906600313	aggcgcgcgg	−	**ggg**	gggcgggcgg	*41*	**70**	<	9	10^{-6}	A	increased antidepressant-like behavior	↑	Rabenstein et al., 2006
	rs986291354	cggcgaggtg	*c*	**g**	ctctgtggcc	*74*	**87**	<	3	10^{-2}	C		↑	
CHRNA9, 605116	rs778582775	ctcaaattac	*c*	**t**	ggaggggagg	*19*	**13**	>	5	10^{-3}	B	mechanical hyperalgesia	↓	Mohammadi, Christie, 2015
	rs558335465	gtctcaaatt	*a*	**t**	ccggagggga	*19*	**42**	<	14	10^{-6}	A	reduced mechanical algesia	↑	
CHRNA10, 606372	rs753922071	cttctgactt	*c*	**t**	taggtactgt	*8*	**7**	>	2	0.05	D	hypersensitive to neuropathic pain	↓	Vincler et al., 2006
	rs780194406	ttctgacttc	*t*	**c**	aggtactgtc	*8*	**18**	<	14	10^{-6}	A	low sensitivity to neuropathic pain	↑	
CHRNB1, 100710	rs567974262	ggtgcacatt	*c*	**g**	ccgggctcct	*49*	**40**	>	4	10^{-3}	B	increased efficiency of muscle functioning	↑	Burniston et al., 2013
	rs1054460489	gggcctggga	*c*	**t**	gagaccaggc	*68*	**44**	>	8	10^{-6}	A		↑	
	rs757646406	cttttctctg	*g*	**t**	ctatgatagc	*17*	**10**	>	10	10^{-6}	A		↑	
	rs751068058	ctggctatga	*t*	**c**	agctccgtgc	*17*	**21**	<	3	10^{-3}	B	increased anesthetic-like behavior	↑	Spitzmaul et al., 2009
	rs777431654	ttctctggct	−	**atg**	atagctccgt	*17*	**35**	<	14	10^{-6}	A		↑	
	rs779475593	tctctggcta	*t*	**c**	gatagctccg	*17*	**27**	<	8	10^{-6}	A		↑	
CHRNB3, (118508)	rs56279199	agacttacac	*g*	**t**	atctgacagc	*9*	**6**	>	6	10^{-6}	A	reduced stress-related anhedonia-like depressive behavior	↑	Han et al., 2017

Supplementary Material

Table S2. Continued

Gene, OMIM	dbSNP (Sherry et al., 2001)	5′ flank	wt	**mut**	3′ flank	K_D, nM, prediction wt	**mut**	Δ	Z	α	ρ	**Known physiological or** candidate SNP[$] markers	Ss	**ClinVar** or Reference
CHRNB4, 118509	rs941163904	ggcggggctc	*10 bp*	**–**	tccgcggggt	*33*	**14**	>	12	10^{-6}	A	reduced impulsive behavior and working memory	↓	Vinals et al., 2012
	rs532586426	gcccagtaca	*t*	**a**	ccctgagtgg	*33*	**28**	>	3	0.05	D		↓	
	rs879768188	gcggggtcct	*a*	**g**	gtgagcgccg	*33*	**49**	<	6	10^{-6}	A	increased stress-related anhedonia-like depressive behavior	↓	Han et al., 2017
	rs1017811487	cgcggggtcc	*t*	**g**	agtgagcgcc	*33*	**49**	<	6	10^{-6}	A		↓	
	rs1018893356	ctccgcgggg	*t*	**g**	cctagtgagc	*33*	**49**	<	5	10^{-6}	A		↓	
CHRNE, 100725	rs2302315	ctccccctca	*c*	**t**	acaggcaccc	*36*	**22**	>	9	10^{-6}	A	predisposition to muscle weakness	↓	Maclennan et al., 2008
	rs765722526	ccagagctca	*g*	**a**	aataaccctg	*36*	**19**	>	12	10^{-6}	A		↓	
	rs865902930	catgcccccct	*c*	**a**	caagcctgcc	*36*	**22**	>	9	10^{-6}	A		↓	
CHRNG, 100730	rs992850125	gttatatgac	*a*	**g**	cccagagccc	*13*	**36**	<	15	10^{-6}	A	predisposition to muscle weakness	↓	Hoffmann et al., 2006
CNR1, 114610	rs183543261	catgacagta	*c*	**t**	atttgccaca	*5*	**3**	>	5	10^{-6}	A	adolescence-like behavior with high risk/novelty seeking and enhanced impulsivity	↑	Schneider et al., 2015
	rs761017586	cctaatcaaa	*g*	**c[*)]**	actgaggtta	*25*	**21**	>	3	10^{-2}	C		↑	
	rs776807704	ttgagctcag	*c*	**a, t[*)]**	ctaatcaaag	*25*	**16**	>	8	10^{-6}	A		↑	
	rs62417860	ttttgagctc	*a*	**c**	gcctaatcaa	*25*	**22**	>	2	0.05	D		↑	
	rs369660344	gtcactttct	*c*	**t**	agtcattttg	*25*	**15**	>	10	10^{-6}	A		↑	
	rs905978966	gcctccgcct	*c*	**t**	ttcttgtctc	*29*	**26**	>	3	10^{-2}	C		↑	
	rs188453060	gctccatctt	*a*	**g**	cttaccaaat	*7*	**10**	<	6	10^{-6}	A	increased anxiety-like behavior in males	↑	Bowers, Ressler, 2016
COMT, 116790	rs777650793	gtccgccacc	*g*	**a**	gaagcgccct	*187*	**81**	>	15	10^{-6}	A	**cardiovascular disease**	↓	**Landrum et al., 2014**
	rs45593642	ccaccggaag	*c*	**a**	gccctcctaa	*187*	**114**	>	9	10^{-6}	A	*higher risks for cerebrovascular vasospasm* as well as increased depression-like behavior	↓	*He et al., 2011;* as well as Wilhelm et al., 2013
	rs45581136	gccaccggaa	*g*	**a**	cgccctccta	*187*	**160**	>	3	10^{-2}	C		↓	
	rs868447575	cgtccgccac	*c*	**a**	ggaagcgccc	*187*	**74**	>	17	10^{-6}	A		↓	
	rs758503929	cggcctgcgt	*c*	**a**	cgccaccgga	*187*	**37**	>	24	10^{-6}	A		↓	
	rs748298389	gccacggcct	*g*	**t**	cgtccgccac	*187*	**70**	>	18	10^{-6}	A		↓	
	rs562298402	cgcccgccac	*g*	**a**	gcctgcgtcc	*187*	**150**	>	4	10^{-3}	B		↓	
	rs866665461	tcctaatccc	*c*	**a**	gcagcgccac	*27*	**22**	>	3	10^{-3}	B		↓	
	rs766448975	cctacctgct	*12 bp[a)]*	**–**	gcgccccgca	*38*	**27**	>	5	10^{-6}	A		↓	
	rs1008629337	catcctacct	–	*12 bp[b)]*	gctgcgcccc	*38*	**27**	>	5	10^{-6}	A		↓	
	rs761297531	ccccatccta	*c*	**t**	ctgctgcgcc	*38*	**22**	>	8	10^{-6}	A		↓	
	rs565340849	ttgatgtgct	*g*	**t**	attaaagtag	*8*	**5**	>	9	10^{-6}	A		↓	
	rs147942364	ggctcatcac	*c*	**t**	atcgagatca	*37*	**33**	>	2	0.05	D		↓	
	rs745493295	ccgcctgctg	*19 bp*	**–**	tcaccatcga	*37*	**24**	>	8	10^{-6}	A		↓	
	rs72563160	gagcacagag	*c*	**t**	actggcgccc	*58*	**21**	>	16	10^{-6}	A		↓	
	rs753104108	gctgcaggag	*g*	**a**	agcacagagc	*58*	**50**	>	3	10^{-2}	C		↓	
	rs4818	gggcgaggct	*c*	**t[*)]**	atcaccatcg	*37*	**21**	>	10	10^{-6}	A		↓	
	rs188402271	ccactggatc	*a*	**g**	caaatcaaac	*21*	**23**	<	2	0.05	D	increased anxiety-like behavior	↑	Desbonnet et al., 2012
	rs901020754	acggcctgcg	*t*	**c**	ccgccaccgg	*187*	**243**	<	5	10^{-3}	B		↑	
	rs779542396	cgccacggcc	*t*	**c**	gcgtccgcca	*187*	**243**	<	5	10^{-3}	B		↑	
	rs760676485	actgtggcta	*c*	**a**	tcagctgtgc	*37*	**50**	<	6	10^{-6}	A		↑	
	rs773235901	cctactgtgg	*c*	**t**	tactcagctg	*37*	**61**	<	9	10^{-6}	A		↑	
	rs377353154	gcacagagca	*c*	**t[*)]**	tggcgcccct	*58*	**72**	<	4	10^{-3}	B		↑	
DRD1, 126449	rs1016706882	ccagtcattc	*a*	**g**	tataaaggag	*2.4*	**2.1**	>	2	0.05	D	hedonic aggressive behavior	↑	Freund et al., 2016
DRD2, 126450	rs944079328	cagagctgtc	*c*	**t**	agcttcagtg	*69*	**27**	>	16	10^{-6}	A	increased depression-like behavior	↓	Chen et al., 2013
	rs112073587	tttctgtttc	*g*	**t[*)]**	atgactagaa	*13*	**7**	>	10	10^{-6}	A		↓	
	rs778361148	gtccattttt	–	**t**	cctggccaga	*31*	**28**	>	2	0.05	D		↓	
	rs768906844	ctgacacctt	*g*	**t**	tgtccatttt	*31*	**22**	>	7	10^{-6}	A		↓	
	rs750830011	ctctcactga	*c*	**t**	accttgtgtc	*31*	**9**	>	21	10^{-6}	A		↓	
	rs1049904556	ccctctcact	*g*	**a**	acaccttgtg	*31*	**10**	>	20	10^{-6}	A		↓	
	rs201888901	ggccctctca	*c*	**t**	tgacaccttg	*31*	**20**	>	8	10^{-6}	A		↓	
	rs865881986	ccctcctgcc	*c*	**a**	gcccgccccg	*224*	**175**	>	5	10^{-6}	A		↓	
	rs927067383	tccctcctgc	*c*	**a**	cgcccgcccc	*224*	**119**	>	12	10^{-6}	A		↓	
	rs745914515	agcctggcca	*c*	**a**	ccagtggctc	*30*	**20**	>	8	10^{-6}	A		↓	
	rs1018362228	ccgccccgcc	–	**10 bp**	gcggcccgtc	*224*	**1291**	<	31	10^{-6}	A	reduced depression-like behavior	↑	Graham et al., 2015
	rs1016768674	ccgtccccgc	*37 bp*	**–**	cccgccccgc	*224*	**426**	<	11	10^{-6}	A		↑	
	rs998480433	gtcacatggt	*t*	**g**	ggaaatgtgg	*35*	**39**	<	3	10^{-2}	C		↑	

Supplementary Material

Table S2. Continued

Gene, OMIM	dbSNP (Sherry et al., 2001)	5′ flank	wt	**mut**	3′ flank	K_D, nM, prediction wt	**mut**	Δ	Z	α	ρ	**Known physiological or** candidate SNP$ markers	Ss	**ClinVar** or Reference
DRD3, 126451	rs36211802	tatgtctgct	*g*	**a**	tcagtaaatg	*9*	**8**	>	4	10^{-3}	B	**hereditary essential tremor**	↓	**Landrum et al., 2014**
	rs139345353	ctcatgaatg	*g*	**t**	attaatgttt	*9*	**4**	>	4	10^{-3}	B	*resistance to the high-dose DRD3-agonist drug treatment against tremor* as well as reduced motor activity and behavior motivation	↓	*Kosmowska et al., 2016* as well as Ikeda et al., 2013
	rs575294415	agaaaatata	*c*	**t**	atgtactaaa	*3*	**2**	>	4	10^{-3}	B		↓	
	rs1004196461	atatacatgt	*a*	**g**	ctaaaaagca	*3*	**4**	<	4	10^{-3}	B	increased motor activity and behavior motivation	↑	
DRD4, 126452	rs956929710	cggcgttgtc	*c*	**t**	gcggtgctca	*64*	**39**	>	9	10^{-6}	A	increased stress resilience	↑	Azadmarzabadi et al., 2018
	rs987740098	tgcgacccgg	*c*	**t**	gttgtccgcg	*64*	**38**	>	9	10^{-6}	A		↑	
	rs954996920	cttgcgaccc	*g*	**a**	gcgttgtccg	*64*	**56**	>	3	10^{-2}	C		↑	
	rs1031281855	cgacccggcg	*t*	**c**	tgtccgcggt	*64*	**176**	<	19	10^{-6}	A	higher locomotion and faster response to anxiogenic stimuli	↑	Keck et al., 2013
DRD5, 126453	rs531790860	agggaccgcg	*g*	**a**	cagcgcctca	*381*	**285**	>	6	10^{-6}	A	hypersensitivity to conditioned fear stimuli	↓	Inoue et al., 2000
	rs998429725	gagcggccag	*g*	**a**	gaccgcggca	*381*	**133**	>	19	10^{-6}	A		↓	
	rs965580700	gcggggcctg	*g*	**a**	gagcggccag	*381*	**129**	>	20	10^{-6}	A		↓	
	rs563493722	ggacgcgggg	*c*	**a**	ctgggagcgg	*381*	**197**	>	12	10^{-6}	A		↓	
	rs548367019	ggcgggacgc	*g*	**a**	gggcctggga	*381*	**189**	>	12	10^{-6}	A		↓	
	rs1004925094	ggggcgggac	*g*	**t**	cggggcctgg	*381*	**230**	>	9	10^{-6}	A		↓	
	rs529978867	ctcggggggc	*g*	**a**	ggacgcgggg	*381*	**191**	>	12	10^{-6}	A		↓	
	rs886206991	gctcgggggg	*c*	**a**	gggacgcggg	*381*	**331**	>	3	0.05	D		↓	
	rs187411304	cggaggctcg	*g*	**t**	ggggcgggac	*381*	**131**	>	21	10^{-6}	A		↓	
	rs1054946571	gagcgcggag	*g*	**a**	ctcggggggc	*381*	**176**	>	14	10^{-6}	A		↓	
GABARAP, 605125	rs956917720	ctaaccttgt	*a*	**t*)**	attgtccctg	*14*	**10**	>	5	10^{-3}	B	hypersensitivity to pain caused by physical stimuli	↓	Lainez et al., 2010
	rs990041436	agctaacctt	*g*	**a**	taattgtccc	*14*	**4**	>	15	10^{-6}	A		↓	
	rs541886304	ttggtgaata	*g*	**a**	ggaagtggcg	*25*	**13**	>	12	10^{-6}	A		↓	
	rs935098762	gggttggtga	*a*	**c**	tagggaagtg	*25*	**34**	<	5	10^{-6}	A	higher resistance to fear stimuli	↑	Lin et al., 2009
GABARAPL1, (607420)	rs1056201850	gaaacgcagt	*g*	**t**	agacagagcg	*22*	**15**	>	6	10^{-6}	A	improved muscle endurance, especially when fasting	↑	Jamart et al., 2013
	rs926248250	tagccagaaa	*g*	**a**	gaaacgcagt	*22*	**16**	>	6	10^{-6}	A		↑	
	rs559192393	aggttagcca	*g*	**c**	aaaggaaacg	*22*	**16**	>	5	10^{-6}	A		↑	
	rs182142915	cctggcgcat	*g*	**a**	gggggcgggg	*54*	**39**	>	6	10^{-6}	A		↑	
	rs950190593	cagtgagaca	*g*	**a, c**	agcgctcggc	*54*	**47**	>	3	10^{-2}	C		↑	
	rs1056201850	gaaacgcagt	*g*	**t**	agacagagcg	*54*	**19**	>	15	10^{-6}	A		↑	
	rs755246580	tgggcgcctg	*g*	**a, t**	cgcatggggg	*161*	**100**	>	9	10^{-6}	A		↑	
	rs115488982	gggacattgt	*g*	**a**	ctgtgcggtg	*9*	**8**	>	2	0.05	D		↑	
	rs558989021	acgcgccacc	*c*	**t**	agctgttttt	*62*	**48**	>	4	10^{-3}	B		↑	
	rs115488982	cgtcacagcc	*c*	**t**	gacgcgccac	*62*	**55**	>	3	0.05	D		↑	
	rs977542172	ttagccagaa	*a*	**c, t**	ggaaacgcag	*22*	**27**	<	4	10^{-3}	B	reduced muscle endurance, especially when fasting	↓	
	rs967548733	aaggttagcc	*a*	**c, g**	gaaaggaaac	*22*	**27**	<	4	10^{-3}	B		↓	
	rs754237756	tgaaaaaggt	*t*	**c**	agccagaaag	*22*	**32**	<	4	10^{-6}	A		↓	
	rs1045843683	agacagagcg	*c*	**t*)**	tcggccaccc	*54*	**61**	<	2	0.05	D		↓	
	rs915889927	gcagtgagac	*a*	**g**	gagcgctcgg	*54*	**68**	<	5	10^{-3}	B		↓	
	rs936027386	tcttttctcc	*t*	**c**	gaggctggat	*24*	**30**	<	5	10^{-3}	B		↓	
	rs926195946	ctggccctct	*t*	**c**	ttctcctgag	*24*	**42**	<	11	10^{-6}	A		↓	
	rs977594630	cagctgtttt	*t*	**c**	gtgctcccag	*12*	**15**	<	5	10^{-3}	B		↓	
	rs558801238	ttggctgcct	*t*	**c**	taagaacgca	*9*	**15**	<	8	10^{-6}	A		↓	
GABARAPL2, 182139	rs575443820	ggcgtggcgc	*c*	**t**	ctgacaaatg	*21*	**19**	>	2	0.05	D	accelerated neutrophil differentiation, autophagic flux, and wound healing	↑	Brigger et al., 2013
	rs1029645971	ttggcgtggc	*g*	**a**	ccctgacaaa	*21*	**19**	>	2	0.05	D		↑	
	rs762096646	cgcctgccgt	*g*	**a*)**	tagtcgccgc	*57*	**23**	>	11	10^{-6}	A		↑	
	rs994253512	cccgcctgcc	*g*	**a**	tgtagtcgcc	*57*	**35**	>	6	10^{-6}	A		↑	
	rs1017071883	tccgacagcc	*g*	**a**	gaagtcccgc	*57*	**37**	>	7	10^{-6}	A		↑	
	rs112633452	gacgcgccac	*g*	**t**	gccggttgct	*57*	**29**	>	10	10^{-6}	A		↑	
	rs542157051	ccccctcccc	*c*	**a, t*)**	actcgggcgg	*203*	**101**	>	13	10^{-6}	A		↑	
	rs770823189	ggcccggctg	*c*	**t*)**	tgggggctgg	*203*	**127**	>	9	10^{-6}	A		↑	
	rs746876136	tcgtcggccc	*g*	**a, t**	gctgctgggg	*203*	**176**	>	3	0.05	D		↑	
	rs8192516:**a**	tcccccactc	*g*	**a**	ggcggccctg	*203*	**111**	>	11	10^{-6}	A		↑	
	rs8192516:**c**	tcccccactc	*g*	**c**	ggcggccctg	*203*	**271**	<	5	10^{-6}	A	weakened neutrophil differentiation, autophagic flux, and wound healing	↓	
	rs764611659	ggcgccctga	*c*	**−**	aaatggcgcc	*21*	**39**	<	12	10^{-6}	A		↓	
	rs895359331	cccggccggt	*t*	**c**	gctaggctcc	*57*	**109**	<	9	10^{-6}	A		↓	
	1055792704	ccccggccgg	*t*	**−**	tgctaggctc	*57*	**80**	<	5	10^{-6}	A		↓	

Supplementary Material

Table S2. Continued

Gene, OMIM	dbSNP (Sherry et al., 2001)	5′ flank	wt	**mut**	3′ flank	K_D, nM, prediction						**Known physiological or** candidate SNP$ markers	Ss	**ClinVar** or Reference
						wt	**mut**	Δ	Z	α	ρ			
GABBR1, 603540	rs550307496	agggccccgg	*g*	**a**	ttagcagggc	*56*	**36**	>	5	10^{-6}	A	reduced initiation of pathological forms of pain	↑	Hanack et al., 2015
	rs111846609	gccccgggtt	*a*	**t**	gcagggctcg	*56*	**77**	<	5	10^{-3}	B	increased depression-like behavior	↓	Fatemi et al., 2011
GABBR2, 607340	rs916566047	tccctggagg	*c*	**t**	ggcccgagcc	*213*	**165**	>	5	10^{-3}	B	accelerated compensatory recovery of vestibular reflexes after injury	↑	Heskin-Sweezie et al., 2010
	rs938493377	ggctcgtccc	*c*	**a**	gtccctggag	*213*	**157**	>	6	10^{-6}	A		↑	
	rs886299937	cccccggcgc	*g*	**t**	cacggctcgt	*213*	**143**	>	8	10^{-6}	A		↑	
	rs1016019627	cagcgcctcc	*c*	**a**	cctcccccgg	*213*	**158**	>	6	10^{-6}	A		↑	
	rs576859190:**t**	gcgcacggct	*c*	**t*)**	gtccccgtcc	*213*	**100**	>	14	10^{-6}	A		↑	
	rs576859190:**g**	gcgcacggct	*c*	**g*)**	gtccccgtcc	*213*	**243**	<	2	0.05	D	increased depression-like behavior	↓	Fatemi et al., 2011
GABRA1, 137160	rs1023408948	ccggtcttaa	*g*	**t**	agatcctgtg	*10*	**4**	>	13	10^{-6}	A	increased anxiety-related behavior	↑	Liu et al., 2018
	rs773487546	ctcacccttt	*c*	**t**	taccсttccc	*18*	**11**	>	4	10^{-3}	B		↑	
	rs949920683	taagtgcagt	*c*	**a**	cgtatgtctc	*18*	**4**	>	15	10^{-6}	A		↑	
	rs905006300	ccctccaatc	*c*	**t**	ctaaatatgt	*7*	**6**	>	2	0.05	D		↑	
	rs1055875025	ggacttagac	*a*	**t**	tatgtgtggg	*4*	**6**	<	6	10^{-6}	A	reduced anxiety-related behavior	↓	Innos et al., 2011
	rs930737705	ggggacttag	*ac*	**−**	atatgtgtgg	*4*	**5**	<	2	0.05	D		↓	
	rs752967542	ttcctagtgt	*a*	**g**	taattatttg	*3*	**5**	<	8	10^{-6}	A		↓	
	rs564399212	caatccctaa	*a*	**g**	tatgttcttc	*7*	**9**	<	4	10^{-3}	B		↓	
GABRA2, 137140	rs114708494	ccatcaccgc	*c*	**t*)**	actacgaacg	*14*	**10**	>	5	10^{-3}	B	alleviated post-traumatic neuropathic pain	↑	Obradovic et al., 2015
	rs960751751	cgcgccttct	*c*	**a**	ttattgtgat	*7*	**5**	>	6	10^{-6}	A		↑	
	rs199761258	tctctctctc	*c*	**a, g**	caagtttcct	*61*	**54**	>	2	0.05	D		↑	
	rs957212598	ctctctctct	*c*	**a**	ccaagtttcc	*61*	**15**	>	23	10^{-6}	A		↑	
	rs199528096	ctctctctct	*c*	**g**	tctctctctc	*61*	**47**	>	5	10^{-6}	A		↑	
	rs200529996	ctctctctct	*c*	**t**	tctctctctc	*61*	**32**	>	12	10^{-6}	A		↑	
	rs113651998	ctctctctct	*c*	**g**	tctctctctc	*61*	**47**	>	5	10^{-6}	A		↑	
	rs750040856	ttttaagtgt	*a*	**g**	ttttgttttt	*6*	**7**	<	4	10^{-3}	B	aggravated post-traumatic neuropathic pain	↓	
	rs3775288	tctctctctc	*a*	**c**	cccaagtttc	*61*	**76**	<	4	10^{-3}	B		↓	
	rs199684976	tctctctctc	*t*	**c**	ctcccaagtt	*61*	**76**	<	4	10^{-3}	B		↓	
	rs773472878	tctctctctc	*11 bp*	**−**	ctcccaagtt	*61*	**76**	<	4	10^{-3}	B		↓	
	rs200969140	tctctctctc	*t*	**c**	ctctcccaag	*61*	**76**	<	4	10^{-3}	B		↓	
GABRA5, 137142	rs757228114	gtcattaatg	*c*	**t**	caccctcacc	*10*	**7**	>	5	10^{-3}	B	increased anxiety-like behavior	↑	Liu et al., 2018
	rs992131156	gtgactacac	*g*	**a**	aggcgccgaa	*17*	**12**	>	4	10^{-3}	B		↑	
	rs930909892	cgaggtgaga	*12 bp*	**−**	gcgccggggg	*89*	**21**	>	26	10^{-6}	A		↑	
	rs1048063014	gagcgggcgc	*g*	**t**	agtgcgccgg	*89*	**47**	>	10	10^{-6}	A		↑	
	rs939688720	agcgcggggc	*g*	**a**	caagagccgc	*117*	**31**	>	26	10^{-6}	A		↑	
	rs960374140	ggtgtcgcgc	*c*	**a, t**	cgtgtcgcgc	*140*	**68**	>	13	10^{-6}	A		↑	
	rs61996950	accccgcggg	*g*	**a**	gtgtcgcgcg	*140*	**116**	>	3	10^{-2}	C		↑	
	rs1005678287	gcgctgctcg	*c*	**t*)**	ggggtggact	*140*	**109**	>	4	10^{-3}	B		↑	
	rs891159550	gtttttgtgt	*g*	**a**	tttaaaccaa	*14*	**7**	>	15	10^{-6}	A		↑	
	rs914784544	tctaccggag	*c*	**t**	acctctgcag	*47*	**25**	>	8	10^{-6}	A		↑	
	rs991518172	ggcctctacc	*g*	**a**	gagcacctct	*47*	**32**	>	5	10^{-6}	A		↑	
	rs546376304	atttgctgag	*c*	**t**	gtctggcggc	*47*	**33**	>	6	10^{-6}	A		↑	
	rs780665732	ctgtttctta	*a*	**g**	atgtgctttt	*7*	**12**	<	10	10^{-6}	A	increased autism-like aggressive behavior	↑	Zurek et al., 2017
	rs576189672	agagacgaca	*t*	**g*)**	gtggcgctcg	*20*	**53**	<	18	10^{-6}	A		↑	
GABRA6, 137143	rs745580098	acctttttaca	*t*	**c**	tgaggatgga	*7*	**5**	>	4	10^{-3}	B	reduced risks of stress-induced adolescent depression-like behavior	↑	Yang L. et al., 2016
	rs776986809	aactgagatt	*c*	**t**	tgagtctgaa	*30*	**28**	>	4	10^{-3}	B		↑	
	rs775718956	ttgaagtttg	*g*	**t**	ggggccaact	*30*	**27**	>	4	10^{-3}	B		↑	
	rs143274925	gttgaagttt	*g*	**a**	gggggccaac	*30*	**23**	>	4	10^{-3}	B		↑	
	rs745960182	agaggttgaa	*g*	**a**	tttggggggc	*30*	**25**	>	4	10^{-3}	B		↑	
	rs375602341	atgagaggtt	*g*	**a, t**	aagtttgggg	*30*	**11**	>	4	10^{-3}	B		↑	
	rs3811995	cgaccatgca	*c*	**t**	tgtcccttca	*41*	**47**	<	4	10^{-3}	B	reduced motor behavior	↓	Kim et al., 2015

Table S2. Continued

Gene, OMIM	dbSNP (Sherry et al., 2001)	5′ flank	wt	**mut**	3′ flank	K_D, nM, prediction						**Known physiological or** candidate SNP[$] markers	Ss	**ClinVar** or Reference
						wt	**mut**	Δ	Z	α	ρ			
GABRB1, 137190	rs190689458	gagctatgta	*c*	**t**	accgctccac	*2*	**4**	<	8	10^{-6}	A	increased depression-like behavior	↓	Fatemi et al., 2013
	rs1011518533	atgtagagct	*a*	**c**	tgtataccgc	*2*	**4**	<	9	10^{-6}	A		↓	
GABRB2, 600232	rs943953551	tcttttgtga	*a*	**c**	ttgagtatct	*17*	**15**	>	2	0.05	D	predisposition to accelerated developing brain injury	↓	Bo et al., 2008
	rs757037702	tcaattccat	*c*	**a**	aaaaactaaa	*16*	**8**	>	9	10^{-6}	A		↓	
	rs199977195	ccctcaattc	*c*	**t**	atcaaaaact	*16*	**9**	>	6	10^{-6}	A		↓	
	rs749615089	gcagactaag	*t*	**g**	tggatctcct	*16*	**19**	<	2	0.05	D	stress-induced hyperalgesia	↓	Ma et al., 2014
	rs537576321	tcacattaga	*a*	**c**	ttactgcact	*8*	**10**	<	3	10^{-3}	B		↓	
	rs571663848	ctcacattag	*a*	**c**	attactgcac	*8*	**10**	<	3	10^{-2}	C		↓	
GABRB3, 137192	rs1051166786	ggggtagggg	–	**6 bp**	cggggatccc	*119*	**69**	>	8	10^{-6}	A	increased autism-like aggressive behavior	↑	Tunc-Ozcan et al., 2013
	rs761859370	ggaagacggg	*25 bp*	**–**	ggtgggggta	*119*	**88**	>	5	10^{-6}	A		↑	
	rs956348540	tccttcctct	*c*	**t**	ccagcgcccg	*109*	**83**	>	5	10^{-6}	A		↑	
	rs989453794	cctccttcct	*c*	**g**	tcccagcgcc	*109*	**66**	>	10	10^{-6}	A		↑	
	rs933451190	agatgtaact	*c*	**t**	aggatttcac	*9*	**6**	>	6	10^{-6}	A		↑	
	rs987859921	agcctcccca	*g*	**t**	atgtaactca	*9*	**3**	>	14	10^{-6}	A		↑	
	rs575205682	gggctgtcat	*t*	**a*)**	tggtggaggt	*39*	**23**	>	25	10^{-6}	A		↑	
	rs1023234060	gggtgggggt	*a*	**g**	ggggcgggga	*119*	**149**	<	3	10^{-3}	B	stress-induced hyperalgesia	↓	Ma et al., 2014
	rs968769457	ggggtggggg	*t*	**g**	aggggcgggg	*119*	**149**	<	3	10^{-3}	B		↓	
	rs201598082	ctgggctgtc	*a*	**c, g**	tttggtggag	*39*	**50**	<	5	10^{-3}	B		↓	
	rs386782327	gctgggctgt	*ca*	**gg**	tttggtggag	*39*	**53**	<	6	10^{-6}	A		↓	
	rs931732521	cctctcctgt	*a*	**g**	aagagcagag	*7*	**29**	<	21	10^{-6}	A		↓	
GABRD, 137163	rs910664464	tccgcccccct	*c*	**t**	gcgcccgcgc	*227*	**178**	>	4	10^{-3}	B	anorexia nervosa-related behaviors in adolescents	↓	Aoki et al., 2012
GABRE, 300093	rs757008061	gtgggaggag	*t*	**g**	gaaagttgga	*41*	**59**	<	7	10^{-6}	A	improved heart rate due to baroreceptor reflex	↑	Irnaten et al., 2002
	rs780719159	gagtgggagg	*a*	**g**	gtgaaagttg	*41*	**48**	<	3	10^{-2}	C		↑	
GABRG1, 137166	rs935859588	tctttttaag	*a*	**g**	taatttatgc	*7*	**5**	>	6	10^{-6}	A	reduced autism-like aggressive behavior	↓	Kratsman et al., 2016
GABRG2, 137164	rs190756358	tccactttga	*c*	**t**	ctcatagtta	*6*	**4**	>	4	10^{-3}	B	infancy-like stress-inducible anxiety-related behavior	↑	van der Kooij et al., 2015
	rs370241190	cctcatagtt	*a*	**c**	atctgcgtga	*6*	**7**	<	2	0.05	D	increased depression-like behavior	↓	Ren et al., 2015
	rs369703101	cttccacttt	*g*	**a**	acctcatagt	*6*	**7**	<	2	0.05	D		↓	
GABRG3, 600233	rs936068495	ggctccaggc	*g*	**a, t**	gggtgggcgg	*325*	**261**	>	4	10^{-3}	B	higher risk of hyperacusis up to depression	↓	Manohar et al., 2016
	rs1002152544	gtccagtgtg	*c*	**t**	gccccgcggg	*74*	**34**	>	13	10^{-6}	A		↓	
	rs370040504	ttttcctgaa	*t*	**c**	tatttttgac	*14*	**10**	>	6	10^{-6}	A		↓	
	rs780304405	tggctttttc	*g*	**a**	caggctcacc	*34*	**28**	>	4	10^{-3}	B		↓	
	rs374839752	ttggcttttt	*c*	**t**	gcaggctcac	*34*	**22**	>	10	10^{-6}	A		↓	
	rs4128126	gctagactga	*c*	**t**	acttggcttt	*34*	**10**	>	19	10^{-6}	A		↓	
	rs369393344	ctgagctaga	*c*	**t**	tgacacttgg	*34*	**24**	>	7	10^{-6}	A		↓	
	rs374930387	gctgtgtgct	*g*	**a**	agctagactg	*34*	**21**	>	8	10^{-6}	A		↓	
	rs182948702	gccttgccct	*g*	**a*)**	tgctgtgtgc	*34*	**29**	>	3	10^{-2}	C		↓	
	rs1027747836	ccggcgtccc	*11bp*	**–**	ccggcgtccc	*74*	**93**	<	4	10^{-3}	B	weak feeling of fatigue caused by aerobic loads	↑	Kawai et al., 2007
	rs1025367023	cccggcgtcc	*c*	**g**	gtgtgcgtcc	*74*	**84**	<	2	0.05	D		↑	
	rs1024303007	ccccaggcca	*c*	**a*)**	gccctttagg	*31*	**35**	<	2	0.05	D		↑	
GABRP, 602729	rs1038021638	atatcccata	*c*	**t**	ttgtgggtta	*12*	**10**	>	4	10^{-3}	B	hypertension-related behavior	↓	Sadeghi, Taylor, 2010
	rs1014674604	gatgccagtg	*t*	**a**	taaggaccgg	*10*	**12**	<	3	10^{-2}	C	poorly controlled antipsychotic-like moodless behavior	↓	Soggiu et al., 2016
	rs181978913	tcccatactt	*g*	**t**	tgggttaagg	*12*	**14**	<	2	0.05	D		↓	
	rs184825634	actaataaag	*g*	**t**	ctggtctgtt	*5*	**6**	<	4	10^{-3}	B		↓	
GABRR1, 137161	rs952855254	gtgggttgat	*t*	**a**	ttttttttgc	*30*	**16**	>	10	10^{-6}	A	higher threshold for mechanical pain	↑	Zheng et al., 2003
GABRR2, 137162	rs114956766	tatcaaatag	*t*	**c**	aaatcgggac	*4*	**7**	<	7	10^{-6}	A	higher threshold for mechanical pain	↑	Tadavarty et al., 2015
GABRR3, no OMIM ID	rs777811241	ctgttttttt	*g*	**a**	ttttggaaga	*15*	**6**	>	14	10^{-6}	A	brain hypersusceptibility to excitotoxicity up to seizures	↓	Michalovicz, Konat, 2014
	rs762387385	cagtattggt	*g*	**a**	gcgttcacct	*15*	**12**	>	4	10^{-3}	B		↓	
	rs774794406	acagtattgg	*t*	**c**	tgcgttcacc	*15*	**19**	<	4	10^{-3}	B	worse healing of wounds owing to accelerated platelet senescence	↓	Pienimaeki-Roemer et al., 2017
	rs762344579	gatcacagta	*t*	**c**	tggttgcgtt	*15*	**20**	<	5	10^{-6}	A		↓	

Supplementary Material

Table S2. Continued

Gene, OMIM	dbSNP (Sherry et al., 2001)	5′ flank	wt	**mut**	3′ flank	K_D, nM, prediction						**Known physiological or** candidate SNP$ markers	Ss	**ClinVar** or Reference
						wt	**mut**	Δ	Z	α	ρ			
GPR18, 602042	rs966307447	aagtcataag	*g*	**a**	tgaaaaagcc	*13*	**8**	>	7	10^{-6}	A	increased pain threshold for mechanical stimuli	↑	Malek et al., 2016
	rs976712436	taaagtcata	*a*	**g**	ggtgaaaaag	*13*	**16**	<	4	10^{-3}	B	worse post-injury repair of microvascular endothelial cells	↓	Zuo et al., 2018
	rs543924530	tgtgacttca	*t*	**c**	aaacaaatca	*7*	**17**	>	16	10^{-6}	A		↓	
GPR55, 604107	rs189675005	ggcagataag	*g*	**a**	ccctggtaag	*13*	**11**	>	3	10^{-2}	C	reduced pain sensitivity	↑	Naderi et al., 2012
	rs181075597	cttcccctag	*g*	**a**	gcagataagg	*13*	**11**	>	3	10^{-2}	C		↑	
	rs528182559	actctcccca	*g*	**t**	aacaggctga	*30*	**15**	>	12	10^{-6}			↑	
	rs3106075:**t**	tcccctaggg	*c*	**t**	agataaggcc	*13*	**4**	>	17	10^{-6}	A		↑	
	rs3106075:**g**	tcccctaggg	*c*	**g**	agataaggcc	*13*	**16**	<	3	10^{-3}	B	increased anxiety-related behavior	↑	Rahimi et al., 2015
GPR119, 300513	rs997396487	ggccagaatc	*g*	**a**	tgctgtagct	*49*	**32**	>	7	10^{-6}	A	reduced need for food, body weight gain, and fat deposition	↓	Overton et al., 2006
	rs185026986	ggatcccaaa	*g*	**a**	atggcgacct	*49*	**28**	>	10	10^{-6}	A		↓	
	rs538711592	agaatcgtgc	*t*	**c**	gtagctctga	*49*	**67**	<	5	10^{-6}	A	increased need for food, body weight gain, and fat deposition	↑	
	rs552021542	agcgaaggcc	*a*	**g**	gaatcgtgct	*49*	**56**	<	2	0.05	D		↑	
GRIA1, 138248	rs990289481	aggaggaaaa	*g*	**t**	aacaggcaga	*36*	**12**	>	18	10^{-6}	A	increased anxiety-like behavior	↑	Liu et al., 2018
	rs958843138	actgcaggag	*g*	**a**	aaaagaacag	*36*	**23**	>	10	10^{-6}	A		↑	
	rs755176176	gtgctctttt	*g*	**t**	tgagtgtgtg	*12*	**10**	>	4	10^{-3}	B		↑	
	rs903431204	ttgtgctctt	*t*	**c**	tgtgagtgtg	*12*	**15**	<	4	10^{-3}	B	increased anxiety-like behavior	↑	Mead et al., 2006
	rs764546765	gtttgtgctc	*t*	**−**	tttgtgagtg	*12*	**15**	<	4	10^{-3}	B		↑	
GRIA2, 138247	rs575850260	gagtgtgtgt	*g*	**a**	agtgcatggg	*20*	**7**	>	18	10^{-6}	A	increased depression-like behavior	↓	Gray et al., 2015
	rs1033352853	gcgcgtgagt	*ga*	**−**	gagaggagag	*18*	**17**	>	2	0.05	D		↓	
	rs60010721	tgtgtgtgtg	*tgtg*	**−**	cgcgcgcgcg	*18*	**13**	>	7	10^{-6}	A		↓	
	rs71942646	gtgtgtgtgt	*gtgt*	**−**	gtgtgtgcgc	*18*	**13**	>	7	10^{-6}	A		↓	
	rs907472385	gtgtgtgtgt	*g*	**a**	tgtgtgtgcg	*18*	**6**	>	20	10^{-6}	A		↓	
	rs1014023735	gtgtgtatgt	*g*	**a**	tgtgtgtgtg	*18*	**13**	>	7	10^{-6}	A		↓	
	rs113291896	cgtgtgtgta	*tgtg*	**−**	tgtgtgtgtg	*18*	**13**	>	7	10^{-6}	A		↓	
	rs964891373	gcacaatttt	*g*	**a**	gaatatttcc	*16*	**10**	>	6	10^{-6}	A		↓	
	rs543053885	cccaagtgca	*c*	**t**	aattttggaa	*16*	**12**	>	3	10^{-2}	C		↓	
	rs1046283273	atgactctac	−	**a**	aagggaggga	*8.2*	**7.6**	>	3	0.05	D		↓	
	rs895627013	gcgtgtgtgt	*a*	**g**	tgtgtgtgtg	*6*	**19**	<	21	10^{-6}	A	reduced anxiety-like behavior	↓	Mead et al., 2006
	rs1045963804	tgtgtgtgcg	*8 bp*	**−**	tgtgtgtgtg	*6*	**20**	<	20	10^{-6}	A		↓	
	rs868602399	atatttcctc	*c*	**t**	acggtgcttt	*16*	**20**	<	2	0.05	D		↓	
	rs561017052	aattttggaa	*t*	**g**	atttcctcca	*16*	**38**	<	11	10^{-6}	A		↓	
	rs777916396	gggtgctgaa	*t*	**g**	attccgagac	*23*	**80**	<	20	10^{-6}	A		↓	
	rs957068306	atgtgaaatt	*a*	**g**	tagactcatc	*6*	**9**	<	8	10^{-6}	A		↓	
	rs187499599	aatgtgaaat	*t*	**c**	atagactcat	*6*	**9**	<	8	10^{-6}	A		↓	
GRIA4, 138246	rs897075614	ccattccctt	*g*	**a**	tcaggctctc	*56*	**22**	>	14	10^{-6}	A	increased depression-like behavior	↓	Gray et al., 2015
	rs533286585	catccattcc	*c*	**g**	ttgtcaggct	*56*	**40**	>	7	10^{-6}	A		↓	
	rs754073551	accctttcac	*c*	**t**	tcatccattc	*56*	**28**	>	15	10^{-6}	A		↓	
	rs771981314	taggaagagt	*g*	**a**	cgagagaaag	*24*	**14**	>	9	10^{-6}	A		↓	
	rs370439405	cagcctttag	*g*	**t**	aagagtgcga	*24*	**4**	>	22	10^{-6}	A		↓	
	rs568903088	tttctcctcc	*c*	**t**	atccctctct	*29*	**26**	>	2	0.05	D		↓	
	rs564357154	cgcagcccag	*t*	**c**	ggcagaagag	*44*	**54**	<	4	10^{-3}	B	reduced fright behavior	↑	Sagata et al., 2010
	rs765551394	gcgagcagcc	*a*	**g**	ctagacgctc	*21*	**34**	<	8	10^{-6}	A		↑	
GRIK1, 138245	rs868444269	cctcgagctc	*g*	**t**	ctccgtgggg	*182*	**117**	>	9	10^{-6}	A	hyperresponsiveness to itching and scratch pain	↓	Descalzi et al., 2013
	rs1026990182	cgcggtccca	*c*	**t***)	gcgggttccc	*182*	**107**	>	11	10^{-6}	A		↓	
	rs758307624	ctccgcggtc	*c*	**t**	cacgcgggtt	*182*	**81**	>	16	10^{-6}	A		↓	
	rs754473482	atgggctagc	*g*	**t**	acagcactga	*52*	**24**	>	11	10^{-6}	A		↓	
	rs928450268	gccgtgatgg	*g*	**a**	ctagcgacag	*52*	**40**	>	4	10^{-3}	B		↓	
	rs981379230	cagcactgag	*g*	**c**	agccccgaga	*52*	**57**	<	2	0.05	D	reduced sensitivity to pain	↑	Kolber et al., 2010

Supplementary Material

Table S2. Continued

Gene, OMIM	dbSNP (Sherry et al., 2001)	5′ flank	wt	**mut**	3′ flank	K_D, nM, prediction						**Known physiological or** candidate SNP[$] markers	Ss	**ClinVar** or Reference
						wt	**mut**	Δ	Z	α	ρ			
GRIK2, 138244	rs781118131	ggatgcaaaa	*c*	**a**	ccttactaaa	*6*	**5**	>	2	0.05	D	hypersensitivity to acute visceral pain	↓	Zhang et al., 2009
	rs372164020	gagccgaacg	*c*	**a**	tagatcgggg	*23*	**14**	>	8	10^{-6}	A		↓	
	rs781324413	ggagccgaac	*g*	**a**	ctagatcggg	*23*	**14**	>	8	10^{-6}	A		↓	
	rs757312004	aacccttact	*a*	**g**	aaagaaatga	*6*	**11**	<	12	10^{-6}	A	higher risks of mania-like aggressive behavior	↑	Shaltiel et al., 2008
	rs751419152	caaagtacat	*a*	**g**	tgctatttta	*3*	**6**	<	10	10^{-6}	A		↑	
	rs766247996	tttacaaagt	*a*	**t**	catatgctat	*3*	**8**	<	13	10^{-6}	A		↑	
	rs762797067	atttacaaag	*t*	**a**	acatatgcta	*3*	**10**	<	16	10^{-6}	A		↑	
	rs369245315	aggagccgaa	*c*	**t**	aggagccgaa	*23*	**30**	<	4	10^{-3}	B		↑	
	rs575125829	ggttttaagc	*a*	**g**	actgcccaaa	*9*	**10**	<	2	0.05	D		↑	
GRIK3, 138243	rs924976738	gcgcctagag	*c*	**t**	tgccgcccca	*52*	**31**	>	7	10^{-6}	A	increased depression-like behavior	↓	Gray et al., 2015
GRIK5, 600283	rs772879825	agctgcgtcc	*c*	**g**	catgaggagg	*58*	**45**	>	5	10^{-3}	B	reduced depression-like behavior	↑	Xu et al., 2017a
	rs774180633	gcggcagctg	*c*	**t**	gtccccatga	*58*	**49**	>	3	10^{-2}	C		↑	
	rs369605813	cgcggcagct	*g*	**a**	cgtccccatg	*58*	**26**	>	12	10^{-6}	A		↑	
	rs749204366	ggcagctgcg	*t*	**c**	ccccatgagg	*58*	**70**	>	3	10^{-2}	C	increased depression-like behavior	↓	
GRIN1, 611239	rs955395023	gcgccgcgca	*g*	**a**	agccaggccc	*203*	**107**	>	13	10^{-6}	A	increased impulsiveness	↑	Reif et al., 2009
	rs150851773	cgttcgcgcc	*g*	**a*)**	cgcagagcca	*203*	**128**	>	9	10^{-6}	A		↑	
	rs200150234	gccccgcgtt	*c*	**a**	gcgccgcgca	*203*	**61**	>	19	10^{-6}	A		↑	
	rs753166193	gcgttcgcgc	*c*	**t**	gcgcagagcc	*203*	**160**	>	5	10^{-3}	B		↑	
	rs540882401	cgcgttcgcg	*c*	**a**	cgcgcagagc	*203*	**157**	>	5	10^{-6}	A		↑	
	rs964029911	agggccccgc	*g*	**a**	ttcgcgccgc	*203*	**131**	>	9	10^{-6}	A		↑	
	rs759827008	gagggccccg	*c*	**a**	gttcgcgccg	*203*	**147**	>	6	10^{-6}	A		↑	
	rs372970942	ccgagggccc	*c*	**t**	gcgttcgcgc	*203*	**153**	>	6	10^{-6}	A		↑	
	rs201254060	caaagacagg	*g*	**a**	tggtgtggac	*23*	**26**	<	2	0.05	D	reduced impulsiveness	↓	
GRIN2A, 138253	rs542854991	aggggagctc	*c*	**t**	tagggcttgg	*56*	**35**	>	4	10^{-3}	B	more stable long-term fear memories	↓	Holehonnur et al., 2016
	rs569856570	tctttctctc	*c*	**t**	ctacctccct	*26*	**23**	>	4	10^{-3}	B		↓	
	rs1046484661	gagctaggga	*t*	**c**	cttggagggg	*56*	**94**	<	4	10^{-3}	B	worse spatial learning and memory during early postnatal stress	↓	Hu et al., 2016
	rs891920545	cataaaatct	*a*	**g**	tttgtctgtc	*12*	**29**	<	4	10^{-3}	B		↓	
GRIN2C, 138254	rs890182922	ccgccgccgc	–	**12 bp, cgc**	atcgcgagtg	*201*	**760**	<	27	10^{-6}	A	reduced conditioned fear	↑	Hillman et al., 2011
	rs557742160	ccgccgccgc	–	**9 bp**	cgcatcgcga	*201*	**760**	<	27	10^{-6}	A		↑	
GRIN2D, 602717	rs181253715	gagccctcct	*c*	**a*)**	ggaatcttgg	*72*	**31**	>	14	10^{-6}	A	higher neuropathic allodynia as pain sensitivity	↓	Kaneko et al., 2010
	rs543803748	cccccgacat	*c*	**t**	ggctctctga	*72*	**64**	>	2	0.05	D		↓	
	rs142614763	tgcccccga	*c*	**t**	atcggctctc	*72*	**25**	>	16	10^{-6}	A		↓	
	rs550069994	gcctgccccc	*c*	**t**	gacatcggct	*72*	**48**	>	7	10^{-6}	A		↓	
GRIN3A, 606650	rs963601907	ggaaaaataa	*c*	**a**	gaaagaaagg	*18*	**10**	>	10	10^{-6}	A	careful behavior	↓	dela Pena et al., 2015
GRIN3B, 606651	rs535918061	ccgtggtccc	*g*	**t*)**	gtggttgcgc	*139*	**64**	>	13	10^{-6}	A	reduced anxiety-like behavior	↓	Niemann et al., 2007
	rs748790446	gccgtggtcc	*c*	**t**	ggtggttgcg	*139*	**77**	>	10	10^{-6}	A		↓	
	rs779743154	cgccgtggtc	*c*	**t**	cggtggttgc	*139*	**108**	>	4	10^{-3}	B		↓	
	rs568854882	tcggaacctg	*g*	**a**	ccggggccct	*139*	**108**	>	4	10^{-3}	B		↓	
GRINA, 138251	rs782722525	tggccccagc	*29 bp*	**–**	tccctacccc	*68*	**50**	>	5	10^{-3}	B	increased depression-like behavior	↓	Goswami et al., 2013
	rs782755338	agcccctacc	*c*	**a**	ccaaggggc	*68*	**30**	>	12	10^{-6}	A		↓	
	rs7814596	gccccagccc	*c*	**t**	tacccccaag	*68*	**40**	>	8	10^{-6}	A		↓	
	rs782786023	ggccatatcc	*c*	**t**	cagagcccct	*26*	**21**	>	3	10^{-3}	B		↓	
	rs782085222	acccccaggg	*g*	**t**	ccatatcccc	*26*	**18**	>	6	10^{-6}	A		↓	
	rs782761973	catcctacta	*t*	**c**	gacaaccagg	*13*	**9**	>	5	10^{-3}	B		↓	
	rs782525102	ggtcccccat	*c*	**t**	ctactatgac	*13*	**8**	>	7	10^{-6}	A		↓	
	rs540702356	gccccgccca	*c*	**t**	ctgcccctcc	*147*	**80**	>	11	10^{-6}	A		↓	
	rs782380662	cgccccctgc	*25 bp*	**–**	cgtcttccga	*48*	**136**	<	19	10^{-6}	A	increased anti-epileptic behavior	↑	Li et al., 2014
	rs547176289	cggcagccga	*t*	**g**	ccaggcccga	*137*	**263**	<	13	10^{-6}	A		↑	
	rs868935561	ccagggggcca	*t*	**c**	atccccagag	*26*	**76**	<	17	10^{-6}	A		↑	

Supplementary Material

Table S2. Continued

Gene, OMIM	dbSNP (Sherry et al., 2001)	5′ flank	wt	**mut**	3′ flank	K_D, nM, prediction						**Known physiological or** candidate SNP[$] markers	Ss	**ClinVar** or Reference
						wt	**mut**	Δ	Z	α	ρ			
GRM1, 604473	rs532615361	`taccttgatg`	*c*	**t**	`actaccggtg`	*15*	**3**	>	21	10^{-6}	A	increased depression-like behavior	↓	Gray et al., 2015
	rs549918628	`tgggcgtctt`	*g*	**t**	`ggggtgcgcg`	*147*	**93**	>	8	10^{-6}	A		↓	
	rs368106110	`tttttttttt`	*t*	**–**	`cttccctccc`	*28*	**24**	>	3	10^{-3}	B		↓	
	rs879928599	`cctgtttttt`	–	**a**	`tttttcttcc`	*28*	**7**	>	27	10^{-6}	A		↓	
GRM2, 604099	rs368654699	`ttgaaagcgg`	*c*	**t**[*)]	`ggggcgagcg`	*33*	**30**	>	2	0.05	D	reduced pain sensitivity	↑	Davidson et al., 2016
	rs1052644999	`ggcggggcgg`	*25 bp*	**–**	`gcgggcgggc`	*33*	**236**	<	38	10^{-6}	A	increased fearful and disgust motivations	↓	Richard, Berridge, 2011
GRM3, 601115	rs372048007	`tctgcacaaa`	*c*	**a**	`cctctccagg`	*36*	**18**	>	13	10^{-6}	A	increased long-term depression behavior	↓	Walker et al., 2015
	rs765210855	`caaccatgag`	*c*	**t**	`cagagcccgg`	*53*	**27**	>	13	10^{-6}	A		↓	
	rs956673948	`tgagtatgac`	*c*	**t**	`ttgtacccac`	*16*	**9**	>	9	10^{-6}	A		↓	
	rs916394201	`tgcaggctca`	–	**6 bp**	`ccgccgccgc`	*53*	**224**	<	25	10^{-6}	A	increased schizophrenia-like hyperactive aggressive behavior	↑	Fujioka et al., 2014
GRM4, 604100	rs866992427	`ctggactgtg`	*c*	**t**[*)]	`gggcaactgt`	*39*	**33**	>	2	0.05	D	increased depression-like behavior	↓	Chandley et al., 2014
	rs372057436	`gtggggagac`	*c*	**–**	`tattattgct`	*7*	**5**	>	3	10^{-2}	C		↓	
	rs79719881	`cgtccctgtg`	*c*	**t**	`tttgctgtac`	*64*	**41**	>	7	10^{-6}	A		↓	
	rs530977254	`agtgacgcac`	*c*	**t**	`agggtgggag`	*64*	**31**	>	11	10^{-6}	A		↓	
	rs186811870	`gagctgccag`	*g*	**a**	`caagacagat`	*39*	**31**	>	5	10^{-3}	B		↓	
	rs564769797	`gtcctcccgc`	*a*	**g**	`cacaatctgg`	*17*	**35**	<	3	10^{-3}	B	reduced microglia-related neuroinflammatory response in post-injury brain repair	↑	Ponnazhagan et al., 2016
	rs932127630	`ggggagacct`	*a*	**g**	`ttattgctcc`	*7*	**12**	<	9	10^{-6}	A		↑	
GRM5, 604102	rs912650405	`gcggcacgtg`	*c*	**t**	`tcccggcgct`	*104*	**39**	>	13	10^{-6}	A	increased novelty-seeking behavior	↑	Leurquin-Sterk et al., 2016
	rs965481347	`agcgcggcac`	*g*	**a**	`tgctcccggc`	*104*	**64**	>	7	10^{-6}	A		↑	
	rs528610	`tttctcagta`	*g*	**c**	`cggggagatt`	*23*	**19**	>	3	10^{-3}	B		↑	
	rs201251830	`atcatctaac`	*t*	**a**	`aaatggtaca`	*7*	**12**	<	6	10^{-6}	A	reduced sensitivity to pain	↑	Lax et al., 2014
	rs201980304	`tggatcatct`	*a*	**g**	`actaaatggt`	*7*	**11**	<	7	10^{-6}	A		↑	
GRM7, 604101	rs762454097	`aacccgagag`	*c*	**g**	`gcgaggcgcc`	*152*	**114**	>	7	10^{-6}	A	increased antidepressant-like behavior	↑	Zhou et al., 2009
	rs935329143	`gctaacccga`	*g*	**a**	`agcgcgaggc`	*152*	**70**	>	14	10^{-6}	A		↑	
	rs746750835	`ccgttccctc`	*c*	**a**	`agcgccgccg`	*153*	**99**	>	8	10^{-6}	A		↑	
	rs565608693	`accgttccct`	*c*	**t**	`cagcgccgcc`	*153*	**121**	>	5	10^{-3}	B		↑	
	rs758162901	`ccaccgttcc`	*c*	**t**	`tccagcgccg`	*153*	**109**	>	7	10^{-6}	A		↑	
	rs540505695	`gcccaccacc`	*g*	**t**	`ttccctccag`	*153*	**99**	>	9	10^{-6}	A		↑	
	rs556828470	`cgctccctag`	*t*	**c**	`gagatgaacc`	*20*	**25**	<	4	10^{-3}	B	increased locomotor activity and anxiety-like behavior, reduced depressive behavior	↑	Cryan et al., 2003
	rs895496662	`ggcgctccct`	*a*	**t**	`gtgagatgaa`	*20*	**33**	<	9	10^{-6}	A		↑	
	rs750812267	`tggcgctccc`	*t*	**c**	`agtgagatga`	*20*	**35**	<	10	10^{-6}	A		↑	
GRM8, *601116*	rs773432214	`agtgataaca`	*c*	**a**	`caggtatgac`	*12*	**10**	>	3	10^{-2}	C	reduced mechanical pain susceptibility	↑	Rossi et al., 2014
	rs771037398	`taagtgataa`	*c*	**t**	`accaggtatg`	*12*	**8**	>	6	10^{-6}	A		↑	
	rs775957882	`aatcagctat`	*g*	**a**	`catccacagc`	*12*	**4**	>	13	10^{-6}	A		↑	
	rs562881546	`tgccttattt`	*g*	**a**	`ccatttgggg`	*24*	**10**	>	12	10^{-6}	A		↑	
	rs983572054	`tggggaatta`	*c*	**g**	`ttcccccgtg`	*40*	**34**	>	3	10^{-2}	C		↑	
	rs1019312719	`cctctcactc`	*t*	**g**	`gtagttgggt`	*11*	**25**	<	11	10^{-6}	A	reduced contextual fear and depression-like behavior such as generalized anxiety-related behavior	↑	Fendt et al., 2013
	rs747249205	`aagtgataac`	*a*	**g**	`ccaggtatga`	*12*	**18**	<	6	10^{-6}	A		↑	
	rs909306022	`ccctggggaa`	*c*	**t**	`tacttccccc`	*40*	**54**	<	5	10^{-6}	A		↑	
	rs191564489	`atgtgaaaca`	*a*	**g**	`gtagacccac`	*11*	**27**	<	18	10^{-6}	A		↑	
HTR1A, 109760	rs932273400:**t**	`gacagacaga`	*c*	**t**	`gttccagcca`	*47*	**26**	>	9	10^{-6}	A	embryonic programming of aggressive behavior	↑	Ahmed et al., 2014
	rs932273400:**g**	`gacagacaga`	*c*	**g**	`gttccagcca`	*47*	**61**	<	4	10^{-3}	C	increased anxiety-like behavior	↑	Meunier et al., 2017
HTR1B, 182131	rs75032335	`tctgcagatc`	*c*	**t**	`aaaagcgtcc`	*37*	**9**	>	24	10^{-6}	A	reduced aggressive behavior	↓	Popova, 2006
	rs924510712	`gtctgcagat`	*c*	**a**	`caaaagcgtc`	*37*	**5**	>	32	10^{-6}	A		↓	
	rs1022356842	`ccaaaagcgt`	*c*	**a**	`caggttagga`	*37*	**22**	>	10	10^{-6}	A		↓	
HTR1F, 182134	rs942605544	`ctgaaaggaa`	*g*	**a**	`agaaaagttc`	*16*	**18**	<	2	0.05	D	reduced pain sensitivity	↑	Granados-Soto et al., 2010

Supplementary Material

Table S2. Continued

Gene, OMIM	dbSNP (Sherry et al., 2001)	5′ flank	wt	**mut**	3′ flank	K_D, nM, prediction						**Known physiological or** candidate SNP[$] markers	Ss	**ClinVar** or Reference
						wt	**mut**	Δ	Z	α	ρ			
HTR2A, 182135	rs6316	tgacatacac	*a*	**g**	tagagggagg	*5*	**7**	<	4	10^{-3}	C	increased aggressive behavior	↑	Popova, 2006
HTR2C, 312861	rs3813929	cccctcatcc	*c*	**t**	gcttttggcc	*73*	**56**	>	6	10^{-6}	A	**olanzapine (antipsychotic) response**<br>*olanzapine-related excess increase in body mass* as well as reduced aggressive behavior	↓	**Landrum et al., 2014**<br>*Ellingrod et al., 2005;* as well as Popova et al., 2010
HTR3A, 182139	rs528460409	cacctgctcc	*g*	**a**	accttcttag	*26*	**29**	<	2	0.05	D	reduced aggressive behavior	↓	Kudryavtseva et al., 2017
	rs200903443	ccacctgctc	*c*	**t**	gaccttctta	*26*	**30**	<	2	0.05	D		↓	
	rs78797932	acagcatgac	*a*	**c**	tcagctaggg	*33*	**54**	<	11	10^{-6}	A		↓	
HTR3B, 604654	rs762131470	ttggcctttg	*a*	**g**	tttattgaca	*7*	**11**	<	4	10^{-3}	B	increased anger-related behavior	↑	Guo et al., 2015
HTR3C, 610121	rs565826563	ctcctttaag	*c*	**t**	tccagggttt	*13*	**8**	>	7	10^{-6}	A	reduced aggressive behavior	↓	Niesler et al., 2007
	rs547673566	gctcctttaa	*g*	**t**	ctccagggtt	*13*	**9**	>	6	10^{-6}	A		↓	
	rs891012877	agctccttta	*a*	**g**	gctccagggt	*13*	**18**	<	6	10^{-6}	A	increased aggressive behavior	↑	
HTR3D, 610122	rs760991487	aggaaggagg	*a*	**g**	gctcacatgc	*30*	**25**	>	3	10^{-2}	C	increased aggressive behavior	↑	
	rs368011858	gatcaaaaat	*c*	**a**	attattgcca	*19*	**9**	>	11	10^{-6}	A		↑	
	rs778053843	gaaggaggag	*c*	**a**	tcacatgctt	*30*	**36**	<	3	10^{-2}	C	reduced aggressive behavior	↓	
HTR3E, 610123	rs776003405	aaagggctta	*g*	**a**	aatatacctg	*5*	**4**	>	3	10^{-3}	B	increased aggressive behavior	↑	
	rs768543061	ggggacgtat	*a*	**g**	gcacagcagc	*6*	**15**	<	10	10^{-6}	A	reduced aggressive behavior	↓	
	rs972175685	cctggggacg	*t*	**c**	atagcacagc	*6*	**32**	<	20	10^{-6}	A		↓	
	rs563391107	gtcccagtac	*a*	**g**	tgttcagaga	*7*	**11**	<	6	10^{-6}	A		↓	
	rs764752724	ggcttagaat	*a*	**g**	tacctgacac	*5*	**6**	<	2	0.05	D		↓	
HTR4, 602164	rs555827116	gcgcaggcag	*c*	**t**	ggctgggagc	*261*	**211**	>	4	10^{-3}	B	reduced aggressive behavior	↓	Kudryavtseva et al., 2017
	rs201301278	ggggcggcag	*g*	**t**	gagggcgcag	*261*	**113**	>	16	10^{-6}	A		↓	
	rs954387142	ccgcacccga	*g*	**a, t**	ggggcggcag	*261*	**173**	>	8	10^{-6}	A		↓	
	rs751705725	aaatccaact	*c*	**t**	actcatgctt	*22*	**14**	>	7	10^{-6}	A		↓	
	rs778825130	tacttccccc	*a*	**g**	ttttaggacc	*22*	**18**	>	4	10^{-3}	B		↓	
	rs201125216	ttacttcccc	*c*	**t**	attttaggac	*22*	**9**	>	16	10^{-6}	A		↓	
	rs772087907	tttacttccc	*c*	**t**	cattttagga	*22*	**20**	>	2	0.05	D		↓	
	rs200189361	catcttttta	*c*	**t**	ttcccccatt	*11*	**7**	>	6	10^{-6}	A		↓	
	rs768118429	cttttataac	*c*	**t**	atctttttac	*11*	**7**	>	6	10^{-6}	A		↓	
	rs761401057	tttcttttat	*a*	**t**	acatcttttt	*11*	**9**	>	2	0.05	D		↓	
	rs763202154	ctgctttctt	*t*	**c, a**	tataacatct	*11*	**6**	>	8	10^{-6}	A		↓	
	rs774677000	gctgctttct	*t*	**a**	ttataacatc	*11*	**6**	>	7	10^{-6}	A		↓	
	rs989274199	ctggcccgcg	*c*	**t**	ctcacgctcg	*143*	**102**	>	7	10^{-6}	A		↓	
	rs746551242	acatcttttt	*a*	**g**	cttcccccat	*11*	**15**	<	5	10^{-3}	B	increased aggressive behavior	↑	
	rs957950340	ggcccgcgcc	*t*	**g**	cacgctcgcc	*143*	**260**	<	12	10^{-6}	A		↑	
HTR5A, 601305	rs371016715	tgatggctta	*c*	**t**	gtagggctag	*6*	**4**	>	6	10^{-6}	A	increased hypertension-related behavior	↓	Farago et al., 2016
HTR7, 182137	rs997150096	agcggaaccg	*g*	**a**	tgaggtgaag	*73*	**48**	>	9	10^{-6}	A	reduced aggressive behavior	↓	Kudryavtseva et al., 2017
SLC6A3, 126455	rs994477499	gtctactgga	*5 bp*	**–**	gcccgaggcc	*9*	**15**	<	8	10^{-6}	A	increased risk-taking behavior	↑	Young et al., 2011
SLC6A4, 182138	rs563815332	ccctcccgca	*a*	**t**	agttaaagag	*7*	**5**	>	8	10^{-6}	A	predisposition to repeated experiencing of social defeats	↓	Kudryavtseva et al., 2017
	rs548873226	gggtacaaat	*a*	**g**	cggccgcgcc	*4*	**5**	<	4	10^{-3}	B	reduced aggressive behavior	↓	Holmes et al., 2002
	rs559905589	ggcccgcggg	*t*	**c**	acaaatacgg	*4*	**7**	<	7	10^{-6}	A		↓	
TH, 191290	rs772061026	agctggacaa	*g*	**t**	tgtcatcacc	*22*	**27**	<	4	10^{-3}	B	reduced aggressive behavior	↓	Young et al., 2011
TPH2, 607478	rs1016706882	ccagtcattc	*a*	**g**	tataaaggag	*2.4*	**2.1**	>	2	0.05	D	reduced aggressive behavior	↓	Audero et al., 2013

Supplementary Material

References

Abiraman, K., Pol, S.U., O'Bara, M.A., Chen, G.D., Khaku, Z.M., Wang, J. et al. (2015) Anti-muscarinic adjunct therapy accelerates functional human oligodendrocyte repair. *J Neurosci*. **35**, 3676-3688. doi: 10.1523/jneurosci.3510-14.2015

Ahmed, A.A., Ma, W., Ni, Y., Zhou, Q., and Zhao, R. (2014) Embryonic exposure to corticosterone modifies aggressive behavior through alterations of the hypothalamic pituitary adrenal axis and the serotonergic system in the chicken. *Horm Behav*. **65**, 97-105. doi: 10.1016/j.yhbeh.2013.12.002

Andersson, K.B., Florholmen, G., Winer, L.H., Tonnessen, T., and Christensen, G. (2006) Regulation of neuronal type genes in congestive heart failure rats. *Acta Physiol (Oxf).* **186,** 17-27. doi: 10.1111/j.1748-1716.2005.01503.x

Aoki, C., Sabaliauskas, N., Chowdhury, T., Min, J.Y., Colacino, A.R., Laurino, K., and Barbarich-Marsteller, N.C. (2012) Adolescent female rats exhibiting activity-based anorexia express elevated levels of GABA(A) receptor α4 and δ subunits at the plasma membrane of hippocampal CA1 spines. *Synapse*. **66**, 391-407. doi: 10.1002/syn.21528

Aridon, P., Marini, C., Di Resta, C., Brilli, E., De Fusco, M., Politi, F. et al. (2006) Increased sensitivity of the neuronal nicotinic receptor alpha 2 subunit causes familial epilepsy with nocturnal wandering and ictal fear. *Am J Hum Genet*. **79**, 342-350. doi: 10.1086/506459

Audero, E., Mlinar, B., Baccini, G., Skachokova, Z.K., Corradetti, R., and Gross, C. (2013) Suppression of serotonin neuron firing increases aggression in mice. *J Neurosci*. **33**, 8678-8688. doi: 10.1523/jneurosci.2067-12.2013

Azadmarzabadi, E., Haghighatfard, A., and Mohammadi, A. (2018) Low resilience to stress is associated with candidate gene expression alterations in the dopaminergic signalling pathway. *Psychogeriatrics*. **18**, 190-201. doi: 10.1111/psyg.12312

Azzinnari, D., Sigrist, H., Staehli, S., Palme, R., Hildebrandt, T., Leparc, G. et al. (2014) Mouse social stress induces increased fear conditioning, helplessness and fatigue to physical challenge together with markers of altered immune and dopamine function. *Neuropharmacology*. **85**, 328-341. doi: 10.1016/j.neuropharm.2014.05.039

Bo, T., Wang, T.M., Zhu, X.H., Li, J., Li, X.F., Chen, Y., and Mao, D.A. (2008) Short-term effects of recurrent neonatal seizures on gamma-aminobutyric acid A receptor alpha1 and beta2 subunit expression in the rat brain. *Zhongguo Dang Dai Er Ke Za Zhi*. **10**, 371-375.

Bowers, M.E., and Ressler, K.J. (2016) Sex-dependence of anxiety-like behavior in cannabinoid receptor 1 (Cnr1) knockout mice. *Behav Brain Res*. **300**, 65-69. doi: 10.1016/j.bbr.2015.12.005

Brigger, D., Torbett, B.E., Chen, J., Fey, M.F., and Tschan, M.P. (2013) Inhibition of GATE-16 attenuates ATRA-induced neutrophil differentiation of APL cells and interferes with autophagosome formation. *Biochem Biophys Res Commun*. **438**, 283-288. doi: 10.1016/j.bbrc.2013.07.056

Budiono, B.P., See Hoe, L.E., Brunt, A.R., Peart, J.N., Headrick, J.P., and Haseler, L.J. (2016) Coupling of myocardial stress resistance and signalling to voluntary activity and inactivity. *Acta Physiol (Oxf).* **218**, 112-122. doi: 10.1111/apha.12710

Burniston, J.G., Meek, T.H., Pandey, S.N., Broitman-Maduro, G., Maduro, M.F., Bronikowski, A.M. et al. (2013) Gene expression profiling of gastrocnemius of "minimuscle" mice. *Physiol Genomics*. **45**, 228-236. doi: 10.1152/physiolgenomics.00149.2012

Chandley, M.J., Szebeni, A., Szebeni, K., Crawford, J.D., Stockmeier, C.A., Turecki, G. et al. (2014) Elevated gene expression of glutamate receptors in noradrenergic neurons from the locus coeruleus in major depression. *Int J Neuropsychopharmacol*. **17**, 1569-1578. doi: 10.1017/S1461145714000662

Supplementary Material

Chauhan, P.S., Misra, U.K., Kalita, J., Chandravanshi, L.P., and Khanna, V.K. (2016) Memory and learning seems to be related to cholinergic dysfunction in the JE rat model. *Physiol Behav*. **156**, 148-555. doi: 10.1016/j.physbeh.2016.01.006

Chen, C., Yang, J.M., Hu, T.T., Xu, T.J., Xu, W.P., and Wei, W. (2013) Elevated dopamine D2 receptor in prefrontal cortex of CUMS rats is associated with downregulated cAMP-independent signaling pathway. *Can J Physiol Pharmacol*. **91**, 750-758. doi: 10.1139/cjpp-2012-0399

Claustre, Y., Leonetti, M., Santucci, V., Bougault, I., Desvignes, C., Rouquier, L. et al. (2008) Effects of the beta3-adrenoceptor (Adrb3) agonist SR58611A (amibegron) on serotonergic and noradrenergic transmission in the rodent: relevance to its antidepressant/anxiolytic-like profile. *Neuroscience*. **156**, 353-364. doi: 10.1016/j.neuroscience.2008.07.011

Cryan, J.F., Kelly, P.H., Neijt, H.C., Sansig, G., Flor, P.J., and van Der Putten, H. (2003) Antidepressant and anxiolytic-like effects in mice lacking the group III metabotropic glutamate receptor mGluR7. *Eur J Neurosci*. **17**, 2409-2417. doi: 10.1046/j.1460-9568.2003.02667.x

Davidson, S., Golden, J.P., Copits, B.A., Ray, P.R., Vogt, S.K., Valtcheva, M.V. et al. (2016) Group II mGluRs suppress hyperexcitability in mouse and human nociceptors. *Pain*. **157**, 2081-2088. doi: 10.1097/j.pain.0000000000000621

dela Pena, I., Bang, M., Lee, J., de la Pena, J.B., Kim, B.N., Han, D.H. et al. (2015) Common prefrontal cortical gene expression profiles between adolescent SHR/NCrl and WKY/NCrl rats which showed inattention behavior. *Behav Brain Res*. **291**, 268-276. doi: 10.1016/j.bbr.2015.05.012

Desbonnet, L., Tighe, O., Karayiorgou, M., Gogos, J.A., Waddington, J.L., and O'Tuathaigh, C.M. (2012) Physiological and behavioural responsivity to stress and anxiogenic stimuli in COMT-deficient mice. *Behav Brain Res*. **228**, 351-358. doi: 10.1016/j.bbr.2011.12.014

Descalzi, G., Chen, T., Koga, K., Li, X.Y., Yamada, K., and Zhuo, M. (2013) Cortical GluK1 kainate receptors modulate scratching in adult mice. *J Neurochem*. **126**, 636-650. doi: 10.1111/jnc.12351

Doze, V.A., Handel, E.M., Jensen, K.A., Darsie, B., Luger, E.J., Haselton, J.R. et al. (2009) alpha(1A)- and alpha(1B)-adrenergic receptors differentially modulate antidepressant-like behavior in the mouse. *Brain Res*. **1285**, 148-157. doi: 10.1016/j.brainres.2009.06.035

Ellingrod, V.L., Perry, P.J., Ringold, J.C., Lund, B.C., Bever-Stille, K., Fleming, F. et al. (2005) Weight gain associated with the -759C/T polymorphism of the 5HT2C receptor and olanzapine. *Am. J. Med. Genet. B Neuropsychiatr. Genet.* **134B**, 76--78. doi 10.1002/ajmg.b.20169

Farago, N., Kocsis, A.K., Brasko, C., Lovas, S., Rozsa, M., Baka, J., et al. (2016) Human neuronal changes in brain edema and increased intracranial pressure. *Acta Neuropathol Commun*. **4**: 78. doi: 10.1186/s40478-016-0356-x

Fatemi, S.H., Folsom, T.D., and Thuras, P.D. (2011) Deficits in GABA(B) receptor system in schizophrenia and mood disorders: a postmortem study. *Schizophr Res*. **128**, 37-43. doi: 10.1016/j.schres.2010.12.025Freund, N., Thompson, B.S., Sonntag, K., Meda, S., and Andersen, S.L. (2016) When the party is over: depressive-like states in rats following termination of cortical D1 receptor overexpression. *Psychopharmacology (Berl)*. **233**, 1191-1201. doi: 10.1007/s00213-015-4200-y

Fatemi, S.H., Folsom, T.D., Rooney, R.J., and Thuras, P.D. (2013) Expression of GABAA α2-, β1- and ε-receptors are altered significantly in the lateral cerebellum of subjects with schizophrenia, major depression and bipolar disorder. *Transl Psychiatry*. **3**: e303. doi: 10.1038/tp.2013.64

Supplementary Material

Fendt, M., Imobersteg, S., Peterlik, D., Chaperon, F., Mattes, C., Wittmann, C. et al. (2013) Differential roles of mGlu(7) and mGlu(8) in amygdala-dependent behavior and physiology. *Neuropharmacology*. **72**, 215-223. doi: 10.1016/j.neuropharm.2013.04.052

Fujioka, R., Nii, T., Iwaki, A., Shibata, A., Ito, I., Kitaichi, K., Nomura, M. et al. (2014) Comprehensive behavioral study of mGluR3 knockout mice: implication in schizophrenia related endophenotypes. *Mol Brain*. **7**: 31. doi: 10.1186/1756-6606-7-31

Garzon, M., and Pickel, V.M. (2013) Somatodendritic targeting of M5 muscarinic receptor in the rat ventral tegmental area: implications for mesolimbic dopamine transmission. *J Comp Neurol*. **521**, 2927-2946. doi: 10.1002/cne.23323

Gibbons, A.S., Jeon, W.J., Scarr, E., and Dean, B. (2016) Changes in muscarinic M2 receptor levels in the cortex of subjects with bipolar disorder and major depressive disorder and in rats after treatment with mood stabilisers and antidepressants. *Int J Neuropsychopharmacol*. **19**: pyv118. doi: 10.1093/ijnp/pyv118

Girven, M., Dugdale, H.F., Owens, D.J., Hughes, D.C., Stewart, C.E., and Sharples, A.P. (2016) l-Glutamine improves skeletal muscle cell differentiation and prevents myotube atrophy after cytokine (TNF-α) stress via reduced p38 mapk signal transduction. *J Cell Physiol*. **231**, 2720-2732. doi: 10.1002/jcp.25380

Goswami, D.B., Jernigan, C.S., Chandran, A., Iyo, A.H., May, W.L., Austin, M.C., Stockmeier, C.A., and Karolewicz, B. (2013) Gene expression analysis of novel genes in the prefrontal cortex of major depressive disorder subjects. *Prog Neuropsychopharmacol Biol Psychiatry*. **43**, 126-133. doi: 10.1016/j.pnpbp.2012.12.010

Graham, D.L., Durai, H.H., Garden, J.D., Cohen, E.L., Echevarria, F.D., and Stanwood, G.D. (2015) Loss of dopamine D2 receptors increases parvalbumin-positive interneurons in the anterior cingulate cortex. *ACS Chem Neurosci*. **6**, 297-305. doi: 10.1021/cn500235m

Granados-Soto, V., Argüelles, C.F., Rocha-González, H.I., Godínez-Chaparro, B., Flores-Murrieta, F.J., and Villalon, C.M. (2010) The role of peripheral 5-HT1A, 5-HT1B, 5-HT1D, 5-HT1E and 5-HT1F serotonergic receptors in the reduction of nociception in rats. *Neuroscience*. **165**, 561-568. doi: 10.1016/j.neuroscience.2009.10.020

Gray, A.L., Hyde, T.M., Deep-Soboslay, A., Kleinman, J.E., and Sodhi, M.S. (2015) Sex differences in glutamate receptor gene expression in major depression and suicide. *Mol Psychiatry*. **20**, 1057-1068. doi: 10.1038/mp.2015.91

Guo, Y., Zhang, H., Gao, J., Wei, S., Song, C., Sun, P., and Qiao, M. (2015) Study of genes associated with the 'anger-in' and 'anger-out' emotions of humans using a rat model. *Exp Ther Med*. **9**, 1448-1454. doi: 10.3892/etm.2015.2246

Han, S., Yang, S.H., Kim, J.Y., Mo, S., Yang, E., Song, K.M. et al. (2017) Down-regulation of cholinergic signaling in the habenula induces anhedonia-like behavior. *Sci Rep*. **7**: 900. doi: 10.1038/s41598-017-01088-6

Hanack, C., Moroni, M., Lima, W.C., Wende, H., Kirchner, M., Adelfinger, L., et al. (2015) GABA blocks pathological but not acute TRPV1 pain signals. *Cell*. **160**, 759-770. doi: 10.1016/j.cell.2015.01.022

He, Z., Sun, X., Guo, Z., and Zhang, J.H. (2011) Expression and role of COMT in a rat subarachnoid hemorrhage model. *Acta Neurochir Suppl*. **110**, 181-187. doi: 10.1007/978-3-7091-0353-1_32

Heskin-Sweezie, R., Titley, H.K., Baizer, J.S., and Broussard, D.M. (2010) Type B GABA receptors contribute to the restoration of balance during vestibular compensation in mice. *Neuroscience*. **169**, 302-314. doi: 10.1016/j.neuroscience.2010.04.008

Hillman, B.G., Gupta, S.C., Stairs, D.J., Buonanno, A., and Dravid, S.M. (2011) Behavioral analysis of NR2C knockout mouse reveals deficit in acquisition of conditioned fear and working memory. *Neurobiol Learn Mem*. **95**, 404-414. doi: 10.1016/j.nlm.2011.01.008

Supplementary Material

Hoffmann, K., Muller, J.S., Stricker, S., Megarbane, A., Rajab, A., Lindner, T.H. et al. (2006) Escobar syndrome is a prenatal myasthenia caused by disruption of the acetylcholine receptor fetal gamma subunit. *Am J Hum Genet*. **79**, 303-312. doi: 10.1086/506257

Holehonnur, R., Phensy, A.J., Kim, L.J., Milivojevic, M., Vuong, D., Daison, D.K. et al. (2016) Increasing the GluN2A/GluN2B ratio in neurons of the mouse basal and lateral amygdala inhibits the modification of an existing fear memory trace. *J Neurosci*. **36,** 9490-9504. doi: 10.1523/jneurosci.1743-16.2016

Holmes, A., Murphy, D.L., and Crawley, J.N. (2002) Reduced aggression in mice lacking the serotonin transporter. *Psychopharmacology (Berl)*. **161**, 160-167. doi: 10.1007/s00213-002-1024-3

Hu, L., Han, B., Zhao, X., Mi, L., Song, Q., Wang, J. et al. (2016) Chronic early postnatal scream sound stress induces learning deficits and NMDA receptor changes in the hippocampus of adult mice. *Neuroreport*. **27**, 397-403. doi: 10.1097/wnr.0000000000000552

Ikeda, E., Matsunaga, N., Kakimoto, K., Hamamura, K., Hayashi, A., Koyanagi, S., and Ohdo, S. (2013) Molecular mechanism regulating 24-hour rhythm of dopamine D3 receptor expression in mouse ventral striatum. *Mol Pharmacol*. **83**, 959-967. doi: 10.1124/mol.112.083535

Innos, J., Philips, M.A., Leidmaa, E., Heinla, I., Raud, S., Reemann, P. et al. (2011) Lower anxiety and a decrease in agonistic behaviour in Lsamp-deficient mice. *Behav Brain Res*. **217**, 21-31. doi: 10.1016/j.bbr.2010.09.019

Inoue, T., Izumi, T., Maki, Y., Muraki, I., and Koyama, T. (2000) Effect of the dopamine D(1/5) antagonist SCH 23390 on the acquisition of conditioned fear. *Pharmacol Biochem Behav*. **66**, 573-578. doi: 10.1016/S0091-3057(00)00254-9

Irnaten, M., Walwyn, W.M., Wang, J., Venkatesan, P., Evans, C., Chang, K.S. et al. (2002) Pentobarbital enhances GABAergic neurotransmission to cardiac parasympathetic neurons, which is prevented by expression of GABA(A) epsilon subunit. *Anesthesiology*. **97**, 717-724.

Jamart, C., Naslain, D., Gilson, H., and Francaux, M. (2013) Higher activation of autophagy in skeletal muscle of mice during endurance exercise in the fasted state. *Am J Physiol Endocrinol Metab*. **305**, E964-E974. doi: 10.1152/ajpendo.00270.2013

Kaneko, T., Kaneko, M., Chokechanachaisakul, U., Kawamura, J., Kaneko, R., Sunakawa, M. et al. (2010) Artificial dental pulp exposure injury up-regulates antigen-presenting cell-related molecules in rat central nervous system. *J Endod*. **36**, 459-464. doi: 10.1016/j.joen.2009.12.011

Kawai, T., Morita, K., Masuda, K., Nishida, K., Sekiyama, A., Teshima-Kondo, S. et al. (2007) Physical exercise-associated gene expression signatures in peripheral blood. *Clin J Sport Med*. **17**, 375-383. doi: 10.1097/JSM.0b013e31814c3e4f

Keck, T.M., Suchland, K.L., Jimenez, C.C., and Grandy, D.K. (2013) Dopamine D4 receptor deficiency in mice alters behavioral responses to anxiogenic stimuli and the psychostimulant methylphenidate. *Pharmacol Biochem Behav*. **103**, 831-841. doi: 10.1016/j.pbb.2012.12.006

Kim, H., Kim, Y., Bae, S., Lim, S.H., Jang, M., Choi, J. et al. (2015) Vitamin C deficiency causes severe defects in the development of the neonatal cerebellum and in the motor behaviors of Gulo(-/-) mice. *Antioxid Redox Signal*. **23**, 1270-1283. doi: 10.1089/ars.2014.6043

Kolber, B.J., Montana, M.C., Carrasquillo, Y., Xu, J., Heinemann, S.F., Muglia, L.J., and Gereau, R.W. 4th. (2010) Activation of metabotropic glutamate receptor 5 in the amygdala modulates pain-like behavior. *J Neurosci*. **30**, 8203-8213. doi: 10.1523/jneurosci.1216-10.2010

Supplementary Material

Kosmowska, B., Wardas, J., Głowacka, U., Ananthan, S., and Ossowska, K. (2016) Pramipexole at a low dose induces beneficial effect in the harmaline-induced model of essential tremor in rats. *CNS Neurosci Ther*. **22**, 53-62. doi: 10.1111/cns.12467

Kratsman, N., Getselter, D., and Elliott, E. (2016) Sodium butyrate attenuates social behavior deficits and modifies the transcription of inhibitory/excitatory genes in the frontal cortex of an autism model. *Neuropharmacology*. **102**, 136-145. doi: 10.1016/j.neuropharm.2015.11.003

Kudryavtseva, N.N., Smagin, D.A., Kovalenko, I.L., Galyamina, A.G., Vishnivetskaya, G.B., Babenko, V.N., and Orlov Y.L. (2017) Serotonergic genes in the development of anxiety/depression-like state and pathology of aggressive behavior in male mice: RNA-seq data. *Mol Biol (Mosk).* **51**, 288-300. doi: 10.1134/S0026893317020133

Kumaran, D., Udayabanu, M., Nair, R.U., R, A., and Katyal, A. (2008) Benzamide protects delayed neuronal death and behavioural impairment in a mouse model of global cerebral ischemia. *Behav Brain Res.* **192**,178-184. doi: 10.1016/j.bbr.2008.03.043

Lainez, S., Valente, P., Ontoria-Oviedo, I., Estevez-Herrera, J., Camprubí-Robles, M., Ferrer-Montiel, A., and Planells-Cases, R. (2010) GABAA receptor associated protein (GABARAP) modulates TRPV1 expression and channel function and desensitization. *FASEB J*. **24**, 1958-1970. doi: 10.1096/fj.09-151472

Landrum, M.J., Lee, J.M., Riley, G.R., Jang, W., Rubinstein, W.S., Church, D.M., et al. (2014). ClinVar: public archive of relationships among sequence variation and human phenotype. *Nucleic Acids Res*. 42, D980-D985. doi:10.1093/nar/gkt1113

Lax, N.C., George, D.C., Ignatz, C., and Kolber, B.J. (2014) The mGluR5 antagonist fenobam induces analgesic conditioned place preference in mice with spared nerve injury. *PLoS One*. **9**: e103524. doi: 10.1371/journal.pone.0103524

Lefebvre, J.L., Ono, F., Puglielli, C., Seidner, G., Franzini-Armstrong, C., Brehm, P., and Granato, M. (2004) Increased neuromuscular activity causes axonal defects and muscular degeneration. *Development*. **131**, 2605-2618. doi: 10.1242/dev.01123

Leurquin-Sterk, G., Van den Stock, J., Crunelle, C.L., de Laat, B., Weerasekera, A., Himmelreich, U. et al. (2016) Positive association between limbic metabotropic glutamate receptor 5 availability and novelty-seeking temperament in humans: an 18F-FPEB PET Study. *J Nucl Med*. **57**, 1746-1752. doi: 10.2967/jnumed.116.176032

Li, M.D., Wang, J., Niu, T., Ma, J.Z., Seneviratne, C., Ait-Daoud, N. et al. (2014) Transcriptome profiling and pathway analysis of genes expressed differentially in participants with or without a positive response to topiramate treatment for methamphetamine addiction. *BMC Med Genomics*. **7**: 65. doi: 10.1186/s12920-014-0065-x

Lin, H.C., Mao, S.C., and Gean, P.W. (2009) Block of gamma-aminobutyric acid-A receptor insertion in the amygdala impairs extinction of conditioned fear. *Biol Psychiatry*. **66**, 665-673. doi: 10.1016/j.biopsych.2009.04.003

Little, J.W., Ford, A., Symons-Liguori, A.M., Chen, Z., Janes, K., and Doyle, T. et al. (2015) Endogenous adenosine A3 receptor activation selectively alleviates persistent pain states. *Brain*. **138**, 28-35. doi: 10.1093/brain/awu330

Liu, M., Fitzgibbon, M., Wang, Y., Reilly, J., Qian, X., O'Brien, T. et al. (2018) Ulk4 regulates GABAergic signaling and anxiety-related behavior. *Transl Psychiatry*. **8**, 43. doi: 10.1038/s41398-017-0091-5

Ma, X., Bao, W., Wang, X., Wang, Z., Liu, Q., Yao, Z. et al. (2014) Role of spinal GABAA receptor reduction induced by stress in rat thermal hyperalgesia. *Exp Brain Res*. **232**, 3413-3420. doi: 10.1007/s00221-014-4027-5

Supplementary Material

Maclennan, C.A., Vincent, A., Marx, A., Willcox, N., Gilhus, N.E., Newsom-Davis, J., and Beeson, D. (2008) Preferential expression of AChR epsilon-subunit in thymomas from patients with myasthenia gravis. *J Neuroimmunol*. **201-202**, 28-32. doi: 10.1016/j.jneuroim.2008.06.016

Malek, N., Kostrzewa, M., Makuch, W., Pajak, A., Kucharczyk, M., Piscitelli, F. et al. (2016) The multiplicity of spinal AA-5-HT anti-nociceptive action in a rat model of neuropathic pain. *Pharmacol Res*. **111**, 251-263. doi: 10.1016/j.phrs.2016.06.012

Mann, P.E. (2014) Gene expression profiling during pregnancy in rat brain tissue. *Brain Sci*. **4**, 125-135. doi: 10.3390/brainsci4010125

Manohar, S., Dahar, K., Adler, H.J., Dalian, D., and Salvi, R. (2016) Noise-induced hearing loss: Neuropathic pain via Ntrk1 signaling. *Mol Cell Neurosci*. **75**, 101-112. doi: 10.1016/j.mcn.2016.07.005

Manzano, R., Toivonen, J.M., Olivan, S., Calvo, A.C., Moreno-Igoa, M., Munoz, M.J. et al. (2011) Altered expression of myogenic regulatory factors in the mouse model of amyotrophic lateral sclerosis. *Neurodegener Dis*. **8,** 386-396. doi: 10.1159/000324159

Mead, A.N., Morris, H.V., Dixon, C.I., Rulten, S.L., Mayne, L.V., Zamanillo, D., and Stephens, D.N. (2006) AMPA receptor GluR2, but not GluR1, subunit deletion impairs emotional response conditioning in mice. *Behav Neurosci*. **120**, 241-248. doi: 10.1016/j.nlm.2016.07.005

Meunier, C.N.J., Cancela, J.M., and Fossier, P. (2017) Lack of GSK3β activation and modulation of synaptic plasticity by dopamine in 5-HT1A-receptor KO mice. *Neuropharmacology*. **113**, 124-136. doi: 10.1016/j.neuropharm.2016.09.025

Michalovicz, L.T., and Konat, G.W. (2014) Peripherally restricted acute phase response to a viral mimic alters hippocampal gene expression. *Metab Brain Dis*. 29, 75-86. doi: 10.1007/s11011-013-9471-6

Mohammadi, S.A., and Christie, M.J. (2015) Conotoxin interactions with α9α10-nachrs: is the α9α10-nicotinic acetylcholine receptor an important therapeutic target for pain management? *Toxins (Basel)*. **7**, 3916-3932. doi: 10.3390/toxins7103916

Mulugeta, E., El-Bakri, N., Karlsson, E., Elhassan, A., and Adem, A. (2003) Loss of muscarinic M4 receptors in spinal cord of arthritic rats: implications for a role of M4 receptors in pain response. *Brain Res*. **982**, 284-287. doi: 10.1016/S0006-8993(03)03025-7

Naderi, N., Majidi, M., Mousavi, Z., Khoramian Tusi, S., Mansouri, Z., and Khodagholi, F. (2012) The interaction between intrathecal administration of low doses of palmitoylethanolamide and AM251 in formalin-induced pain related behavior and spinal cord IL1-β expression in rats. *Neurochem Res*. **37**, 778-785. doi: 10.1007/s11064-011-0672-2

Niemann, S., Kanki, H., Fukui, Y., Takao, K., Fukaya, M., Hynynen, M.N. et al. (2007) Genetic ablation of NMDA receptor subunit NR3B in mouse reveals motoneuronal and nonmotoneuronal phenotypes. *Eur J Neurosci*. **26,** 1407-1420. doi: 10.1111/j.1460-9568.2007.05774.x

Niesler, B., Walstab, J., Combrink, S., Moller, D., Kapeller, J., et al. (2007) Characterization of the novel human serotonin receptor subunits 5-HT3C,5-HT3D, and 5-HT3E. *Mol Pharmacol*. **72**, 8-17. doi: 10.1124/mol.106.032144

Obradovic, A.L., Scarpa, J., Osuru, H.P., Weaver, J.L., Park, J.Y., et al. (2015) Silencing the α2 subunit of γ-aminobutyric acid type A receptors in rat dorsal root ganglia reveals its major role in antinociception posttraumatic nerve injury. *Anesthesiology*. **123**, 654-667. doi: 10.1097/ALN.0000000000000767

Supplementary Material

Overton, H.A., Babbs, A.J., Doel, S.M., Fyfe, M.C., Gardner, L.S., Griffin, G. et al. (2006) Deorphanization of a G protein-coupled receptor for oleoylethanolamide and its use in the discovery of small-molecule hypophagic agents. *Cell Metab*. **3**, 167-175. doi: 10.1016/j.cmet.2006.02.004

Pienimaeki-Roemer, A., Konovalova, T., Musri, M.M., Sigruener, A., Boettcher, A., Meister, G., and Schmitz, G. (2017) Transcriptomic profiling of platelet senescence and platelet extracellular vesicles. *Transfusion*. **57**, 144-156. doi: 10.1111/trf.13896

Ponnazhagan, R., Harms, A.S., Thome, A.D., Jurkuvenaite, A., Gogliotti, R., Niswender, C.M. et al. (2016) The metabotropic glutamate receptor 4 positive allosteric modulator ADX88178 inhibits inflammatory responses in primary microglia. *J Neuroimmune Pharmacol*. **11**, 231-237. doi: 10.1007/s11481-016-9655-z

Popova, N.K. (2006) From genes to aggressive behavior: the role of serotonergic system. *Bioessays*.;**28**, 495-503. doi: 10.1002/bies.20412

Popova, N.K., Naumenko, V.S., Kozhemyakina, R.V., and Plyusnina, I.Z. (2010) Functional characteristics of serotonin 5-HT2A and 5-HT2C receptors in the brain and the expression of the 5-HT2A and 5-HT2C receptor genes in aggressive and non-aggressive rats. *Neurosci. Behav. Physiol.* **40**, 357--361. doi 10.1007/s11055-010-9264-x

Rabenstein, R.L., Caldarone, B.J., and Picciotto, M.R. (2006) The nicotinic antagonist mecamylamine has antidepressant-like effects in wild-type but not beta2- or alpha7-nicotinic acetylcholine receptor subunit knockout mice. *Psychopharmacology (Berl)*. **189**, 395-401. doi: 10.1007/s00213-006-0568-z

Rahimi, A., Hajizadeh Moghaddam, A., and Roohbakhsh, A. (2015) Central administration of GPR55 receptor agonist and antagonist modulates anxiety-related behaviors in rats. *Fundam Clin Pharmacol*. **29**, 185-190. doi: 10.1111/fcp.12099

Reif, A., Jacob, C.P., Rujescu, D., Herterich, S., Lang, S., Gutknecht, L. et al. (2009) Influence of functional variant of neuronal nitric oxide synthase on impulsive behaviors in humans. *Arch Gen Psychiatry*. **66**, 41-50. doi: 10.1001/archgenpsychiatry.2008.510

Ren, Z., Sahir, N., Murakami, S., Luellen, B.A., Earnheart, J.C., Lal R. et al. (2015) Defects in dendrite and spine maturation and synaptogenesis associated with an anxious-depressive-like phenotype of GABAA receptor-deficient mice. *Neuropharmacology.* **88**, 171-179. doi: 10.1016/j.neuropharm.2014.07.019

Richard, J.M., and Berridge, K.C. (2011) Metabotropic glutamate receptor blockade in nucleus accumbens shell shifts affective valence towards fear and disgust. *Eur J Neurosci*. **33**, 736-747. doi: 10.1111/j.1460-9568.2010.07553.x

Roh, D.H., Seo, H.S., Yoon, S.Y., Song, S., Han, H.J., Beitz, A.J., and Lee JH. (2010) Activation of spinal alpha-2 adrenoceptors, but not mu-opioid receptors, reduces the intrathecal N-methyl-D-aspartate-induced increase in spinal NR1 subunit phosphorylation and nociceptive behaviors in the rat. *Anesth Analg*. **110**, 622-629. doi: 10.1213/ANE.0b013e3181c8afc1

Rossi, F., Marabese, I., De Chiaro, M., Boccella, S., Luongo, L., Guida, F. et al. (2014) Dorsal striatum metabotropic glutamate receptor 8 affects nocifensive responses and rostral ventromedial medulla cell activity in neuropathic pain conditions. *J Neurophysiol*. **111**, 2196-2209. doi: 10.1152/jn.00212.2013

Sagata, N., Iwaki, A., Aramaki, T., Takao, K., Kura, S., Tsuzuki, T. et al. (2010) Comprehensive behavioural study of GluR4 knockout mice: implication in cognitive function. *Genes Brain Behav*. **9**, 899-909. doi: 10.1111/j.1601-183X.2010.00629.x

Sadeghi, H., and Taylor, H.S. (2010) HOXA10 regulates endometrial GABAA {pi} receptor expression and membrane translocation. *Am J Physiol Endocrinol Metab*. **298**, e889-e893. doi: 10.1152/ajpendo.00577.2009

Supplementary Material

Sakata, K., and Overacre, A.E. (2017) Promoter IV-BDNF deficiency disturbs cholinergic gene expression of CHRNA5, CHRM2, and CHRM5: effects of drug and environmental treatments. *J Neurochem*. **143**, 49-64. doi: 10.1111/jnc.14129

Schneider, M., Kasanetz, F., Lynch, D.L., Friemel, C.M., Lassalle, O., Hurst, D.P. et al. (2015) Enhanced functional activity of the cannabinoid type-1 receptor mediates adolescent behavior. *J Neurosci*. **35**, 13975-13988. doi: 10.1523/jneurosci.1937-15.2015

Serchov, T., Clement, H.W., Schwarz, M.K., Iasevoli, F., Tosh, D.K., Idzko, M. et al. (2015) Increased signaling via adenosine a1 receptors, sleep deprivation, imipramine, and ketamine inhibit depressive-like behavior via induction of Homer1a. *Neuron*. **87**, 549-562. doi: 10.1016/j.neuron.2015.07.010

Shaltiel, G., Maeng, S., Malkesman, O., Pearson, B., Schloesser, R.J., Tragon, T. et al. (2008) Evidence for the involvement of the kainate receptor subunit GluR6 (GRIK2) in mediating behavioral displays related to behavioral symptoms of mania. *Mol Psychiatry*. **13**, 858-872. doi: 10.1038/mp.2008.20

Sherry, S.T., Ward, M.H., Kholodov, M., Baker, J., Phan, L., Smigielski, E.M., et al. (2001). dbSNP: the NCBI database of genetic variation. *Nucleic Acids Res*. **29**, 308–311. doi:10.1093/nar/29.1.308

Soggiu, A., Piras, C., Greco, V., Devoto, P., Urbani, A., Calzetta, L. et al. (2016) Exploring the neural mechanisms of finasteride: a proteomic analysis in the nucleus accumbens. *Psychoneuroendocrinology*. **74**, 387-396. doi: 10.1016/j.psyneuen.2016.10.001

Spitzmaul, G., Gumilar, F., Dilger, J.P., and Bouzat, C. (2009) The local anaesthetics proadifen and adiphenine inhibit nicotinic receptors by different molecular mechanisms. *Br J Pharmacol*. **157**, 804-817. doi: 10.1111/j.1476-5381.2009.00214.x.

Stojkov, N.J., Janjic, M.M., Baburski, A.Z., Mihajlovic, A.I., Drljaca, D.M., Sokanovic, S.J. et al. (2013) Sustained in vivo blockade of α_1-adrenergic receptors prevented some of stress-triggered effects on steroidogenic machinery in Leydig cells. *Am J Physiol Endocrinol Metab*. **305**, E194-E204. doi: 10.1152/ajpendo.00100.2013

Sullivan, G.W., Fang, G., Linden, J., and Scheld, W.M. (2004) A2A adenosine receptor activation improves survival in mouse models of endotoxemia and sepsis. *J Infect Dis*. **189**, 1897-1904. doi: 10.1086/386311

Tadavarty, R., Hwang, J., Rajput, P.S., Soja, P.J., Kumar, U., and Sastry, B.R. (2015) Are presynaptic GABA-Cρ2 receptors involved in anti-nociception? *Neurosci Lett*. **606**, 145-150. doi: 10.1016/j.neulet.2015.08.049

Tanaka, S., Kuratsune, H., Hidaka, Y., Hakariya, Y., Tatsumi, K.I., Takano, T. et al. (2003) Autoantibodies against muscarinic cholinergic receptor in chronic fatigue syndrome. *Int J Mol Med*. **12**, 225-230. doi: 10.3892/ijmm.12.2.225

Thomson, S.R., Seo, S.S., Barnes, S.A., Louros, S.R., Muscas, M., Dando, O. et al. (2017) Cell-type-specific translation profiling reveals a novel strategy for treating fragile X syndrome. *Neuron*. **95**, 550-563. doi: 10.1016/j.neuron.2017.07.013

Tunc-Ozcan, E., Ullmann, T.M., Shukla, P.K., and Redei, E.E. (2013) Low-dose thyroxine attenuates autism-associated adverse effects of fetal alcohol in male offspring's social behavior and hippocampal gene expression. *Alcohol Clin Exp Res*. **37**, 1986-1995. doi: 10.1111/acer.12183

Uteshev, V.V. (2012) α7 nicotinic ACh receptors as a ligand-gated source of Ca(2+) ions: the search for a Ca(2+) optimum. *Adv Exp Med Biol*. **740**, 603-638. doi: 10.1007/978-94-007-2888-2_27

van der Kooij, M.A., Grosse, J., Zanoletti, O., Papilloud, A., and Sandi, C. (2015) The effects of stress during early postnatal periods on behavior and hippocampal neuroplasticity markers in adult male mice. *Neuroscience*. **311**, 508-518. doi: 10.1016/j.neuroscience.2015.10.058

Supplementary Material

Vinals, X., Molas, S., Gallego, X., Fernandez-Montes, R.D., Robledo, P., Dierssen, M., and Maldonado, R. (2012) Overexpression of α3/α5/β4 nicotinic receptor subunits modifies impulsive-like behavior. *Drug Alcohol Depend*. **122**, 247-252. doi: 10.1016/j.drugalcdep.2011.09.027

Vincler, M.A., and Eisenach, J.C. (2005) Knock down of the alpha 5 nicotinic acetylcholine receptor in spinal nerve-ligated rats alleviates mechanical allodynia. *Pharmacol Biochem Behav*. **80,** 135-143. doi: 10.1016/j.pbb.2004.10.011

Vincler, M., Wittenauer, S., Parker, R., Ellison, M., Olivera, B.M., and McIntosh, J.M. (2006) Molecular mechanism for analgesia involving specific antagonism of alpha9alpha10 nicotinic acetylcholine receptors. *Proc Natl Acad Sci USA*. **103**, 17880-17884. doi: 10.1073/pnas.0608715103

Walker, A.G., Wenthur, C.J., Xiang, Z., Rook, J.M., Emmitte, K.A., Niswender, C.M. et al. (2015) Metabotropic glutamate receptor 3 activation is required for long-term depression in medial prefrontal cortex and fear extinction. *Proc Natl Acad Sci USA*. **112**, 1196-1201. doi: 10.1073/pnas.1416196112

Wilhelm, C.J., Choi, D., Huckans, M., Manthe, L., and Loftis, J.M. (2013) Adipocytokine signaling is altered in Flinders sensitive line rats, and adiponectin correlates in humans with some symptoms of depression. *Pharmacol Biochem Behav*. **103**, 643-651. doi: 10.1016/j.pbb.2012.11.001

Wu, W.P., Hao, J.X., Halldner, L., Lovdahl, C., DeLander, G.E., Wiesenfeld-Hallin, Z. et al. (2005) Increased nociceptive response in mice lacking the adenosine A1 receptor. *Pain*. **113**, 395-404. doi: 10.1016/j.pain.2004.11.020

Xu, Y., Wang, J., Rao, S., Ritter, M., Manor, L.C., Backer, R. et al. (2017a) An integrative computational approach to evaluate genetic markers for bipolar disorder. *Sci Rep*. **7**: 6745. doi: 10.1038/s41598-017-05846-4

Yalcin, I., Choucair-Jaafar, N., Benbouzid, M., Tessier, L.H., Muller, A., Hein, L. et al. (2009) beta(2)-Adrenoceptors are critical for antidepressant treatment of neuropathic pain. *Ann Neurol*. **65**, 218-225. doi: 10.1002/ana.21542

Yang, L., Xu, T., Zhang, K., Wei, Z., Li, X., Huang, M. et al. (2016) The essential role of hippocampal alpha6 subunit-containing GABAA receptors in maternal separation stress-induced adolescent depressive behaviors. *Behav Brain Res*. **313**, 135-143. doi: 10.1016/j.bbr.2016.07.002

Young, J.W., van Enkhuizen, J., Winstanley, C.A., and Geyer, M.A. (2011) Increased risk-taking behavior in dopamine transporter knockdown mice: further support for a mouse model of mania. *J Psychopharmacol*. **25**, 934-943. doi: 10.1177/0269881111400646

Zhang, W.G., Zhang, L.C., Peng, Z.D., and Zeng, Y.M. (2009) Intrathecal injection of GluR6 antisense oligodeoxynucleotides alleviates acute inflammatory pain of rectum in rats. *Neurosci Bull*. 25, 319-**323**. doi: 10.1007/s12264-009-0326-4

Zheng, W., Xie, W., Zhang, J., Strong, J.A., Wang, L., Yu, L. et al. (2003) Function of gamma-aminobutyric acid receptor/channel rho 1 subunits in spinal cord. *J Biol Chem*. **278**, 48321-48329. doi: 10.1074/jbc.M307930200

Zhou, R., Yuan, P., Wang, Y., Hunsberger, J.G., Elkahloun, A., Wei, Y. et al. (2009) Evidence for selective microRNAs and their effectors as common long-term targets for the actions of mood stabilizers. *Neuropsychopharmacology*. **34**, 1395-405. doi: 10.1038/npp.2008.131

Zuo, G., Zhang, D., Mu, R., Shen, H., Li, X., Wang, Z. et al. (2018) Resolvin D2 protects against cerebral ischemia/reperfusion injury in rats. *Mol Brain*. **11**: 9. doi: 10.1186/s13041-018-0351-1

Zurek, A.A., Kemp, S.W., Aga, Z., Walker, S., Milenkovic, M., Ramsey, A.J. et al. (2016) α5GABAA receptor deficiency causes autism-like behaviors. *Ann Clin Transl Neurol*. **3**, 392-398. doi: 10.1002/acn3.303
