# Supplementary material for: Natural Selection Equally Supports the Human Tendencies in Subordination and Domination: A Genome-Wide Study With in silico Confirmation and in vivo Validation in Mice
Source: Front Genet. 2019 Feb 20;10:73. doi: 10.3389/fgene.2019.00073 (PMC6404730; doi:10.3389/fgene.2019.00073)
Supplement: Supplementary file 3 [file Data_Sheet_3.PDF]

Supplementary Material

Natural selection equally supports the human tendencies in subordination and domination: a genome-wide study with *in silico* confirmation and *in vivo* validation in mice

Irina Chadaeva, Petr Ponomarenko, Dmitry Rasskazov, Ekaterina Sharypova, Elena Kashina, Maxim Kleshchev, Mikhail Ponomarenko*, Vladimir Naumenko, Ludmila Savinkova, Nikolay Kolchanov, Ludmila Osadchuk, Alexandr Osadchuk

***Correspondence:** Mikhail Ponomarenko (pon@bionet.nsc.ru)

Table S3. Candidate SNP markers predicted in this work near TBP-binding sites in the promoter of the human genes encoding neurotrophinergic-system–related proteins (e.g., growth factors, receptors, enzymes)

Gene, OMIM	dbSNP (Sherry et al., 2001)	5′ flank	wt	**mut**	3′ flank	K_D, nM, prediction						**Known physiological or** candidate SNP[$] markers	Ss	**ClinVar** or Reference
						wt	**mut**	Δ	Z	α	ρ			
ADCYAP1, 102980	rs772551357	catcttttta	*g*	**a**	ttcccccatt	*70*	**39**	>	10	10^{-6}	A	hypersensitivity to chronic neuropathic pain	↓	Missig et al., 2017
	rs181086670	gcaaacgagt	*c*	**g**[*)]	ccgcagctcc	*70*	**55**	>	4	10^{-3}	B		↓	
	rs532841894	ggagttgaag	*g*	**t**	gtaagggagg	*21*	**11**	>	9	10^{-6}	A		↓	
	rs779405150	gcctcggcaa	*a*	**g**	cgagtcccgc	*70*	**117**	<	11	10^{-6}	A	increased stress-induced depression-like behaviors	↓	Lehmann et al., 2013
ADCYAP1R1, 102981	rs914413371	gccgcgcagg	*g*	**a**	acacacggac	*48*	**43**	>	2	0.05	D	increased chronic post-traumatic nociceptive behavior	↓	Missig et al., 2017
	rs990428608	cgccgcgcag	*g*	**a**	gacacacgga	*48*	**36**	>	5	10^{-3}	B		↓	
	rs374295312	ccccaactgt	*g*	**t**	tgtgtgacag	*19*	**16**	>	4	10^{-3}	B		↓	
	rs375617970	ctgatatctt	*g*	**a**	ttgtgttcct	*12*	**9**	>	5	10^{-6}	A		↓	
	rs368788347	cagagacaca	*t*	**c**	tggggctgac	*25*	**29**	<	2	0.05	D	reduced aggressive social behavior	↓	Nicot et al., 2004
	rs949639399	tgatatcttg	*t*	**c**	tgtgttcctt	*12*	**19**	<	9	10^{-6}	A		↓	
ARTN, 603886	rs572822960	aacccaacaa	*g*	**a**	gcaggtgaac	*44*	**33**	>	5	10^{-6}	A	increased antidepressant-like behavior	↑	Di Cesare Mannelli et al., 2011
	rs967372171	ggagactaga	*c*	**t**	cagagtcaga	*44*	**35**	>	3	10^{-3}	B		↑	
	rs527812444	ccgacgggtg	*g*	**t**	agcagccagg	*64*	**30**	>	11	10^{-6}	A		↑	
	rs958975178	caggtgagcc	*c*	**t**	cgaaaggtgg	*74*	**62**	>	4	10^{-3}	B		↑	
	rs541549283	ctgagctgcc	*c*	**t**	tcaagagacc	*50*	**42**	>	3	10^{-2}	C		↑	
	rs992967159	aaggaggtag	*g*	**a**	aggcagggcc	*50*	**21**	>	13	10^{-6}	A		↑	
	rs752075644	aaggcggctt	*g*	**t**	actggtgagg	*19*	**12**	>	8	10^{-6}	A		↑	
	rs763575206	acctggtact	*g*	**a, t**	aggaaaggcg	*19*	**5**	>	17	10^{-6}	A		↑	
	rs760435671	ctacctggta	*c*	**t**	tgaggaaagg	*19*	**14**	>	5	10^{-3}	B		↑	
	rs751500103	aggcactgcc	*a*	**g**	ggtgtacagt	*11*	**14**	<	3	0.05	D	deficient innervation of tissues with cell death because of reduced neurotrophic support	↓	Honma et al., 2002
	rs774936509	cctacctggt	*a*	**g**	ctgaggaaag	*19*	**47**	<	14	10^{-6}	A		↓	
	rs771912026	ctcctacctg	*g*	**a**	tactgaggaa	*19*	**32**	<	8	10^{-6}	A		↓	

Notes: hereinafter, Alleles: wt, ancestral; mut, minor; "–", deletion; K_D, dissociation constant of TBP–DNA complex; α = 1 – p, significance (where p value is given in Figure 1); **Δ, changes**: excess (>) and deficit (<); **Ss, Social status**: dominance (↑) and subordination (↓); ρ, heuristic rank of candidate SNP markers varying in alphabetical order from the "best" (A) to the "worst" (E). [*)]This SNP also includes other neutral alleles. [#)]Distance from this SNP to a given alternative transcription start site (TSS) whose transcription activity is altered by this SNP. ClinVar, the database of clinical annotations of SNPs (Landrum et al., 2014); Reference, the *italicized references* found by our manual keyword search in the PubMed database (Figure S1, Supplementary file 1) the contents of which are *italicized* in the third rightmost column. **Genes**: ADCYAP1s, adenylate cyclase–activating polypeptide 1 and its receptor; *ARTN*, artemin; *BDNF*, brain-derived neurotrophic factor; *CDNF*, cerebral dopamine neurotrophic factor; *CNTF*, ciliary neurotrophic factor; *FGF*s, fibroblast growth factors and their receptors; *FLT*s, Fms-related tyrosine kinases and their ligand; *GDNF*, glial-cell–derived neurotrophic factor; *GFR*s, GDNF family receptors; *GMF*s, glia maturation factors; *IGF*s, insulin-like growth factors and their receptors; *IL*s, interleukins as well as their receptors and signal transducers; LIFs, leukemia-inhibitory factor (IL6-family cytokine) and its receptor; *NGF*s, nerve growth factor and its receptor; *NRG*s, neuregulins; *NRP*s, neuropilins; *NRTN*, neurturin; *NTF*s, neurotrophins; *NTRK*s, neurotrophic receptor tyrosine kinases; *OSM*s, oncostatin M and its receptor; *PDGF*s, platelet-derived growth factor subunits and receptors; *PGF*, placental growth factor; *PSPN*, persephin; *RET*, Ret receptor tyrosine kinase; *TGFB*s, transforming growth factors β, their receptors and associated protein 1; *VEGF*s, vascular endothelial growth factors. **Deletion/insertion**: FGF1: 12 bp = gaaaaagagaga; FGF11: 12 bp = ccgcccccctgcc; FGF22: 18 bp = gtctctgtctccctccgg; FLT1: 7 bp = aattcca; FLT4: 22 bp = ctgtgccttagctaagcggcca; GDNF: 6 bp = cggcgg; IGF1R: 18 bp = ggcggcgctgagggagga, 23 bp = ggcggcggcggcgctgagggagg; IL6ST: 15 bp = gggattggggagcgc, 22 bp = gttggccggccgggggcgcagc; IL11: 8 bp = ccccggca; NRG2: 16 bp = gggggcgcagcgcggc; NRTN: 9 bp = ctgcagcc; NTRK3: 5 bp = acccc; OSMR: 12 bp = caatgtttccat.

Supplementary Material

Table S3. Continued

Gene, OMIM	dbSNP (Sherry et al., 2001)	5′ flank	wt	**mut**	3′ flank	K_D, nM, prediction: wt	**mut**	Δ	Z	α	ρ	**Known physiological or** candidate SNP[$] markers	Ss	**ClinVar** or Reference
BDNF, 113505	rs900181880	acgtgacaaa	*c*	**t**	cgtaaggaag	*17*	**14**	>	3	10^{-2}	C	increased fear-induced aggressive behavior	↑	Ilchibaeva et al., 2018
	rs549649514	cttaattaga	*g*	**t**	atttttaagc	*17*	**7**	>	3	10^{-2}	C		↑	
	rs774974933	agtatcactt	*a*	**c**	attagagatt	*17*	**8**	>	2	0.05	D		↑	
	rs554116130	atatcaagta	*t*	**a**	cacttaatta	*17*	**6**	>	5	10^{-6}	A		↑	
	rs1051771585	aatatcaagt	*a*	**t**	tcacttaatt	*17*	**8**	>	2	0.05	D		↑	
	rs368334830	tctgtttaag	*c*	**t**	cggcgtgttt	*10*	**7**	>	6	10^{-6}	A		↑	
	rs778895755	gaacttgggt	*g*	**a*)**	ctgggatggg	*48*	**37**	>	4	10^{-3}	B		↑	
	rs768585661	cccgggaaac	*g*	**a**	cacgccctct	*48*	**39**	>	4	10^{-3}	B		↑	
	rs539103547	gggaagcttg	*c*	**t*)**	gccttcagcc	*142*	**66**	>	13	10^{-6}	A		↑	
	rs781368365	aaacagcgag	*g*	**c, t**	ttagtcgtcg	*27*	**20**	>	5	10^{-3}	B		↑	
	rs979555776	gtatcactta	*atta*	**–**	gagattttta	*17*	**10**	<	2	0.05	D	increased aggressive behavior	↑	Ito et al., 2011
	rs1023770367	tttttaaaag	*t*	**c**	aggataaact	*4*	**6**	<	6	10^{-6}	A		↑	
	rs967857379	cactcagctt	*t*	**g**	ttaaaagtag	*4*	**7**	<	9	10^{-6}	A		↑	
	rs922197320	gcggaggtat	*c*	**g**	atatgacagc	*7*	**10**	<	7	10^{-6}	A		↑	
CDNF, 611233	rs545895677	ccttcgctcc	*g*	**t**	gagtgtcctc	*129*	**66**	>	14	10^{-6}	A	improved post-injury axonal neuron regeneration	↑	Lindahl et al., 2017
	rs753172356	atcgccaggc	*g*	**a*)**	cgaggccggg	*129*	**103**	>	4	10^{-3}	B		↑	
	rs958561072	tcccttcctt	*g*	**a, t**	agagccaatg	*26*	**16**	>	8	10^{-6}	A		↑	
	rs977035066	ggctcctaca	*c*	**t**	tccgcccccct	*26*	**19**	>	5	10^{-3}	B		↑	
	rs929441844	cggagtgtcc	*t*	**c**	cgcggcaacg	*129*	**157**	<	3	10^{-3}	B	reduced post-injury axonal neuron regeneration	↓	
CNTF, 118945	rs536642046	atcccattag	*t*	**c**	agagaatgcc	*4*	**29**	<	23	10^{-6}	A	increased depressive-like behavior	↓	Peruga et al., 2012
FGF1, 131220	rs10674553	gagaaagaga	–	**12 bp*)**	aaaagagaga	*29*	**23**	>	5	10^{-3}	B	accelerated skin wound healing	↑	Sun et al., 1997
	rs201326082	gggagaaaga	*g*	**a**	aaagagaaaa	*29*	**25**	>	3	10^{-2}	C		↑	
	rs1033702098	cagcttaaag	*a*	**t**	gagcaccaac	*10*	**9**	>	2	0.05	D		↑	
	rs540994897	aataagatca	*c*	**t**	atgagagggg	*14*	**10**	>	7	10^{-6}	A		↑	
	rs753389103	aacctgatgt	*g*	**a**	catgccacgg	*38*	**10**	>	20	10^{-6}	A		↑	
	rs200890547	aagagaaaaa	*g*	**a**	agagagagaa	*29*	**31**	<	2	0.05	D	delayed skin wound healing	↓	Peplow, Baxter, 2012
	rs202038412	gagaaagaga	*a*	**g**	aaagagagag	*29*	**35**	<	4	10^{-3}	B		↓	
	rs34975836	agagaaagag	–	**ga**	aaaaagagag	*29*	**36**	<	6	10^{-6}	A		↓	
	rs946076749	ccgtcttctg	*t*	**c**	ctctacctcc	*29*	**37**	<	9	10^{-6}	A		↓	
	rs752370034	atgtaaaaat	*a*	**t**	gtacttctag	*5*	**8**	<	6	10^{-6}	A		↓	
	rs1006869201	gacatgtaaa	*a*	**g**	atagtacttc	*5*	**6**	<	3	10^{-2}	C		↓	
FGF3, 164950	rs143969364	cgcgaagccg	*g*	**t**	agtcagcatc	*73*	**43**	>	7	10^{-6}	A	improved post-traumatic neuron regeneration	↑	Goldshmit et al., 2012
FGF4, 164980	rs994443937	caggagaacg	*c*	**a, t**	gaacggcccc	*78*	**47**	>	10	10^{-6}	A	improved post-traumatic neural-tissue survival	↑	Srivastava et al., 2006
	rs955591135	gggcgcccag	*g*	**t**	agaacgcgaa	*78*	**19**	>	22	10^{-6}	A		↑	
FGF5, 165190	rs984068192	ggagcagtga	*g*	**a**	atcactggcg	*47*	**38**	>	4	10^{-3}	B	increased tissue remodeling as complication of chronic post-traumatic inflammation	↓	Sautter et al., 2012
	rs980623288	gtacacaaag	*c*	**a, t**	cgcgggtgag	*6*	**8**	<	4	10^{-3}	B	slowed post-traumatic axonal repair	↓	Barrette et al., 2010
FGF6, 134921	rs763957773	cttaggtaat	*g*	**a**	tggcccccct	*11*	**9**	>	3	10^{-2}	C	increased tissue remodeling as complication of chronic post-traumatic inflammation	↓	Sautter et al., 2012
	rs550517769	tcatctgcct	*t*	**c**	ccaaaaaataa	*15*	**17**	<	2	0.05	D	impaired post-injury skeletal muscle regeneration	↓	Liu et al., 2017
	rs753727963	aagccttagg	*t*	**c**	aatgtggccc	*11*	**33**	<	15	10^{-6}	A		↓	
FGF8, 600483	rs1036153984	ccggcagcgg	*c*	**t**	gcacagcgat	*224*	**132**	>	9	10^{-6}	A	reduced stress-induced anxiety-like behavior	↓	Brooks et al., 2014
	rs993107382	cgcacccgca	*c*	**a, t**	cctctccgct	*187*	**152**	>	4	10^{-3}	B		↓	
	rs866323990	ccgccgggct	*c*	**t**	tcggtgcccc	*157*	**103**	>	8	10^{-6}	A		↓	
	rs756187925	gacggcgtga	*c*	**t**	ccgccgggct	*157*	**121**	>	5	10^{-3}	B		↓	
	rs940142999	ggcagcggcg	*c*	**–**	acagcgattc	*224*	**256**	<	3	0.05	D	increased stress-induced anxiety-like behavior	↑	
	rs879555604	agccgcgtcg	*t*	**a**	gatcgccgcc	*73*	**100**	<	7	10^{-6}	A		↑	
FGF9, 600921	rs753940318	gggagatttg	*t*	**c**	cgccgccacc	*25*	**50**	<	11	10^{-6}	A	worse post-traumatic skin wound healing	↓	Komi-Kuramochi et al., 2005

Supplementary Material

Table S3. Continued

Gene, OMIM	dbSNP (Sherry et al., 2001)	5′ flank	wt	**mut**	3′ flank	K_D, nM, prediction						**Known physiological or** candidate SNP$ markers	Ss	**ClinVar** or Reference
						wt	**mut**	Δ	Z	α	ρ			
FGF10, 602115	rs907033203	aagataccgt	*c*	**t**	agcgccgtgg	*27*	**18**	>	5	10^{-6}	A	improved skin wound healing	↑	Komi-Kuramochi et al., 2005
	rs938496979	gggaagatac	*c*	**a, t**	gtcagcgccg	*27*	**19**	>	5	10^{-6}	A		↑	
FGF11, 601514	rs1002212696	cccagcgtct	*12 bp*	**−**	ccgcccccgg	*35*	**26**	>	5	10^{-6}	A	improved post-injury skin repair	↑	Lin et al., 2014
	rs1019561393	tctcccggcc	*a*	**g**	gaggtttctc	*39*	**47**	<	3	10^{-3}	B	worse vasculogenesis during post-injury wound healing	↓	Cheng et al., 2018
FGF12, 601513	rs941301178	gcgggaggcg	*g*	**t**	tgaaaagggg	*37*	**21**	>	11	10^{-6}	A	fast increase in aversive behavior from generation to generation because of prenatal stress	↓	McCreary et al., 2016
	rs763919094	aatctgctgt	*g*	**a, t**	catccagaga	*31*	**30**	>	6	10^{-3}	B		↓	
	rs538740895	gctgtgcatc	*c*	**t**	agagagcaaa	*41*	**19**	>	13	10^{-6}	A		↓	
	rs966516371	tgcaaatctg	*ctg*	**−**	tgcatccaga	*41*	**31**	>	6	10^{-6}	A		↓	
	rs976229214	tgcaaatctg	*c*	**t**	tgtgcatcca	*41*	**31**	>	6	10^{-6}	A		↓	
	rs1035276723	attttgtatt	*g*	**t**	attttgtatt	*6*	**7**	<	2	0.05	D	slow increase in aversive behavior from generation to generation because of prenatal stress	↑	
	rs959200999	tattttgtat	*t*	**c**	gttttgtttt	*6*	**7**	<	2	0.05	D		↑	
	rs191273550	ggtattttgt	*a*	**g**	ttgttttgtt	*6*	**7**	<	2	0.05	D		↑	
FGF13, 300070	rs187191248	aattcctgtg	*c*	**t**	tgcgactgtg	*51*	**45**	>	3	10^{-2}	C	improved repair, regeneration, and regrowth of injured axons	↑	Li et al., 2018
	rs926021072	tctctgtttg	*c*	**t**	ctgctgtcgt	*47*	**21**	>	17	10^{-6}	A		↑	
	rs942902495	cacaagctct	*c*	**t**	tgtttgcctg	*47*	**25**	>	12	10^{-6}	A		↑	
	rs1039856639	ctgatggttt	*c*	**t*)**	gaaaacagga	*47*	**25**	>	13	10^{-6}	A		↑	
	rs932547015	acaccttgcc	*g*	**t**	tttatataga	*1.0*	**1.2**	<	3	10^{-2}	C	slowed post-traumatic functional recovery of central nervous system	↓	
	rs931591294	tcattttgtt	*a*	**c**	tcattttgtt	*5*	**7**	<	15	10^{-6}	A		↓	
	rs753372648	gagttcattt	*t*	**−**	gttatgtggt	*5*	**7**	<	3	10^{-3}	B		↓	
FGF14, 601515	rs568733534	gacccgttcc	*c*	**t**	ggggccacca	*207*	**124**	>	10	10^{-6}	A	improved motor activity, coordination and balance	↑	Bosch et al., 2015
	rs764921436	cggacccgtt	*c*	**t*)**	ccggggccac	*207*	**155**	>	5	10^{-6}	A		↑	
	rs752244362	ccccggaccc	*g*	**a*)**	ttcccggggc	*207*	**135**	>	8	10^{-6}	A		↑	
	rs757959492	tccccggacc	*c*	**a*)**	gttcccgggg	*207*	**88**	>	8	10^{-6}	A		↑	
	rs777084679	cctccccgga	*c*	**t**	ccgttcccgg	*207*	**147**	>	6	10^{-6}	A		↑	
	rs750943809	cctccctccc	*c*	**t*)**	ggacccgttc	*207*	**142**	>	7	10^{-6}	A		↑	
	rs768849604	cgtcctcccg	*c*	**t**	gccctccctc	*207*	**183**	>	2	0.05	D		↑	
	rs779224822	cgccgtcctc	*c*	**t**	cgcgccctcc	*207*	**173**	>	3	10^{-2}	C		↑	
	rs748248716	cccccggctc	*g*	**a**	ccgtcctccc	*207*	**147**	>	6	10^{-6}	A		↑	
	rs773410343	ggtgcccccg	*g*	**t**	ctcgccgtcc	*207*	**147**	>	3	10^{-6}	A		↑	
	rs986126429	tggtaataat	*c*	**g**	cggtggtgat	*5*	**4**	>	3	10^{-2}	C		↑	
	rs748300605	ccctttggta	*a*	**g**	taatccggtg	*5*	**4**	>	2	0.05	D		↑	
FGF17, 603725	rs547931760	ctgggaattt	*c*	**t**	cactccagag	*43*	**33**	>	5	10^{-6}	A	improved social interaction in novelty exploration	↑	Scearce-Levie et al., 2008
	rs897550388	gctgcttctg	*g*	**t**	gaatttccac	*43*	**35**	>	4	10^{-3}	B		↑	
	rs993234050	tctgggaatt	*t*	**c**	ccactccaga	*43*	**83**	<	11	10^{-6}	A	worse complex social behavior	↓	
FGF18, 603726	rs1005246908	gtagaaaacg	*c*	**t**	cccggccgcc	*9*	**11**	<	3	10^{-2}	C	reduced cerebral blood flow leads to motor and exploratory behavior deficits	↓	Ellsworth et al., 2003
FGF19, 603891	rs564710219	gcaatttacc	*c*	**t**	ggataaaagg	*6*	**5**	>	3	10^{-2}	C	increased heart and coronary artery protection against stress	↑	Hao et al., 2013
FGF20, 605558	rs182128570	aagatcgctc	*c*	**t**	aagacagtga	*33*	**19**	>	7	10^{-6}	A	increased anti-traumatic protection of the nigrostriatal tract with retention of both gross locomotion and fine motor movement	↑	Sleeman et al., 2012
	rs182128570	aaggagagaa	*g*	**t*)**	atcgctccaa	*33*	**25**	>	4	10^{-3}	B		↑	
	rs539071122	tcccacctac	*c*	**g**	caaggagaga	*33*	**25**	>	4	10^{-3}	B		↑	
	rs933127630	cggctcccac	*c*	**t**	tacccaagga	*33*	**14**	>	13	10^{-6}	A		↑	
	rs779946155	gcacctgcac	*g*	**a, t*)**	gcatcctgcg	*123*	**95**	>	4	10^{-3}	B		↑	
	rs763226027	gctggcgcac	*c*	**t**	tgcacggcat	*123*	**47**	>	17	10^{-6}	A		↑	
	rs748250152	gggctgcgca	*g*	**a**	ctggcgcacc	*123*	**91**	>	5	10^{-6}	A		↑	
	rs991685834	gccgggggct	*g*	**a**	cgcagctggc	*123*	**51**	>	13	10^{-6}	A		↑	
	rs746934075:**t**	cgcacctgca	*c*	**t*)**	ggcatcctgc	*123*	**75**	>	9	10^{-6}	A		↑	
	rs746934075:**g**	cgcacctgca	*c*	**g*)**	ggcatcctgc	*123*	**145**	>	3	10^{-2}	C	low anti-traumatic protection of the dopaminergic neurons	↓	

Supplementary Material

Table S3. Continued

Gene, OMIM	dbSNP (Sherry et al., 2001)	5′ flank	wt	**mut**	3′ flank	K_D, nM, prediction: wt	**mut**	Δ	Z	α	ρ	**Known physiological or** candidate SNP[$] markers	Ss	**ClinVar** or Reference
FGF21, 609436	rs575169157	atgaggttga	*g*	**a**	gttggcccac	*37*	**31**	>	3	10^{-2}	C	increased depression-like behavior	↓	Chang et al., 2018
	rs191407470	gtctgagcat	*c*	**t**	tgagcaggga	*37*	**25**	>	8	10^{-6}	A		↓	
FGF22, 605831	rs897054983	cccgccgcgc	*g*	**a**	aaggcagagc	*201*	**94**	>	15	10^{-6}	A	reduced depression-like behavior	↑	Xu Y.H. et al., 2017b
	rs555299241	attggccccg	*c*	**t**	cgcgcgaagg	*201*	**153**	>	5	10^{-6}	A		↑	
	rs1003108764	cattggcccc	*g*	**a**	ccgcgcgaag	*201*	**157**	>	5	10^{-6}	A		↑	
	rs1051130233	ccgtctttct	*18 bp*	**−**	gtctctgtct	*50*	**21**	>	16	10^{-6}	A		↑	
FGFR1, 136350	rs17182058	tgagagagcg	*a*	**g**	gccctcgcgc	*79*	**71**	>	3	10^{-2}	C	improved post-traumatic wound healing without scar formation	↑	Cobden et al., 2016
	rs925214533	gggtgggagt	*g*	**a**	agagagcgag	*79*	**20**	>	27	10^{-6}	A		↑	
	rs550847402	cttcccgccg	*c*	**t**	ccaacttttc	*122*	**98**	>	4	10^{-3}	B		↑	
	rs990541953	ccgctcttcc	*c*	**a**	gccgcccaac	*122*	**89**	>	6	10^{-6}	A		↑	
	rs17175687	cccctccctg	*c*	**t**	gctcgtcccg	*122*	**68**	>	12	10^{-6}	A		↑	
	rs1043293328	gcgccccctc	*c*	**t**	ctgcgctcgt	*122*	**79**	>	9	10^{-6}	A		↑	
	rs17182099	cgcgtctcct	*c*	**t**	cgccccctcc	*122*	**46**	>	17	10^{-6}	A		↑	
	rs4647906	cgcgctttgc	*c*	**t**	cgccgcagcc	*147*	**88**	>	10	10^{-6}	A		↑	
	rs575773912	ccgctgctat	*g*	**a**	tctagggcct	*8*	**3**	>	11	10^{-6}	A		↑	
	rs960719039: 8[#)]	ccgcccaact	*t*	**c**	ttcctccaac	*122*	**107**	>	3	10^{-2}	C		↑	
	rs960719039: 13[#)]	ccgcccaact	*t*	**c**	ttcctccaac	*34*	**82**	<	18	10^{-6}	A	impaired post-traumatic wound healing	↓	
	rs959077050	agccgcatta	*tta*	**−**	acttccctct	*5*	**15**	<	16	10^{-6}	A		↓	
	rs980807486	tgctatgtct	*a*	**g**	gggcctgaca	*8*	**11**	<	4	10^{-3}	B		↓	
FGFR2, 176943	rs886046768	agagcgcggt	*g*	**a**	gagagccgag	*116*	**31**	>	22	10^{-6}	A	**craniosynostosis**	↑	**Landrum et al., 2014**
	rs913507040	ggcgtacctg	*g*	**a**	cccggcgcgg	*42*	**30**	>	4	10^{-3}	B	*predisposition to craniosynostosis* as well as accelerated ulcer healing	↑	*Mansukhani et al., 2000;* as well as Baatar et al., 2002
	rs946170523	ctgcatgcgg	*c*	**t**	gtacctggcc	*42*	**22**	>	8	10^{-6}	A		↑	
	rs767413285	ttaacgtcca	*c*	**a**	atggagatat	*15*	**12**	>	3	10^{-2}	C		↑	
	rs755803072	atgggattaa	*c*	**t[*)]**	gtccacatgg	*15*	**10**	>	10	10^{-6}	A		↑	
	rs986245867	aaacggctcg	*g*	**a**	gtttcagtgg	*30*	**24**	>	4	10^{-3}	B		↑	
	rs757197396	cgtccacatg	*g*	**c**	agatatggaa	*15*	**12**	>	4	10^{-3}	B		↑	
	rs1014850980	ttgcctgcgc	*g*	**t, c**	ctctgagcct	*136*	**116**	>	3	10^{-2}	C		↑	
	rs778187292	cctgtatggt	*g*	**a**	gtaacagtca	*13*	**7**	>	9	10^{-6}	A		↑	
	rs563645875:t	tcactccaaa	*c*	**t**	gtactgactg	*12*	**8**	>	6	10^{-6}	A		↑	
	rs1027484343:37,44[#)]	ctggaggaga	*g*	**t[*)]**	cgcggtggag	*116*	**101**	>	3	10^{-2}	C		↑	
	rs1027484343: 8[#)]	ctggaggaga	*g*	**t[*)]**	cgcggtggag	*128*	**152**	<	3	10^{-3}	B	delayed skin wound healing as well as *predisposition to bent bone dysplasia syndrome*	↓	Meyer et al., 2012; as well as *Merrill et al., 2012*
	rs563645875:g	tcactccaaa	*c*	**g**	gtactgactg	*12*	**23**	<	8	10^{-6}	A		↓	
	rs906542867	ggcggcggcg	*(gcg)*$_{2-3}$	**−**	cgggcggcgg	*30*	**213**	<	31	10^{-6}	A		↓	
	rs905126001	cgggtttcag	*t*	**g**	gggggcgtga	*30*	**59**	<	11	10^{-6}	A		↓	
	rs1002048385	acggctcggg	*t*	**g**	ttcagtgggg	*30*	**51**	<	9	10^{-6}	A		↓	
	rs1009263660	gcctgcgcgc	*t*	**c**	ctgagccttc	*136*	**170**	<	4	10^{-3}	B		↓	
	rs751951199	aatcgcctgt	*a*	**g**	tggtggtaac	*13*	**30**	<	13	10^{-6}	A		↓	
	rs757648006	tcttaatcgc	*c*	**g**	tgtatggtgg	*13*	**17**	<	3	10^{-2}	C		↓	
	rs934459569	attaaaccgg	*g*	**t**	agggctttgt	*8*	**9**	<	2	0.05	D		↓	
	rs387906677	atcgcctgta	*t*	**g**	ggtggtaaca	*13*	**27**	<	9	10^{-6}	A	**bent bone dysplasia syndrome**	↓	**Landrum et al., 2014**
FGFR3, 134934	rs1022054412	cctcccgccg	*g*	**t**	tgcccgcgcc	*393*	**224**	>	11	10^{-6}	A	hypersensitivity to neuropathic mechanical pain	↓	Chen et al., 2015
	rs988202796	cggcgcccgc	*c*	**a, t**	tcccgccggt	*393*	**246**	>	10	10^{-6}	A		↓	
	rs973412538	agcccaggct	*c*	**t**	agtgcgcggt	*103*	**24**	>	25	10^{-6}	A		↓	
	rs531000581	gggcagccca	*g*	**c**	gctcagtgcg	*103*	**89**	>	2	0.05	D		↓	
	rs1028686653	gtcccccact	*g*	**a**	gctgcggcgc	*103*	**49**	>	13	10^{-6}	A		↓	
	rs545082474	gaccgtcccc	*c*	**a, t**	actggctgcg	*103*	**80**	>	5	10^{-3}	B		↓	
	rs373209526	ccgccaacac	*c*	**t**	gtccgcttcc	*79*	**58**	>	6	10^{-6}	A		↓	
	rs577990843	caagaagctg	*c*	**t[*)]**	tggccgtgcc	*79*	**53**	>	8	10^{-6}	A		↓	

Supplementary Material

Table S3. Continued

Gene, OMIM	dbSNP (Sherry et al., 2001)	5′ flank	wt	**mut**	3′ flank	K_D, nM, prediction: wt	**mut**	Δ	Z	α	ρ	**Known physiological or** candidate SNP[$] markers	Ss	**ClinVar** or Reference
FGFR4, 134935	rs773962203	ctgtctgtgt	*g*	**a**	tgtccatgtg	*23*	**10**	>	12	10^{-6}	A	predisposition to muscle dystrophy	↓	Saito et al., 2000
	rs3091253	aggggcgggg	*t*	**c, g**	gggacaggag	*94*	**145**	<	8	10^{-6}	A	reduced post-injury muscle regeneration via replacement by fat and calcification	↓	Zhao et al., 2006
	rs942282462	ctccctattt	*t*	**g**	aggaaggcag	*7*	**10**	<	5	10^{-3}	B		↓	
	rs573476556	cctccctatt	*t*	**c**	taggaaggca	*7*	**9**	<	3	10^{-2}	C		↓	
	rs759087410	tgtctgtgtg	*t*	**c**	gtccatgtgc	*23*	**35**	<	7	10^{-6}	A		↓	
FGFRL1, 605830	rs556824232	ccggcacctg	*c*	**t**	cggcctgtcc	*94*	**59**	>	8	10^{-6}	A	enhanced slow fibers in skeletal muscle	↑	Amann et al., 2014
	rs569782184	actgcccgct	*c*	**t**	attcgggggc	*94*	**23**	>	23	10^{-6}	A		↑	
	rs868584324	gagcgcccga	*g*	**a, t**	cccggacccc	*541*	**323**	>	9	10^{-6}	A		↑	
	rs938279276	ccccgcgccc	*c*	**t**	acgtgggccc	*87*	**25**	>	18	10^{-6}	A		↑	
	rs763426754	tctcccagtt	*c*	**g**	cacgtgttag	*29*	**23**	>	4	10^{-3}	B		↑	
	rs1051752375	tatctcccag	*t*	**g**	tccacgtgtt	*29*	**25**	>	3	10^{-2}	C		↑	
	rs950538892	tgcccgctca	*t*	**c**	tcgggggccg	*94*	**149**	<	9	10^{-6}	A	weakened slow fibers in skeletal muscle	↓	
	rs1026135849	ctgcccgctc	*a*	**c, t**	ttcgggggcc	*94*	**111**	<	3	10^{-2}	C		↓	
	rs766479251	agttccacgt	*g*	**c**	ttagtgacgg	*29*	**33**	<	2	0.05	D		↓	
FLT1, 165070	rs753840461	tcttcatcga	*g*	**a**	gtccgcggga	*26*	**19**	>	6	10^{-6}	A	higher risks of chronic non-healing status of wounds	↓	Zhou et al., 2015
	rs1043619638	gctggggaaa	*g*	**c**	gttataaatc	*3*	**2**	>	3	10^{-2}	C		↓	
	rs570882629	tgggagactc	*g*	**a**	aatggtaatg	*15*	**12**	>	5	10^{-6}	A		↓	
	rs1012402727	ggaaaggtta	*t*	**c**	aaatcgcccc	*3*	**8**	<	15	10^{-6}	A	predisposition to hypertrophy and fibrosis of heart in overload	↓	Mei et al., 2015
	rs971978099	tttctttaca	*7 bp*	**−**	gagttccctc	*7*	**9**	<	4	10^{-3}	B		↓	
	rs917847085	ggcatttctt	*t*	**c**	acaaattcca	*7*	**18**	<	15	10^{-6}	A		↓	
FLT4, 136352	rs916733203	gcctgaatcc	*c*	**t**	gggcccgcct	*81*	**64**	>	4	10^{-3}	B	higher risks of exacerbated inflammatory neovascularization in chronic injury	↓	Leedom et al., 2010
	rs1044187743	ggcgcgggga	*g*	**a**	cggcctgaat	*81*	**69**	>	3	10^{-2}	C		↓	
	rs963866478	ccccaggcca	*g*	**t**	ccggcgcccg	*475*	**263**	>	11	10^{-6}	A		↓	
	rs995645078	ccgccccagg	*c*	**t**	cagccggcgc	*475*	**124**	>	24	10^{-6}	A		↓	
	rs1026679240	cccgccccag	*g*	**a**	ccagccggcg	*475*	**131**	>	23	10^{-6}	A		↓	
	rs950998663	ccccgcccca	*g*	**a**	gccagccggc	*475*	**294**	>	9	10^{-6}	A		↓	
	rs992373585	ccgcctccgg	*c*	**t**	ccccgccccg	*475*	**309**	>	8	10^{-6}	A		↓	
	rs370763296	gggtcggggc	*c*	**a**	ggtgtgaggc	*72*	**56**	>	4	10^{-3}	B		↓	
	rs142395534	cggggacctg	−	**22 bp**	ccagggtcgg	*72*	**18**	>	20	10^{-6}	A		↓	
	rs569295679	ggggaacggg	*g*	**t**[*)]	acctgccagg	*72*	**49**	>	6	10^{-6}	A		↓	
	rs776740749	gggtggcggg	*g*	**t**[*)]	aacggggacc	*72*	**41**	>	9	10^{-6}	A		↓	
	rs373327722	tgtgaggccc	*g*	**t**	tgtcccctcc	*72*	**81**	<	2	0.05	D	narrowed lumen of arteries in vessel remodeling under stress	↓	Baeyens et al., 2015
	rs377358639	gtcggggccg	*g*	**c**	tgtgaggccc	*72*	**90**	<	4	10^{-3}	B		↓	
FLT3LG, 600007	rs762760044	ctgtctcccc	*c*	**a**[*)]	caaaaatttc	*39*	**23**	>	10	10^{-6}	A	enhanced anti-bacterial clearance in post-injury skin wound healing	↑	Bohannon et al., 2010
	rs763513799	gaggggcggt	*g*	**a**[*)]	gggggatgac	*51*	**46**	>	2	0.05	D		↑	
	rs369176469	ccttccaaca	*c*	**t**	agccccatct	*45*	**17**	>	15	10^{-6}	A		↑	
	rs781738148	caggactgct	*c*	**t**	cttccaacac	*45*	**41**	>	2	0.05	D		↑	
	rs747638047	gggatgacg	*c*	**t**	ggtggtgacg	*51*	**77**	<	8	10^{-6}	A	slow innate immune response in post-injury wound healing	↓	Zhang et al., 2013
	rs748705220	gctccttcca	*a*	**g**	cacagcccca	*45*	**75**	<	9	10^{-6}	A		↓	
GDNF, 600837	rs752035330	cagccctcgc	*c*	**t**	ctgttggcgg	*101*	**68**	>	7	10^{-6}	A	improved post-injury spinal cord recovery	↑	Tian et al., 2013
	rs554953764	gcggcggcgg	−	**6 bp**[*)]	gagtcttggc	*101*	**145**	<	7	10^{-6}	A	reduced locomotor activity	↓	Littrell et al., 2013
GFRA1, 601496	rs920008633:**t**	gctctccgct	*c*	**t**	tcatctcaaa	*26*	**18**	>	8	10^{-6}	A	reduced neuropathic pain sensitivity	↑	Dong et al., 2005
	rs920008633:**g**	gctctccgct	*c*	**g**	tcatctcaaa	*26*	**31**	<	3	10^{-2}	C	reduced locomotor activity	↓	Zaman et al., 2008
	rs570918962	tccgctctca	*t*	**c**	ctcaaagcgc	*26*	**42**	<	9	10^{-6}	A		↓	
GFRA2, 601956	rs771505612	aggggggcgt	*c*	**t**	gagaggcagc	*85*	**53**	>	9	10^{-6}	A	improved post-injury survival of all motoneurons except oculomotor and abducens nerves	↑	Oppenheim et al., 2000
GFRA3, 605710	rs531569391	ctagtcgaac	*t*	**c**	tgtcagacag	*46*	**51**	<	2	0.05	D	accelerated neurodegeneration of somatosensory system	↓	Wang, Albers, 2009

Supplementary Material

Table S3. Continued

Gene, OMIM	dbSNP (Sherry et al., 2001)	5′ flank	wt	**mut**	3′ flank	K_D, nM, prediction						**Known physiological or** candidate SNP[$] markers	Ss	**ClinVar** or Reference
						wt	**mut**	Δ	Z	α	ρ			
GFRA4, no ID	rs760523788	atccttctct	*g*	**a**	ttgaaggtcc	*19*	**14**	>	6	10^{-6}	A	increased neuronal survival and neurite outgrowth	↑	Yang et al., 2004
	rs756460522	gaagggggcg	*c*	**a, t[*)]**	ctatcagact	*19*	**14**	>	6	10^{-6}	A		↑	
	rs751545976	gcctatcaga	*c*	**t[*)]a**	tagggctctg	*19*	**22**	<	2	0.05	D	reduced neuronal survival and neurite outgrowth	↓	
	rs767608244	ggcgcctatc	*a*	**g**	gactagggct	*19*	**35**	<	11	10^{-6}	A		↓	
	rs750696535	ggggcgccta	*t*	**a, g**	cagactaggg	*19*	**23**	<	3	10^{-2}	C		↓	
GMFB, 601713	rs181263382	catgactttt	*a*	**t**	agtatgcacc	*4*	**6**	<	6	10^{-6}	A	reduced neurodegenerative effects on motor coordination in brain injury	↑	Khan et al., 2015
	rs962051030	ccagcgggcc	*a*	**g**	tgggactagg	*31*	**35**	<	2	0.05	D		↑	
GMFG, 604104	rs745952352	acgcctagaa	*g*	**t**	acagcggaac	*17*	**10**	>	8	10^{-6}	A	better wound healing via improved angiogenesis	↑	Zuo et al., 2013
	rs757388319	ggtccccacg	*c*	**a**	ctagaagaca	*17*	**10**	>	7	10^{-6}	A		↑	
	rs775463657	cggtccccac	*g*	**a**	cctagaagac	*17*	**12**	>	5	10^{-6}	A		↑	
	rs181638961	gcccgccccc	*c*	**a, t[*)]**	tacagccccg	*30*	**19**	>	6	10^{-6}	A		↑	
	rs769527080	cacgcctaga	*a*	**–**	gacagcggaa	*17*	**32**	<	10	10^{-6}	A	worse wound healing because of impaired angiogenesis	↓	
	rs191075835	gcgccgtgcg	*t*	**c[*)]**	caccgcccct	*140*	**248**	<	11	10^{-6}	A		↓	
IGF1, 147440	rs936770538	ttactcaata	*a*	**c**	ctttgccaga	*10*	**12**	<	4	10^{-3}	B	increased depression-like behavior	↓	Mitschelen et al., 2011
	rs369361539	ttgtttcaat	*c*	**t**	gacaaaaggc	*16*	**19**	<	3	10^{-2}	C		↓	
IGF1R, 147370	rs111811434	ccggcccgcc	*g*	**a**	ctttgtgtgt	*12*	**10**	>	3	10^{-2}	C	facilitated pain sensitivity when tissue is damaged	↑	Miura et al., 2011
	rs565450721	gagggaggag	*g*	**a**	cggcggcgag	*150*	**110**	>	6	10^{-6}	A		↑	
	rs891986395	ccgcggcggc	*18 bp*	**–**	ggcggcggcg	*150*	**65**	>	17	10^{-6}	A		↑	
	rs1053681925	agctcgccgc	*23 bp*	**–**	aggcggcggc	*150*	**60**	>	18	10^{-6}	A		↑	
	rs758662662	tgtgtccaga	*c*	**t**	aggaatacag	*17*	**14**	>	3	10^{-2}	C		↑	
	rs3138594	cggagccagg	*agg*	**–**	aggaggagga	*150*	**213**	<	7	10^{-6}	A	increased susceptibility to skin injury and worse post-injury skin wound healing	↓	Bentov et al., 2014
	rs533518937	gagcggagcc	*agg*	**–**	aggaggagga	*150*	**213**	<	7	10^{-6}	A		↓	
	rs139163109	gagcggagcc	*–*	**gga, ggg[*)]**	aggaggagga	*150*	**207**	<	7	10^{-6}	A		↓	
	rs747468693	acaggaatac	*a*	**g**	ggaagtatgg	*17*	**20**	<	2	0.05	D		↓	
	rs778092387	gtgtccagac	*a*	**c**	ggaatacagg	*17*	**20**	<	2	0.05	D		↓	
IGF2, 147470	rs899552468	gcgactataa	*g*	**a**	agccgggcgt	*2.4*	**1.6**	>	6	10^{-6}	A	increased antidepressant-like behavior	↑	Grieco et al., 2017
	rs893349307	gggccgctcc	*c*	**g**	ctttccgcgc	*106*	**123**	<	3	10^{-2}	D	increased depression-like behavior	↓	Lim et al., 2018
	rs574896585	cgtgtccagg	*a*	**c**	aagcgaccgg	*47*	**63**	<	5	10^{-6}	A		↓	
IL6, 147620	rs2069827	ctgttttatc	*g*	**t[*)]**	atcttgaaga	*7*	**5**	>	3	10^{-2}	C	higher risks of stress-induced depression-like behavior	↓	Trystula et al., 2017
	rs527770772	ccaacaaaga	*t*	**c**	ttatcaaatg	*11*	**9**	>	3	10^{-2}	C		↓	
	rs746737342	caccgggaac	*g*	**a[*)]**	aaagagaagc	*36*	**25**	>	8	10^{-6}	A		↓	
	rs201575115	agagtctcaa	*c*	**a**	ccccaataaa	*3*	**5**	<	6	10^{-6}	A	reduced depression-like behavior	↑	Kong et al., 2015
	rs898211454	actggagatg	*t*	**c**	ctgaggctca	*36*	**53**	<	8	10^{-6}	A		↑	
	rs779901687	aggactggag	*a*	**g**	tgtctgaggc	*36*	**49**	<	5	10^{-6}	A		↑	
IL6R, 147880	rs770930455	caccccagca	*g*	**c**	atgggctggc	*64*	**39**	>	9	10^{-6}	A	increased pain sensitivity	↓	Strickland et al., 2014
	rs147437437	aagccggctg	*c*	**t**	aggctcccac	*64*	**54**	>	2	0.05	D		↓	
	rs1019106367	actgggtgct	*c*	**t**	aggaagccgg	*64*	**30**	>	13	10^{-6}	A		↓	
	rs759580674	agcagatggg	*c*	**t**	tggcatggga	*64*	**73**	<	3	10^{-2}	C	reduced pain sensitivity	↑	
	rs774440162	cagcagatgg	*g*	**c**	ctggcatggg	*64*	**74**	<	3	10^{-2}	C		↑	
	rs1011958394	ttctacatag	*t*	**c**	gtccatgtgc	*4*	**5**	<	5	10^{-6}	A		↑	
	rs766792218	agctctttct	*a*	**g**	catagtgtcc	*4*	**12**	<	17	10^{-6}	A		↑	
IL6ST, 600694	rs1001902576	gcgcagctgg	*g*	**a**	cgggattggg	*165*	**121**	>	6	10^{-6}	A	increased sensitivity to fatigue	↓	Gray et al., 2008
	rs1033697726	ggggcgcagc	*22 bp*	**–**	tgggcgggat	*165*	**108**	>	7	10^{-6}	A		↓	
	rs753501869	acatgtgtct	*gt*	**–**	tgcagaaata	*4*	**3**	>	3	10^{-2}	C		↓	
	rs57236815	tacatgtgtc	*tg*	**–**	ttgcagaaat	*4*	**3**	>	3	10^{-2}	C		↓	
	rs369036454	aagagaaaaa	*a*	**g**	tattaaaagt	*4*	**3**	>	6	10^{-6}	A		↓	
	rs535927064	ggcgcgctac	*c*	**t**	tctgcggaga	*49*	**29**	>	9	10^{-6}	A		↓	
	rs569406527	ccgcgggact	*g*	**a[*)]**	gggtggcgcg	*49*	**39**	>	3	10^{-3}	B		↓	
	rs775209835:**a**	taaattatgt	*g*	**a**	tatataagag	*1.0*	**0.7**	>	5	10^{-6}	A		↓	
	rs775209835:**t**	taaattatgt	*g*	**t**	tatataagag	*1.0*	**1.4**	<	4	10^{-3}	B	reduced sensitivity to pain	↑	Andratsch et al., 2009
	rs1002376913	gcagctgggc	*15 bp*	**–**	cccgccccgc	*165*	**207**	<	4	10^{-3}	B		↑	
	rs369959492	aatattaaaa	*g*	**a**	taatattaca	*3.7*	**4.3**	<	2	0.05	D		↑	
	rs997152259	aaaaatatta	*a*	**g**	aagtaatatt	*3.7*	**4.3**	<	2	0.05	D		↑	
	rs910298633	ctctctttac	*a*	**g**	gtgaaaccca	*5*	**6**	<	2	0.05	D		↑	
	rs373078515	gcgctacctc	*t*	**c, g[*)]**	gcggagaagg	*49*	**59**	<	3	10^{-2}	C		↑	
	rs1038178387	cgcgctacct	*c*	**t**	tgcggagaag	*49*	**57**	<	3	10^{-2}	C		↑	

Supplementary Material

Table S3. Continued

Gene, OMIM	dbSNP (Sherry et al., 2001)	5′ flank	wt	**mut**	3′ flank	K_D, nM, prediction						**Known physiological or** candidate SNP$^{\$}$ markers	Ss	**ClinVar** or Reference
						wt	**mut**	Δ	Z	α	ρ			
IL11, 147681	rs553507807	ctcggggctc	*6 bp*	**–**	ctcggggctc	*160*	**72**	>	14	10^{-6}	A	reduced depression-like behavior	↑	Pusic et al., 2014
	rs978083181	gggcggggca	*g*	**a**	ccagagccag	*145*	**89**	>	10	10^{-6}	A		↑	
	rs934227915	gcgtctgctc	*c*	**t**	gacgggcggg	*145*	**81**	>	11	10^{-6}	A		↑	
	rs561820040	cccgggccgc	*g*	**a**	tctgctccga	*145*	**85**	>	10	10^{-6}	A		↑	
	rs985622223	ccccgggccg	*c*	**t**$^{*)}$	gtctgctccg	*145*	**62**	>	15	10^{-6}	A		↑	
IL11RA, 600939	rs754749137	ttgactctac	*c*	**t**	tctccccaca	*16*	**13**	>	4	10^{-3}	B	increased sensory neuron survival in neurodegenerative trauma	↑	Their et al., 1999
	rs780140398	atttttgact	*c*	**t**	tacctctccc	*16*	**14**	>	2	0.05	D		↑	
	rs1032155727	aaagcactgg	*g*	**t**	tatacagtgg	*4.4*	**3.6**	>	2	0.05	D		↑	
	rs574333714	gtgtgtgtcc	*g*	**a**	tgtgtgtgtg	*18*	**14**	>	6	10^{-6}	A		↑	
	rs144043150	gtgtgtgtcc	–	**at**$^{*)}$	gtgtgtgtgt	*18*	**14**	>	6	10^{-6}	A		↑	
	rs1010025315	agggggcctg	*g*	**c**	ctagggggcgg	*126*	**96**	>	4	10^{-3}	B		↑	
	rs13300653	ggcaggagca	*g*	**a**	ggagggggcc	*126*	**110**	>	2	0.05	D		↑	
IL27, 608273	rs767614290	cagtataaga	*c*	**a**$^{*)}$	ccccccctacc	*3*	**2**	>	3	10^{-2}	C	increased depression-like behavior	↓	Chitu et al., 2015
	rs375439697	gcccccagta	*t*	**g**	aagaccccccc	*3*	**13**	<	21	10^{-6}	A	predisposition to sepsis reducing post-injury survival	↓	Yan et al., 2016
LIFR, 151443	rs934938783	gcggcgtgca	–	**a**	ggcgccgccc	*165*	**90**	>	11	10^{-6}	A	increased depression-like behavior	↓	Maes et al., 2002
	rs549886719	gagcggcgtg	*c*	**t**	aggcgccgcc	*165*	**29**	>	28	10^{-6}	A		↓	
	rs896028952	cccagccgag	*c*	**t**	ggcgtgcagg	*165*	**121**	>	6	10^{-6}	A		↓	
	rs1013074495	gcccccagcc	*g*	**t**$^{*)}$	agcggcgtgc	*165*	**129**	>	4	10^{-3}	B		↓	
	rs1044528837	gctgcgcccc	*c*	**t**$^{*)}$	agccgagcgg	*165*	**135**	>	3	10^{-3}	B		↓	
	rs535151782	tctgaacttt	*g*	**a**	caaaattaat	*12*	**7**	>	8	10^{-6}	A		↓	
NGFR, 162010	rs1003002976	tggcttcacc	*c*	**t**	agcctctccc	*89*	**28**	>	20	10^{-6}	A	increased emotional anxiolytic-like behavior	↓	Kasuya et al., 2013
	rs532346435	tgctgactaa	*c*	**t**	gccgctggtt	*24*	**13**	>	10	10^{-6}	A		↓	
	rs535209739	gaggcaagtg	*c*	**t**	agtccgccgc	*92*	**14**	>	25	10^{-6}	A		↓	
NRG1, 142445	rs913880282	gtgccctctt	*g*	**a**	gcccaccgcc	*162*	**36**	>	27	10^{-6}	A	reduced neuropathic-pain sensitivity	↑	Wang G. et al., 2013
	rs879701096	accgtgccct	*c*	**t**	ttggcccacc	*162*	**90**	>	12	10^{-6}	A		↑	
	rs772632425	caacgggacc	*g*	**a**$^{*)}$	tgccctcttg	*162*	**145**	>	2	0.05	D		↑	
	rs745448521	ccaacgggac	*c*	**t**$^{*)}$	gtgccctctt	*162*	**139**	>	3	10^{-2}	C		↑	
	rs778723461	ccgccaacgg	*g*	**a**$^{*)}$	accgtgccct	*162*	**118**	>	6	10^{-6}	A		↑	
	rs552381232	ccgaggagcc	*g*	**a**	ctgctcgccg	*162*	**122**	>	5	10^{-6}	A		↑	
	rs36213231	aggctcctcc	*c*	**t**$^{*)}$	ggtggcgtgt	*89*	**77**	>	2	0.05	D		↑	
	rs564814775	aggcgcctgc	*c*	**t**	tccaacctgc	*99*	**73**	>	6	10^{-6}	A		↑	
	rs548361366	gctccctgca	*g*	**a**	gcaacgggag	*147*	**68**	>	14	10^{-6}	A		↑	
	rs923812011	acccactcgc	*g*	**a**	ggtcccgctc	*152*	**108**	>	6	10^{-6}	A		↑	
	rs754364784	agacccatct	*c*	**t**	ttgatgggct	*39*	**26**	>	8	10^{-6}	A		↑	
	rs199775395	agtgcagacc	*c*	**t**	atctcttgat	*39*	**21**	>	11	10^{-6}	A		↑	
	rs74893081	cagctgagtg	*c*	**t**	agacccatct	*39*	**18**	>	12	10^{-6}	A		↑	
	rs895250429	cctctgcgtg	*g*	**a**	taatggaccg	*21*	**17**	>	4	10^{-3}	B		↑	
	rs377497653	ggggcctctg	*c*	**a, t**	gtggtaatgg	*21*	**18**	>	3	10^{-2}	C		↑	
	rs771113096	cattcccttg	*g*	**t**	catctagaga	*17*	**13**	>	4	10^{-3}	B		↑	
	rs760753911	gaaaactaat	*g*	**a**	actccaccta	*17*	**8**	>	11	10^{-6}	A		↑	
	rs923023850	agctacatta	*g*	**–**	actccaccta	*8*	**6**	>	3	10^{-2}	C		↑	
	rs777137810	ggagagtata	*t*	**c**	gtgcaaagtg	*2*	**6**	<	14	10^{-6}	A	increased aggressiveness during social contacts	↑	O'Tuathaigh et al., 2008
	rs762362331	acccatctct	*t*	**c**	gatgggcttc	*39*	**58**	<	84	10^{-6}	A		↑	
	rs560821644	aaactaatga	*c*	**t**	tccacctatc	*17*	**20**	<	4	10^{-3}	B		↑	
	rs773264121	tggaaaacta	*a*	**g**	tgactccacc	*17*	**22**	<	5	10^{-6}	A		↑	
	rs531395089	agagaagcta	*c*	**a**	attaggtggc	*8*	**12**	<	8	10^{-6}	A		↑	

Supplementary Material

Table S3. Continued

Gene, OMIM	dbSNP (Sherry et al., 2001)	5′ flank	wt	**mut**	3′ flank	K_D, nM, prediction: wt	**mut**	Δ	Z	α	ρ	**Known physiological or** candidate SNP[$] markers	Ss	**ClinVar** or Reference
	rs769308716	cccccaaatg	*g*	**–**	cctggccaga	*58*	**47**	>	4	10^{-3}	B		↑	
	rs367804259	cccaccgcct	*g*	**t**	ggaccccgtg	*158*	**124**	>	5	10^{-3}	B		↑	
	rs747268987	ccgtcccacc	*g*	**a**[*)]	cctgggaccc	*158*	**106**	>	7	10^{-6}	A		↑	
	rs770404206	gagagctgag	*g*	**a**	ccgtcccacc	*158*	**132**	>	3	10^{-3}	B	increased anxiety-like behavior	↑	Diaz-Moran et al., 2013
NRG2, 603818	rs565936260	agcgcggcag	*c*	**a**	ggagagctga	*158*	**133**	>	3	10^{-3}	B		↑	
	rs749613530	tgcaggcggc	*16 bp*	**–**	agcggagagc	*158*	**131**	>	4	10^{-3}	B		↑	
	rs369753660:**t**	cggagccccc	*c*	**t**	aaatggcctg	*58*	**17**	>	22	10^{-6}	A		↑	
	rs369753660:**g**	cggagccccc	*c*	**g**	aaatggcctg	*58*	**68**	<	3	10^{-2}	C		↓	
	rs763517342	gagcccccca	*a*	**g**[*)]	atggcctggc	*58*	**91**	<	8	10^{-6}	A	reduced anxiety-like behavior	↓	Yan et al., 2018
	rs1036285416	ggcgctaacg	*t*	**c**	tacgctgttt	*19*	**33**	<	10	10^{-6}	A		↓	
	rs775903748	ccagattttt	*g*	**t**	atctgctctt	*17*	**7**	>	18	10^{-6}	A	increased impulsive behavior	↑	
NRG3, 605533	rs917457901	tagaagataa	*a*	**g**	ttgggagaca	*9*	**13**	<	7	10^{-6}	A		↓	Loos et al., 2014
	rs1884280	tgatctgctc	*t*	**a**	tttgatgcag	*17*	**20**	<	4	10^{-3}	B	reduced impulsive responding	↓	
	rs981163111	ggggcgaagg	*t*	**c**	gaagaccggc	*64*	**161**	<	18	10^{-6}	A		↓	
NRG4, 610894	rs886519666	cctcgcctcc	*c*	**t**	gccggccgcg	*374*	**216**	>	10	10^{-6}	A	improved oxidative balance reducing aggressiveness	↓	Slattery et al., 2015; Garratt, Brooks, 2015
	rs950135265	ctgcgccctc	*g*	**a**	cctcccgccg	*374*	**111**	>	21	10^{-6}	A		↓	
	rs764376860	tcccgcgtct	*g*	**a**	cctgcggacc	*73*	**35**	>	13	10^{-6}	A		↑	
	rs750728269	tcctcgttct	*c*	**g**	ccgcgtctgc	*73*	**61**	>	4	10^{-3}	B		↑	
	rs368605791	ttccccccac	*g*	**t**	tcctcgttct	*73*	**62**	>	4	10^{-3}	B		↑	
	rs770826613	gaccacgctt	*g*	**a**	tacttgtctt	*12*	**5**	>	13	10^{-6}	A		↑	
	rs778973864	cactgaccac	*g*	**a, c**	cttgtacttg	*12*	**8**	>	4	10^{-3}	B		↑	
	rs1014331980	tcatcaggat	*c*	**t**	taccccgaga	*37*	**29**	>	5	10^{-3}	B		↑	
	rs764907882	tctccacgcg	–	**gtag**	attcatcagg	*37*	**31**	>	3	10^{-2}	C		↑	
	rs773172539	ctctccacgc	*g*	**a**	attcatcagg	*37*	**33**	>	2	0.05	D	improver post-injury muscle regeneration	↑	Wagatsuma, 2007
	rs200246620	ctctctccac	*g*	**a**[*)]	cgattcatca	*37*	**27**	>	6	10^{-6}	A		↑	
	rs767534920	ttccagctct	*c*	**a**	tccacgcgat	*37*	**28**	>	4	10^{-3}	B		↑	
	rs775164675	cggacttttc	*c*	**t**	agctctctcc	*37*	**9**	>	27	10^{-6}	A		↑	
	rs765366437	ggattgctgt	*g*	**a**	gatgacatta	*27*	**18**	>	8	10^{-6}	A		↑	
	rs767349233	ccgcctggta	*a*	**t**	ccagtcgctc	*33*	**29**	>	2	0.05	D		↑	
NRP1, 602069	rs540725422	catccgcctg	*g*	**a**	taaccagtcg	*33*	**16**	>	12	10^{-6}	A		↑	
	rs938895415	agattatcct	*g*	**a**	gaatttgaaa	*23*	**16**	>	6	10^{-6}	A		↑	
	rs766540692	tcagagatta	*t*	**a**	cctggaattt	*23*	**17**	>	5	10^{-6}	A		↑	
	rs28527211	tttttttttt	*t*	**c**	ttttttgaga	*24*	**28**	<	3	10^{-2}	C		↓	
	rs566779182	gagacacaaa	*a*	**g**	agagaagcgt	*11*	**14**	<	6	10^{-6}	A		↓	
	rs933271099	tgagacacaa	*a*	**g**	aagagaagcg	*11*	**17**	<	10	10^{-6}	A		↓	
	rs375566888	aggcgacttg	*a*	**g**	gacacaaaaa	*11*	**12**	<	3	10^{-2}	C		↓	
	rs749023218	acgcttgtac	*t*	**c**	tgtcttgcag	*12*	**15**	<	4	10^{-3}	B		↓	
	rs748317563	agataataat	*c*	**t**	cttggatctt	*7*	**9**	<	4	10^{-3}	B	predisposition to sepsis reducing post-injury survival	↓	Dai et al., 2017
	rs773278916	tgtgagataa	*t*	**c**	aatccttgga	*7*	**11**	<	8	10^{-6}	A		↓	
	rs374304776	ggtgtgagat	*a*	**g, c**	ataatccttg	*7*	**13**	<	9	10^{-6}	A		↓	
	rs774720126	aggtgtgaga	*t*	**c**	aataatcctt	*7*	**13**	<	9	10^{-6}	A		↓	
	rs200660300	aacatccgcc	*g*	**t**	ggtaaccagt	*33*	**58**	<	9	10^{-6}	A		↓	
	rs199740711	cagagattat	*c*	**t**	ctggaatttg	*23*	**28**	<	3	10^{-2}	C		↓	
	rs774782566	gtcagagatt	*a*	**t**	tcctggaatt	*23*	**28**	<	4	10^{-3}	B		↓	
NRP2, 602070	rs974971969	agaaaaacac	*a*	**c**	aagatttaaa	*6*	**5**	>	2	0.05	D	higher risks of post-traumatic neointimal hyperplasia of vascular smooth muscle	↓	Pellet-Many et al., 2015
	rs774739180	caaagattta	*a*	**g**	acaagaaacc	*6*	**13**	<	13	10^{-6}	A	preference for social novelty because of weakened object recognition memory	↑	Shiflett et al., 2015
	rs778187703	acaaagattt	*a*	**t**	aacaagaaac	*6*	**10**	<	8	10^{-6}	A		↑	
	rs113815476	acacaaagat	*t*	**g**	taaacaagaa	*6*	**8**	<	4	10^{-3}	B		↑	

Supplementary Material

Table S3. Continued

Gene, *OMIM*	dbSNP (Sherry et al., 2001)	5′ flank	wt	**mut**	3′ flank	K$_D$, nM, prediction: wt	**mut**	Δ	Z	α	ρ	**Known physiological or** candidate SNP[$] markers	Ss	**ClinVar** or Reference
NRTN, 602018	rs531950590	ggcctccttg	*c*	**t**	tgttcctggg	*38*	**27**	>	7	10^{-6}	A	behavioral hypersensitivity to environmental stimuli	↓	Wang T. et al., 2013
	rs576981082	tggctcctca	–	**9 bp**	gctgcagccg	*38*	**85**	<	16	10^{-6}	A	impaired innervation of organs such as heart and eye	↓	Downs et al., 2016
	rs533504972	cctttcactc	*c*	**t**	tccccctggc	*38*	**49**	<	5	10^{-6}	A		↓	
NTF3, 162660	rs71532852	ccctcacagg	*g*	**t**	ctactcagcc	*39*	**21**	>	8	10^{-6}	A	increased attention-deficit behavior	↓	Bilgic et al., 2017
	rs573875777	cgtgccctca	*c*	**t**[*)]	agggctactc	*39*	**22**	>	8	10^{-6}	A		↓	
	rs1009831233	taccctctct	*c*	**t**	gtgccctcac	*39*	**31**	>	3	10^{-2}	C		↓	
	rs982691988	tggctggtta	*t*	**c**	aaccgcgcag	*5*	**14**	<	12	10^{-6}	A	increased depression-like behavior	↓	Oglodek et al., 2016
NTF4, 162662	rs1031777123	tcctctgccg	*c*	**a, t**	ccggcgccgc	*143*	**105**	>	6	10^{-6}	A	increased manic-depression-like behavior	↓	Tseng et al., 2016
	rs531235522	ccacctgcct	*c*	**t**	tataacctgt	*5*	**3**	>	8	10^{-6}	A		↓	
NTRK1, 191315	rs1009139776	ccgcccagcg	*c*	**t**	acatgtcggg	*11*	**7**	>	6	10^{-6}	A	reduced neuropathic allodynia	↑	Wu C. et al., 2016
	rs917244438	cgtcagagag	*t*	**c**	aggaagcggg	*27*	**58**	<	13	10^{-6}	A	increased self-mutilating behavior	↓	Indo, 2002
NTRK2, *600456*	rs937639671	tcttacgcgt	*g*	**a**	tctgtttgtc	*15*	**11**	>	5	10^{-3}	B	reduced depression-like behavior	↑	Zhu et al., 2015
	rs577271234	cagtcttacg	*c*	**t**	gtgtctgttt	*15*	**11**	>	6	10^{-6}	A		↑	
	rs199568649	tcagtctta	*c*	**t**	gcgtgtctgt	*15*	**8**	>	10	10^{-6}	A		↑	
	rs563746574	cctacgctca	*g*	**c**	tcttacgcgt	*15*	**14**	>	2	0.05	D		↑	
NTRK3, 191316	rs960861259	ctccgataac	*c*	**t**	cgtgcgtttc	*29*	**13**	>	12	10^{-6}	A	increased panic behavior due to improved fear memory	↓	Santos et al., 2013
	rs534372666	tgttgcaact	*g*	**a**	gacccctctt	*58*	**36**	>	9	10^{-6}	A		↓	
	rs1021213050	gcagctgttg	*c*	**a**	aactggaccc	*58*	**28**	>	13	10^{-6}	A		↓	
	rs552794563	ccggaagatg	*a*	**t**	ggtcggcagc	*58*	**35**	>	10	10^{-6}	A		↓	
	rs781578330	ccccccctctc	*c*	**t**	tctcctcctc	*119*	**49**	>	18	10^{-6}	A		↓	
	rs746068935	accccccctct	*c*	**t**	ctctcctcct	*119*	**49**	>	18	10^{-6}	A		↓	
	rs771055278	cccccacccc	*5 bp*	**–**	cctctcctct	*119*	**44**	>	20	10^{-6}	A		↓	
	rs566027273	tccccactcc	*c*	**t**[*)]	cccaccccac	*119*	**92**	>	5	10^{-3}	B		↓	
	rs139639386	cccctgctct	*c*	**t**	cccactcccc	*119*	**96**	>	4	10^{-3}	B		↓	
	rs944569547	agcacattgt	*c*	**a, g**	aagttctatg	*43*	**39**	>	2	0.05	D		↓	
	rs751327117	tgcagcatga	*g*	**a, c**	cacattgtca	*43*	**36**	>	4	10^{-3}	B		↓	
	rs757138874	ccgagctgct	*c*	**t**	accaacctgc	*43*	**21**	>	13	10^{-6}	A		↓	
	rs755542029	gcttcaccct	*g*	**t**	acgtgcattg	*30*	**13**	>	15	10^{-6}	A		↓	
	rs550578284	cgagtgagga	*c*	**t**	aatggcttca	*30*	**23**	>	5	10^{-3}	B		↓	
	rs781493247	tgaatgtgac	*g*	**a**	agtgaggaca	*30*	**27**	>	2	0.05	D		↓	
	rs187007070	gtgaatgtga	*c*	**g**[*)]	gagtgaggac	*30*	**27**	>	3	0.05	D		↓	
	rs774240394	tgacgctggt	*g*	**a**	aatgtgacga	*30*	**10**	>	19	10^{-6}	A		↓	
	rs747378024	agcgcgcctc	*g*	**a, t**[*)]	cttccagagc	*99*	**79**	>	4	10^{-3}	B		↓	
	rs960893436	ggagagcgcg	*c*	**t**[*)]	ctcgcttcca	*99*	**85**	>	3	10^{-2}	C		↓	
	rs758791090	tcctcttttt	*t*	**g, c**	agaagcagcg	*8*	**28**	<	22	10^{-6}	A	increased anhedonic depressive behavior	↓	Farhang et al., 2014
	rs147833081	ccccacccca	*c*	**t**	ccccctctcc	*119*	**133**	<	2	0.05	D		↓	
	rs534653573	caggctgtga	*c*	**t**[*)]	gtcacaaagg	*20*	**23**	<	2	0.05	D		↓	
OSM, 165095	rs1049925681	gaaggagcca	*c*	**t**	ttatggagag	*38*	**26**	>	8	10^{-6}	A	lesser lesion size and faster recovery after brain/spinal cord injury	↑	Slaets et al., 2014
OSMR, 601743	rs931880437	cgcaaagtcc	*g*	**a**	ggttgtgagg	*45*	**34**	>	6	10^{-6}	A	worse skin wound healing because of greater size of lesions with severe pruritus	↓	Dillon et al., 2004
	rs930392237	ctcttctcac	*c*	**t**	agtgcccgcc	*45*	**16**	>	18	10^{-6}	A		↓	
	rs574451237	cctcttctca	*c*	**t**	cagtgcccgc	*45*	**30**	>	8	10^{-6}	A		↓	
	rs375663420	acttcctctt	*c*	**t**	tcaccagtgc	*45*	**31**	>	8	10^{-6}	A		↓	
	rs780269025	tcaagcagta	*12 bp*	**–**	caaggtgaac	*14*	**9**	>	5	10^{-6}	A		↓	
	rs188634744	tctcttttttt	*g*	**a**[*)]	atcaagcagt	*14*	**9**	>	8	10^{-6}	A		↓	
	rs755116094	gcagtacaat	*g*	**c**	tttccatcaa	*14*	**17**	<	4	10^{-3}	B	worse wound healing because of deficit of platelets and their progenitors	↓	Tanaka, M. et al., 2003

Supplementary Material

Table S3. Continued

Gene, OMIM	dbSNP (Sherry et al., 2001)	5′ flank	wt	**mut**	3′ flank	K_D, nM, prediction: wt	**mut**	Δ	Z	α	ρ	**Known physiological or** candidate SNP[$] markers	Ss	**ClinVar** or Reference
PDGFA, 173430	rs533482707	ggcggcggcc	–	**(ggc)₃**	ccagactccc	*89*	**109**	<	4	10^{-3}	B	lower risks of atrial fibrillation inducing stroke and heart failure under stress	↑	Liao et al., 2010
PDGFB, 190040	rs888083607	cctcgctgca	*a*	**g**	agagaaaacc	*30*	**26**	>	3	10^{-2}	C	increased explorative behavior	↑	Niklasson et al., 2010
	rs764391387	gtgtgtgtgt	*g*	**a**	cgcatctgag	*18*	**15**	>	3	10^{-2}	C		↑	
	rs762931476	gtgtgtgtgt	–	**ac**	gcgcatctga	*18*	**13**	>	4	10^{-3}	B		↑	
	rs66513025	tgtgtgtgtg	*tgt*	**cgc**	gcgcatctga	*18*	**13**	>	4	10^{-3}	B		↑	
	rs566842805	ggagcgcgcc	*g*	**t**	cctgcaaaag	*41*	**36**	>	2	0.05	D		↑	
	rs1044055259	tgtcacgtga	*c*	**t**	gcgtctggcc	*45*	**39**	>	3	10^{-2}	C		↑	
	rs938411629	tcccactgtc	*a*	**c, t**	cgtgacgcgt	*45*	**53**	<	3	10^{-2}	C	slowed recovery of motor activity in spinal cord injury	↓	Xiyang et al., 2009
	rs549255120	cgagtcccac	*t*	**c**[*)]	gtcacgtgac	*45*	**53**	<	3	10^{-2}	C		↓	
PDGFC, 608452	rs993346859	cttgtttctt	*c*	**a, t**	cccctccccc	*42*	**48**	<	3	10^{-3}	B	improved motor neuron survival	↑	Lewandowski et al., 2016
PDGFD, 609673	rs771642015	ttgtccttct	*g*	**a**[*)]	cagtaaaacc	*9*	**6**	>	6	10^{-6}	A	improved skin wound healing	↑	Uutela et al., 2004
	rs745421003:**t**	gtccttctgc	*a*	**t**	gtaaaaccac	*9*	**7**	>	5	10^{-6}	A		↑	
	rs745421003:**g**	gtccttctgc	*a*	**g**	gtaaaaccac	*9*	**13**	<	6	10^{-6}	A	delayed skin wound healing	↓	Toriseva et al., 2012
PDGFRA, 173490	rs183431225	tggactataa	*g*	**a**	attgaatcaa	*4*	**3**	>	4	10^{-3}	B	**idiopathic hypereosinophilic syndrome, gastrointestinal stromal tumor**; *predisposition to idiopathic hypereosinophilic syndrome and gastrointestinal stromal tumor,* as well as oligodendrocyte-associated nociceptive hypersensitivity to neuropathic pain	↓	**Landrum et al., 2014**; *Score et al., 2006; Hayashi et al., 2015;* as well as Shi et al., 2016
	rs146337155	gaaattctgt	*g*	**a**	tgtaaacatt	*6*	**3**	>	11	10^{-6}	A		↓	
	rs899357849	gatggactat	*g*	**a**	acattgaatc	*4*	**6**	<	6	10^{-6}	A	higher risks of post-injury glial scar formation in brain and spinal cord	↓	Chen et al., 2016
PDGFRB, 173410	rs1004567051	tgcagccttt	*c*	**t**	ccccaaagtg	*72*	**60**	>	4	10^{-3}	B	improved acute post-traumatic vascular remodeling	↑	Malabanan et al., 2012
	rs555370823	ttcccaaata	*t*	**c**	ttgtcctggc	*10*	**8**	>	3	10^{-2}	C		↑	
	rs542518387	ggatgcttgg	*g*	**a**[*)]	gagtgaggcg	*114*	**86**	>	6	10^{-6}	A		↑	
	rs371860652	tgctcctccc	*g*	**a**	aaggatgctt	*114*	**99**	>	3	10^{-2}	C		↑	
	rs554304774	ctgctcctcc	*g*	**a, t**	gaaggatgct	*114*	**92**	>	4	10^{-3}	B		↑	
	rs540087068	gcccctctgg	*c*	**t**	ggctctgctc	*114*	**75**	>	8	10^{-6}	A		↑	
	rs984412818	tcccaaatat	*t*	**c**	tgtcctggca	*10*	**13**	<	3	10^{-2}	C	worsened post-traumatic skin wound healing	↓	Gao et al., 2005
PDGFRL, 604584	rs992666694	accttacgaa	*g*	**t**	gactggcctg	*17*	**12**	>	5	10^{-6}	A	increased cardiac mass during myocardial hypertrophy in hypertensive behavior	↓	Coan et al., 2017
	rs191564603	tacaccttac	*g*	**t**	aaggactggc	*17*	**7**	>	12	10^{-6}	A		↓	
PGF, 601121	rs918441513	tctcagaagg	*g*	**t**	agctgctgtc	*44*	**29**	>	7	10^{-6}	A	improved post-injury cardiac muscle repair	↑	Zhang et al., 2015
	rs1051295108	gctgcagtct	*c*	**t**	agaagggagc	*44*	**14**	>	21	10^{-6}	A		↑	
	rs892660294	acctcgtctg	*c*	**a**	ggtgggcggg	*104*	**51**	>	14	10^{-6}	A		↑	
	rs1025067034	aactcggctc	*g*	**a, c**	ggaacctcgt	*104*	**92**	>	2	0.05	D		↑	
	rs532908973	tggctcggga	*c*	**t**	gtctgagaag	*98*	**76**	>	5	10^{-6}	A		↑	
	rs956976703	gatgagcatg	*g*	**t**	tggttttccc	*98*	**66**	>	9	10^{-6}	A		↑	
	rs1013722378:**t**	tcgggaacct	*c*	**t**	gtctgcggtg	*104*	**44**	>	16	10^{-6}	A		↑	
	rs1013722378:**g**	tcgggaacct	*c*	**g**	gtctgcggtg	*104*	**146**	<	7	10^{-6}	A	slowed post-injury peripheral nervous system repair	↓	Chaballe et al., 2011
	rs929825857	ctgcagtctc	*a*	**c**	gaagggagct	*44*	**62**	<	6	10^{-6}	A		↓	
	rs12885730	ggctgcagtc	*t*	**c**	cagaagggag	*44*	**62**	<	6	10^{-6}	A		↓	
PSPN, 602921	rs774838152	cccaggcagg	*c*	**t**	cataaatttg	*6*	**5**	>	2	0.05	D	reduced excitotoxic death of motor neurons in overload	↑	Ho et al., 2000

Table S3. Continued

Gene, OMIM	dbSNP (Sherry et al., 2001)	5′ flank	wt	**mut**	3′ flank	K_D, nM, prediction: wt	**mut**	Δ	Z	α	ρ	**Known physiological or** candidate SNP$ markers	Ss	**ClinVar** or Reference
RET, 164761	rs528835015	accccacccg	*c*	**t**	ctccggcccc	*258*	**152**	>	9	10^{-6}	A	hypersensitivity to neuropathic pain as well as *predisposition to renal adysplasia, Hirschsprung disease, pheochromocytoma*	↓	Wang, H. et al., 2017 as well as *Sarin et al., 2014; Ishii et al., 2013; Powers et al., 2009*
	rs1053032841	ccccttcccg	*c*	**t**	accccacccg	*258*	**116**	>	11	10^{-6}	A		↓	
	rs10900297	gcgcttacct	*c*	**a**	gcttcagtcc	*30*	**9**	>	17	10^{-6}	A	**renal adysplasia, Hirschsprung disease, pheochromocytoma**	↓	**Landrum et al., 2014**
	rs10900296	gccggcgctt	*a*	**g, c**	cctcgcttca	*30*	**90**	<	20	10^{-6}	A	*predisposition to renal adysplasia, Hirschsprung disease, pheochromocytoma,* as well as reduced epidermal innervation	↓	*Bridgewater et al., 2008; Zhan et al., 1999; Moore, Zaahl, 2012* as well as Golden et al., 2010
	rs901058241	ccacccctgg	*a*	**c**	ccgcccccgc	*258*	**322**	<	4	10^{-3}	B		↓	
	rs551321384	agccggcgct	*t*	**c**	acctcgcttc	*30*	**75**	<	17	10^{-6}	A		↓	
TGFB1, 190180	rs1016245089	cggcttcaaa	*a*	**c, g**	ccccctgccg	*25*	**41**	<	9	10^{-6}	A	increased depression-like behavior	↓	Myint et al., 2005
	rs139080695	gtgtgtgtgt	*a*	**g**	tgtccccctat	*9*	**14**	<	6	10^{-6}	A		↓	
TGFB2, 190220	rs546214861	tctttaaata	*t*	**c**	ataaatttca	*1.5*	**2.0**	<	4	10^{-3}	C	improved neuroregeneration due to reduced post-traumatic collagen scar in wound healing	↑	Kim et al., 2011
TGFB3, 190230	rs148069039	tgagttcatg	*c*	**t**	accttcttgc	*39*	**9**	>	24	10^{-6}	A	accelerated skin wound healing	↑	Sefat et al., 2014
	rs551929281	agtgagttca	*t*	**c**	gcaccttctt	*39*	**54**	<	6	10^{-6}	A	increased depression-like behavior	↓	Xu et al., 2016
TGFBI, 601692	rs1045084770	agccaggggc	*g*	**a**	cacgggttgg	*106*	**86**	>	4	10^{-3}	B	worsened vision because of TGFBI-induced corneal dystrophy	↓	Han et al., 2016
	rs781740702	gccagtacac	*g*	**a**	cttttggccc	*24*	**13**	>	8	10^{-6}	A		↓	
	rs757860567	ggccagtaca	*c*	**t**	gcttttggcc	*24*	**12**	>	9	10^{-6}	A		↓	
	rs184978714	tgaaggtaac	*g*	**a**	gccagtacac	*24*	**19**	>	3	10^{-2}	C		↓	
	rs753586534	atgcttgaag	*g*	**a, t**	taacggccag	*24*	**15**	>	6	10^{-6}	A		↓	
	rs766085944	gatgcttgaa	*g*	**a**	gtaacggcca	*24*	**20**	>	3	0.05	D		↓	
	rs755874694	cacgatgctt	*g*	**a**	aaggtaacgg	*24*	**12**	>	10	10^{-6}	A		↓	
	rs199852470	caacacgatg	*c*	**t**	ttgaaggtaa	*24*	**19**	>	4	10^{-3}	B		↓	
	rs761773998	tcaacacgat	*g*	**a**	cttgaaggta	*24*	**11**	>	10	10^{-6}	A		↓	
	rs779138447	tgggcagatt	*g*	**a**	taactgtgaa	*10*	**3**	>	14	10^{-6}	A		↓	
	rs779132788	aaataccaca	*t*	**g, c**	tggtgatgaa	*28*	**24**	>	3	10^{-2}	C		↓	
	rs759542534	tgaaatacca	*c*	**t**	attggtgatg	*28*	**13**	>	13	10^{-6}	A		↓	
	rs113538354	ccatcaccaa	*c*	**t**	aacatccagc	*29*	**11**	>	16	10^{-6}	A		↓	
	rs534794507	tccaccatca	*c*	**t**	caacaacatc	*29*	**19**	>	8	10^{-6}	A		↓	
	rs758383396	gcagattgta	*a*	**g**	ctgtgaactg	*10*	**16**	<	6	10^{-6}	A	predisposition to reduced skeleton size and degradation of articular cartilage	↓	Lee et al., 2015
	rs369690547	ggcagattgt	*a*	**g**	actgtgaact	*10*	**38**	<	22	10^{-6}	A		↓	
	rs780973591	atcaccaaca	*a*	**g**	catccagcag	*29*	**52**	<	12	10^{-6}	A		↓	
	rs751300973	accatcacca	*a*	**g**	caacatccag	*29*	**52**	<	11	10^{-6}	A		↓	
TGFBR1, *190181*	rs955149948	ctagggaggt	*g*	**a**	gggcgaggcg	*96*	**41**	>	13	10^{-6}	A	improved neuroregeneration in oral tissue wound healing	↑	Agis et al., 2014
	rs11568745	gccgccgcgg	*c*	**t**	ggctagggag	*96*	**81**	>	3	10^{-2}	C		↑	
	rs898170338	ggccgccgcg	*g*	**a**	cggctaggga	*96*	**69**	>	5	10^{-6}	A		↑	
	rs11568742	ctctgttcta	*c*	**t**	gtaacagcct	*5*	**3**	>	5	10^{-6}	A		↑	
	rs905336450	agcagttaca	*a*	**g**	agggccggag	*6*	**11**	<	8	10^{-6}	A	higher risks for aortic aneurysm deforming aortic wall	↓	Yang P. et al., 2016
	rs1028049974	ccgcggcggc	*t*	**c**	agggaggtgg	*96*	**150**	<	8	10^{-6}	A		↓	

Supplementary Material

Table S3. Continued

Gene, *OMIM*	dbSNP (Sherry et al., 2001)	5′ flank	wt	**mut**	3′ flank	K_D, nM, prediction						**Known physiological or** candidate SNP$^{\$}$ markers	Ss	**ClinVar** or Reference
						wt	**mut**	Δ	Z	α	ρ			
TGFBR2, 190182	rs138010137	cgcagcgctg	*a*	**g**	gttgaagttg	*29*	**39**	<	6	10^{-6}	A	**thoracic aortic aneurysm and aortic dissection**; *predisposition to TGFBR2-deficient aortic aneurysm and aortic dissection* as well as accelerated healing, closure, and resurfacing of skin wounds	↑	**Landrum et al., 2014**; *Angelov et al., 2017* as well as Martinez-Ferrer et al., 2010
	rs774493245	attggcagct	*a*	**g**	cgagagagct	*22*	**24**	<	2	0.05	D		↑	
TGFBR3, 600742	rs929900640	gagcggcact	*t*	**a**	tcctcttccc	*55*	**35**	>	7	10^{-6}	A	accelerated wound healing due to increased vascularization	↑	Wehrhan et al., 2004
	rs537116060	gatgggggta	*c*	**t**	tcgagggttt	*55*	**48**	>	2	0.05	D		↑	
	rs371850227	gtttctgggc	*g*	**t**$^{*)}$	gccgagagcc	*79*	**117**	<	7	10^{-6}	A	slowed oral cavity wound healing in diabetes	↓	Yamano et al., 2013
	rs1046910560	agtttctggg	*c*	**t**	ggccgagagc	*79*	**89**	<	2	0.05	D		↓	
VEGFA, 192240	rs757438449	cttgggatcc	*c*	**a**	gcagctgacc	*195*	**140**	>	5	10^{-6}	A	improved skin wound healing	↑	Wang et al., 2018
	rs370995111	cctcccccctt	*g*	**a**	ggatcccgca	*195*	**53**	>	20	10^{-6}	A		↑	
	rs758827990	ggagcgcggc	*g*	**a**	tgagccctcc	*195*	**140**	>	6	10^{-6}	A		↑	
	rs962048538	ccgccggagc	*g*	**a**$^{*)}$	cggcgtgagc	*195*	**152**	>	4	10^{-3}	B		↑	
	rs992466179	gcggcgcgga	*g*	**a**	ccgattacat	*21*	**15**	>	6	10^{-6}	A		↑	
	rs772861763	ccccagctac	*c*	**t**	acctcctccc	*41*	**14**	>	15	10^{-6}	A		↑	
	rs942729487	cccagcccca	*g*	**a**	ctaccacctc	*41*	**19**	>	11	10^{-6}	A		↑	
	rs781745747	tgctctcttt	*c*	**g**	tgtcctcagt	*28*	**17**	>	9	10^{-6}	A		↑	
	rs762362569	gcgccgagga	*g*	**t**	agcgggccgc	*61*	**54**	>	2	0.05	D		↑	
	rs748501346	ccgaggcgcc	*gag*	**−**	gagagcgggc	*61*	**43**	>	7	10^{-6}	A		↑	
	rs745387465	aggaagagta	*g*	**t**	ctcgccgagg	*61*	**23**	>	16	10^{-6}	A		↑	
	rs780766978	cggggaggaa	*g*	**a**	agtagctcgc	*61*	**44**	>	6	10^{-6}	A		↑	
	rs779923978	cagacagaca	*g*	**a**$^{*)}$	acaccgcccc	*41*	**13**	>	13	10^{-6}	A		↑	
	rs761101895	cgccggcccc	*g*	**t**$^{*)}$	gtcgggcctc	*193*	**150**	>	5	10^{-3}	B		↑	
	rs756935833	agagggagcg	*c*	**t**	gagccgcgcc	*193*	**157**	>	4	10^{-3}	B		↑	
	rs777531850	gccggagagg	*g*	**a**	agcgcgagcc	*193*	**127**	>	8	10^{-6}	A		↑	
	rs372731987	agcccgagcc	*g*	**a, t**	gagagggagc	*193*	**108**	>	11	10^{-6}	A		↑	
	rs765392517	ccgccccaca	*g*	**a, c**	cccgagccgg	*193*	**139**	>	6	10^{-6}	A		↑	
	rs759754009	ggccgcccca	*c*	**t**	agcccgagcc	*193*	**70**	>	17	10^{-6}	A		↑	
	rs754042549	gcgggccgcc	*c*	**t**	cacagcccga	*193*	**123**	>	9	10^{-6}	A		↑	
	rs529911267	ttttttttta	*a*	**g**	aagtcggctg	*5*	**7**	<	6	10^{-6}	A	better learning without depression-like behaviors during helplessness	↑	Reif et al., 2004
	rs562785377	cttttttttt	*t*	**c**$^{*)}$	aaaagtcggc	*5*	**16**	<	22	10^{-6}	A		↑	
	rs747915464	accgccccca	*g*	**a**	ccccagctac	*41*	**51**	<	3	10^{-3}	B		↑	
	rs752674861	ggctcatgga	*c*	**t**	gggtgaggcg	*41*	**54**	<	5	10^{-6}	A		↑	
VEGFB, 601398	rs369397290	ctgacatcac	*c*	**g, t**	catcccactc	*49*	**44**	>	2	0.05	D	improved neurogenesis when brain injury causes central neuronal loss	↑	Sun et al., 2006
	rs140652999	gctgacatca	*c*	**a**	ccatcccact	*49*	**35**	>	7	10^{-6}	A		↑	

References

Agis, H., Collins, A., Taut, A.D., Jin, Q., Kruger, L., Gorlach, C., and Giannobile, W.V. (2014) Cell population kinetics of collagen scaffolds in ex vivo oral wound repair. *PLoS One*. **9**: e112680. doi: 10.1371/journal.pone.0112680

Amann, R., Wyder, S., Slavotinek, A.M., and Trueb, B. (2014) The FgfrL1 receptor is required for development of slow muscle fibers. *Dev Biol*. **394**, 228-241. doi: 10.1016/j.ydbio.2014.08.016

Andratsch, M., Mair, N., Constantin, C.E., Scherbakov, N., Benetti, C., Quarta, S. et al. (2009) A key role for gp130 expressed on peripheral sensory nerves in pathological pain. *J Neurosci*. **29**, 13473-13483. doi: 10.1523/jneurosci.1822-09.2009

Supplementary Material

Angelov, S.N., Hu, J.H., Wei, H., Airhart, N., Shi, M., and Dichek, D.A. (2017) TGF-β (Transforming Growth Factor-β) signaling protects the thoracic and abdominal aorta from angiotensin II-induced pathology by distinct mechanisms. *Arterioscler Thromb Vasc Biol*. **37**, 2102-2113. doi: 10.1161/atvbaha.117.309401

Baatar, D., Kawanaka, H., Szabo, I.L., Pai, R., Jones, M.K., Kitano, S., and Tarnawski, A.S. (2002) Esophageal ulceration activates keratinocyte growth factor and its receptor in rats: implications for ulcer healing. *Gastroenterology.* **122**, 458-468. doi: 10.1053/gast.2002.31004

Baeyens, N., Nicoli, S., Coon, B.G., Ross, T.D., Van den Dries, K., Han, J. et al. (2015) Vascular remodeling is governed by a VEGFR3-dependent fluid shear stress set point. *eLife*. **4**: e04645. doi: 10.7554/eLife.04645

Barrette, B., Calvo, E., Vallieres, N., and Lacroix, S. (2010) Transcriptional profiling of the injured sciatic nerve of mice carrying the Wld(S) mutant gene: identification of genes involved in neuroprotection, neuroinflammation, and nerve regeneration. *Brain Behav Immun*. **24**, 1254-1267. doi: 10.1016/j.bbi.2010.07.249

Bentov, I., Damodarasamy, M., Plymate, S., and Reed, M.J. (2014) Decreased proliferative capacity of aged dermal fibroblasts in a three dimensional matrix is associated with reduced IGF1R expression and activation. *Biogerontology*. **15**, 329-337. doi: 10.1007/s10522-014-9501-8

Bilgic, A., Toker, A., Isık, U., and Kılınc, I. (2017) Serum brain-derived neurotrophic factor, glial-derived neurotrophic factor, nerve growth factor, and neurotrophin-3 levels in children with attention-deficit/hyperactivity disorder. *Eur Child Adolesc Psychiatry*. **26**, 355-363. doi: 10.1007/s00787-016-0898-2

Bohannon, J., Cui, W., Sherwood, E., and Toliver-Kinsky, T. (2010) Dendritic cell modification of neutrophil responses to infection after burn injury. *J Immunol*. **185**, 2847-2853. doi: 10.4049/jimmunol.0903619

Bosch, M.K., Carrasquillo, Y., Ransdell, J.L., Kanakamedala, A., Ornitz, D.M., and Nerbonne, J.M. (2015) Intracellular FGF14 (iFGF14) is required for spontaneous and evoked firing in cerebellar purkinje neurons and for motor coordination and balance. *J Neurosci*. **35**, 6752-6769. doi: 10.1523/jneurosci.2663-14.2015

Bridgewater, D., Cox, B., Cain, J., Lau, A., Athaide, V., Gill, P.S. et al. (2008) Canonical WNT/beta-catenin signaling is required for ureteric branching. *Dev Biol*. **317**, 83-94. doi: 10.1016/j.ydbio.2008.02.010

Brooks, L.R., Pals, H.L., Enix, C.L., Woolaver, R.A., Paul, E.D., Lowry, C.A., and Tsai, P.S. (2014) Fibroblast growth factor 8 deficiency compromises the functional response of the serotonergic system to stress. *PLoS One*. **9**: e101420. doi: 10.1371/journal.pone.0101420

Chaballe, L., Close, P., Sempels, M., Delstanche, S., Fanielle, J., Moons, L. et al. (2011) Involvement of placental growth factor in Wallerian degeneration. *Glia*. **59**, 379-396. doi: 10.1002/glia.21108

Chang, H.H., Chen, P.S., Cheng, Y.W., Wang, T.Y., Yang, Y.K., and Lu, R.B. (2018) FGF21 is associated with metabolic effects and treatment response in depressed bipolar II disorder patients treated with valproate. *Int J Neuropsychopharmacol*. **21**, 319-324. doi: 10.1093/ijnp/pyx093.

Chen, F.F., Huo, F.Q., Xiong, H., Wan, Q., Zheng, Y.N., Du, W.J., and Mei, Z.N. (2015) Analgesic effect of total flavonoids from Sanguis draxonis on spared nerve injury rat model of neuropathic pain. *Phytomedicine*. **22**, 1125-1132. doi: 10.1016/j.phymed.2015.08.011

Chen, Y., Cao, S., Xu, P., Han, W., Shan, T., Pan, J. et al. (2016) Changes in the expression of miR-34a and its target genes following spinal cord injury in rats. *Med Sci Monit*. **22**, 3981-3993. doi: 10.12659/msm.900893

Cheng, S., Cui, Y., Fan, L., Mu, X., and Hua, Y. (2018) T2DM inhibition of endothelial miR-342-3p facilitates angiogenic dysfunction via repression of FGF11 signaling. *Biochem Biophys Res Commun*. **503**, 71-78. doi: 10.1016/j.bbrc.2018.05.179

Supplementary Material

Chitu, V., Gokhan, S., Gulinello, M., Branch, C.A., Patil, M., Basu, R. et al. (2015) Phenotypic characterization of a Csf1r haploinsufficient mouse model of adult-onset leukodystrophy with axonal spheroids and pigmented glia (ALSP). *Neurobiol Dis*. **74**, 219-228. doi: 10.1016/j.nbd.2014.12.001

Coan, P.M., Hummel, O., Garcia Diaz, A., Barrier, M., Alfazema, N., Norsworthy, P.J. et al. (2017) Genetic, physiological and comparative genomic studies of hypertension and insulin resistance in the spontaneously hypertensive rat. *Dis Model Mech*. **10**, 297-306. doi: 10.1242/dmm.026716

Cobden, S.B., Ozturk, K., Duman, S., Esen, H., Aktan, T.M., Avunduk, M.C., and Elsurer, C. (2016) Treatment of acute vocal fold injury with platelet-rich plasma. *J Voice*. **30,** 731-735. doi: 10.1016/j.jvoice.2015.07.012

Dai, X., Okon, I., Liu, Z., Wu, Y., Zhu, H., Song, P., and Zou, M.H. (2017) A novel role for myeloid cell-specific neuropilin 1 in mitigating sepsis. *FASEB J*. **31**, 2881-2892. doi: 10.1096/fj.201601238R

Di Cesare Mannelli, L., Vivoli, E., Salvicchi, A., Schiavone, N., Koverech, A., Messano, M. et al. (2011) Antidepressant-like effect of artemin in mice: a mechanism for acetyl-L-carnitine activity on depression. *Psychopharmacology (Berl).* **218**, 347-356. doi: 10.1007/s00213-011-2326-0

Diaz-Moran, S., Palencia, M., Mont-Cardona, C., Canete, T., Blazquez, G., Martinez-Membrives, E. et al. (2013) Gene expression in hippocampus as a function of differential trait anxiety levels in genetically heterogeneous NIH-HS rats. *Behav Brain Res*. **257**, 129-139. doi: 10.1016/j.bbr.2013.09.041

Dillon, S.R., Sprecher, C., Hammond, A., Bilsborough, J., Rosenfeld-Franklin, M., Presnell, S.R. et al. (2004) Interleukin 31, a cytokine produced by activated T cells, induces dermatitis in mice. *Nat Immunol*. **5**, 752-760. doi: 10.1038/ni1084

Dong, Z.Q., Ma, F., Xie, H., Wang, Y.Q., and Wu, G.C. (2005) Changes of expression of glial cell line-derived neurotrophic factor and its receptor in dorsal root ganglions and spinal dorsal horn during electroacupuncture treatment in neuropathic pain rats. *Neurosci Lett*. **376**, 143-148. doi: 10.1016/j.neulet.2004.11.044

Downs, A.M., Jalloh, H.B., Prater, K.J., Fregoso, S.P., Bond, C.E., Hampton, T.G., and Hoover, D.B. (2016) Deletion of neurturin impairs development of cholinergic nerves and heart rate control in postnatal mouse hearts. *Physiol Rep*. **4:** e12779. doi: 10.14814/phy2.12779

Ellsworth, J.L., Garcia, R., Yu, J., and Kindy, M.S. (2003) Fibroblast growth factor-18 reduced infarct volumes and behavioral deficits after transient occlusion of the middle cerebral artery in rats. *Stroke*. **34**, 1507-1512. doi: 10.1161/01.str.0000071760.66720.5f

Farhang, S., Barar, J., Fakhari, A., Mesgariabbasi, M., Khani, S., Omidi, Y., and Farnam, A. (2014) Asymmetrical expression of BDNF and NTRK3 genes in frontoparietal cortex of stress-resilient rats in an animal model of depression. *Synapse*. **68**, 387-393. doi: 10.1002/syn.21746

Gao, Z., Sasaoka, T., Fujimori, T., Oya, T., Ishii, Y., Sabit, H. et al. (2005) Deletion of the PDGFR-beta gene affects key fibroblast functions important for wound healing. *J Biol Chem*. **280**, 9375-9389. doi: 10.1074/jbc.m413081200

Garratt, M., and Brooks, R.C. (2015) A genetic reduction in antioxidant function causes elevated aggression in mice. *J Exp Biol*. **218**, 223-227. doi: 10.1242/jeb.112011

Golden, J.P., Hoshi, M., Nassar, M.A., Enomoto, H., Wood, J.N., Milbrandt, J. et al. (2010) RET signaling is required for survival and normal function of nonpeptidergic nociceptors. *J Neurosci*. **30**, 3983-3994. doi: 10.1523/jneurosci.5930-09.2010

Goldshmit, Y., Sztal, T.E., Jusuf, P.R., Hall, T.E., Nguyen-Chi, M., and Currie, P.D. (2012) Fgf-dependent glial cell bridges facilitate spinal cord regeneration in zebrafish. *J Neurosci*. **32**, 7477-7492. doi: 10.1523/jneurosci.0758-12.2012.

Supplementary Material

Gray, S.R., Robinson, M., and Nimmo, M.A. (2008) Response of plasma IL-6 and its soluble receptors during submaximal exercise to fatigue in sedentary middle-aged men. *Cell Stress Chaperones*. **13**, 247-251. doi: 10.1007/s12192-008-0019-3

Grieco, S.F., Cheng, Y., Eldar-Finkelman, H., Jope, R.S., and Beurel, E. (2017) Up-regulation of insulin-like growth factor 2 by ketamine requires glycogen synthase kinase-3 inhibition. *Prog Neuropsychopharmacol Biol Psychiatry*. **72**, 49-54. doi: 10.1016/j.pnpbp.2016.08.008

Han, K.E., Choi, S.I., Kim, T.I., Maeng, Y.S., Stulting, R.D., Ji, Y.W., and Kim, E.K. (2016) Pathogenesis and treatments of TGFBI corneal dystrophies. *Prog Retin Eye Res*. **50**, 67-88. doi: 10.1016/j.preteyeres.2015.11.002

Hao, Y., Zhou, J., Zhou, M., Ma, X., Lu, Z., Gao, M. et al. (2013) Serum levels of fibroblast growth factor 19 are inversely associated with coronary artery disease in chinese individuals. *PLoS One*. **8**: e72345. doi: 10.1371/journal.pone.0072345

Hayashi, Y., Bardsley, M.R., Toyomasu, Y., Milosavljevic, S., Gajdos, G.B., Choi, K.M. et al. (2015) Platelet-derived growth factor receptor-α regulates proliferation of gastrointestinal stromal tumor cells with mutations in KIT by stabilizing ETV1. *Gastroenterology*. **149**, 420-432.e16. doi: 10.1053/j.gastro.2015.04.006

Ho, T.W., Bristol, L.A., Coccia, C., Li, Y., Milbrandt, J., Johnson, E. et al. (2000) TGFbeta trophic factors differentially modulate motor axon outgrowth and protection from excitotoxicity. *Exp Neurol*. **161**, 664-675. doi: 10.1006/exnr.1999.7290

Honma, Y., Araki, T., Gianino, S., Bruce, A., Heuckeroth, R., Johnson, E., and Milbrandt, J. (2002) Artemin is a vascular-derived neurotropic factor for developing sympathetic neurons. *Neuron*. **35**, 267-282. doi: 10.1016/S0896-6273(02)00774-2

Ilchibaeva, T.V., Tsybko, A.S., Kozhemyakina, R.V., Kondaurova, E.M., Popova, N.K., and Naumenko, V.S. (2018) Genetically defined fear-induced aggression: Focus on BDNF and its receptors. *Behav Brain Res*. **343**, 102-110. doi: 10.1016/j.bbr.2018.01.034

Indo, Y. (2002) Genetics of congenital insensitivity to pain with anhidrosis (CIPA) or hereditary sensory and autonomic neuropathy type IV. Clinical, biological and molecular aspects of mutations in TRKA(NTRK1) gene encoding the receptor tyrosine kinase for nerve growth factor. *Clin Auton Res*. **12**, I20-I32. doi: 10.1007/s102860200016

Ishii, K., Doi, T., Inoue, K., Okawada, M., Lane, G.J., Yamataka, A., and Akazawa, C. (2013) Correlation between multiple RET mutations and severity of Hirschsprung's disease. *Pediatr Surg Int*. **29**, 157-163. doi: 10.1007/s00383-012-3196-1

Ito, W., Chehab, M., Thakur, S., Li, J., and Morozov, A. (2011) BDNF-restricted knockout mice as an animal model for aggression. *Genes Brain Behav*. **10,** 365-374. doi: 10.1111/j.1601-183X.2010.00676.x

Kasuya, H., Hata, E., Satou, T., Yoshikawa, M., Hayashi, S., Masuo, Y., and Koike, K. (2013) Effect on emotional behavior and stress by inhalation of the essential oil from Chamaecyparis obtusa. *Nat Prod Commun*. **8**, 515-518.

Khan, M.M., Zaheer, S., Thangavel, R., Patel, M., Kempuraj, D., and Zaheer, A. (2015) Absence of glia maturation factor protects dopaminergic neurons and improves motor behavior in mouse model of parkinsonism. *Neurochem Res*. **40**, 980-990. doi: 10.1007/s11064-015-1553-x

Kim, J.H., Lee, Y.W., Park, Y.M., Park, K.A., Park, S.H., Lee, W.T., and Lee, J.E. (2011) Agmatine-reduced collagen scar area accompanied with surface righting reflex recovery after complete transection spinal cord injury. *Spine (Phila Pa 1976)*. **36**, 2130-2138. doi: 10.1097/brs.0b013e318205e3f7

Supplementary Material

Komi-Kuramochi, A., Kawano, M., Oda, Y., Asada, M., Suzuki, M., Oki, J., and Imamura, T. (2005) Expression of fibroblast growth factors and their receptors during full-thickness skin wound healing in young and aged mice. *J Endocrinol*. **186**, 273-289. doi: 10.1677/joe.1.06055

Kong, E., Sucic, S., Monje, F.J., Savalli, G., Diao, W., Khan, D. et al. (2015) STAT3 controls IL6-dependent regulation of serotonin transporter function and depression-like behavior. *Sci Rep*. **5**: 9009. doi: 10.1038/srep09009

Landrum, M.J., Lee, J.M., Riley, G.R., Jang, W., Rubinstein, W.S., Church, D.M., et al. (2014). ClinVar: public archive of relationships among sequence variation and human phenotype. *Nucleic Acids Res*. 42, D980-D985. doi:10.1093/nar/gkt1113

Lee, J.M., Lee, E.H., Kim, I.S., and Kim, J.E. (2015) Tgfbi deficiency leads to a reduction in skeletal size and degradation of the bone matrix. *Calcif Tissue Int*. **96**, 56-64. doi: 10.1007/s00223-014-9938-4

Leedom, A.J., Sullivan, A.B., Dong, B., Lau, D., and Gronert, K. (2010) Endogenous LXA4 circuits are determinants of pathological angiogenesis in response to chronic injury. *Am J Pathol*. **176**, 74-84. doi: 10.2353/ajpath.2010.090678

Lehmann, M.L., Mustafa, T., Eiden, A.M., Herkenham, M., and Eiden L.E. (2013) PACAP-deficient mice show attenuated corticosterone secretion and fail to develop depressive behavior during chronic social defeat stress. *Psychoneuroendocrinology*. **38**, 702-715. doi: 10.1016/j.psyneuen.2012.09.006

Lewandowski, S.A., Nilsson, I., Fredriksson, L., Lonnerberg, P., Muhl, L., Zeitelhofer, M. et al. (2016) Presymptomatic activation of the PDGF-CC pathway accelerates onset of ALS neurodegeneration. *Acta Neuropathol*. **131**, 453-464. doi: 10.1007/s00401-015-1520-2

Li, J., Wang, Q., Wang, H., Wu, Y., Yin, J., Chen, J. et al. (2018) Lentivirus mediating FGF13 enhances axon regeneration after spinal cord injury by stabilizing microtubule and improving mitochondrial function. *J Neurotrauma*. **35**, 548-559. doi: 10.1089/neu.2017.5205

Liao, C.H., Akazawa, H., Tamagawa, M., Ito, K., Yasuda, N., Kudo, Y. et al. (2010) Cardiac mast cells cause atrial fibrillation through PDGF-A-mediated fibrosis in pressure-overloaded mouse hearts. *J Clin Invest*. **120**, 242-253. doi: 10.1172/jci39942

Lim, P.H., Wert, S.L., Tunc-Ozcan, E., Marr, R., Ferreira, A., and Redei, E.E. (2018) Premature hippocampus-dependent memory decline in middle-aged females of a genetic rat model of depression. *Behav Brain Res*. pii: S0166-4328(17)31740-0. doi: 10.1016/j.bbr.2018.02.030

Lin, H.Y., Peng, C.W., and Wu, W.W. (2014) Fibrous hydrogel scaffolds with cells embedded in the fibers as a potential tissue scaffold for skin repair. *J Mater Sci Mater Med*. **25**, 259-269. doi: 10.1007/s10856-013-5065-4

Lindahl, M., Saarma, M., and Lindholm, P. (2017) Unconventional neurotrophic factors CDNF and MANF: structure, physiological functions and therapeutic potential. *Neurobiol Dis*. **97**, 90-102. doi: 10.1016/j.nbd.2016.07.009

Littrell, O.M., Granholm, A.C., Gerhardt, G.A., and Boger, H.A. (2013) Glial cell-line derived neurotrophic factor (GDNF) replacement attenuates motor impairments and nigrostriatal dopamine deficits in 12-month-old mice with a partial deletion of GDNF. *Pharmacol Biochem Behav*. **104**, 10-19. doi: 10.1016/j.pbb.2012.12.022

Liu, X., Liu, Y., Zhao, L., Zeng, Z., Xiao, W., and Chen P. (2017) Macrophage depletion impairs skeletal muscle regeneration: The roles of regulatory factors for muscle regeneration. *Cell Biol Int*. **41**, 228-238. doi: 10.1002/cbin.10705

Loos, M., Mueller, T., Gouwenberg, Y., Wijnands, R., van der Loo, R.J.; Neuro-BSIK Mouse Phenomics Consortium et al. (2014) Neuregulin-3 in the mouse medial prefrontal cortex regulates impulsive action. *Biol Psychiatry*. **76**, 648-655. doi: 10.1016/j.biopsych.2014.02.011

Supplementary Material

Maes, M., Verkerk, R., Bonaccorso, S., Ombelet, W., Bosmans, E., and Scharpe, S. (2002) Depressive and anxiety symptoms in the early puerperium are related to increased degradation of tryptophan into kynurenine, a phenomenon which is related to immune activation. *Life Sci*. **71**, 1837-1848. doi: 10.1016/S0024-3205(02)01853-2

Malabanan, K.P., Sheahan, A.V., and Khachigian, L.M. (2012) Platelet-derived growth factor-BB mediates cell migration through induction of activating transcription factor 4 and tenascin-C. *Am J Pathol*. **180**, 2590-2597. doi: 10.1016/j.ajpath.2012.02.009

Mansukhani, A., Bellosta, P., Sahni, M., and Basilico, C. (2000) Signaling by fibroblast growth factors (FGF) and fibroblast growth factor receptor 2 (FGFR2)-activating mutations blocks mineralization and induces apoptosis in osteoblasts. *J Cell Biol*. **149**, 1297-1308.

Martinez-Ferrer, M., Afshar-Sherif, A.R., Uwamariya, C., de Crombrugghe, B., Davidson, J.M., and Bhowmick, N.A. (2010) Dermal transforming growth factor-beta responsiveness mediates wound contraction and epithelial closure. *Am J Pathol*. **176**, 98-107. doi: 10.2353/ajpath.2010.090283

McCreary, J.K., Truica, L.S., Friesen, B., Yao, Y., Olson, D.M., Kovalchuk, I. et al. (2016) Altered brain morphology and functional connectivity reflect a vulnerable affective state after cumulative multigenerational stress in rats. *Neuroscience*. **330**, 79-89. doi: 10.1016/j.neuroscience.2016.05.046

Mei, L., Huang, Y., Lin, J., Chu, M., Hu, C., Zhou, N., and Wu, L. (2015) Increased cardiac remodeling in cardiac-specific Flt-1 receptor knockout mice with pressure overload. *Cell Tissue Res*. **362**, 389-398. doi: 10.1007/s00441-015-2209-5

Merrill, A.E., Sarukhanov, A., Krejci, P., Idoni, B., Camacho, N., Estrada, K.D. et al. (2012) Bent bone dysplasia-FGFR2 type, a distinct skeletal disorder, has deficient canonical FGF signaling. *Am J Hum Genet*. **90**, 550-557. doi: 10.1016/j.ajhg.2012.02.005

Meyer, M., Muller, A.K., Yang, J., Moik, D., Ponzio, G., Ornitz, D.M. et al. (2012) FGF receptors 1 and 2 are key regulators of keratinocyte migration in vitro and in wounded skin. *J Cell Sci*. **125**, 5690-5701. doi: 10.1242/jcs.108167

Missig, G., Mei, L., Vizzard, M.A., Braas, K.M., Waschek, J.A., Ressler, K.J. et al. (2017) Parabrachial pituitary adenylate cyclase-activating polypeptide activation of amygdala endosomal extracellular signal-regulated kinase signaling regulates the emotional component of pain. *Biol Psychiatry*. **81**, 671-682. doi: 10.1016/j.biopsych.2016.08.025

Mitschelen, M., Yan, H., Farley, J.A., Warrington, J.P., Han, S., and Herenu, C.B. et al. (2011) Long-term deficiency of circulating and hippocampal insulin-like growth factor I induces depressive behavior in adult mice: a potential model of geriatric depression. *Neuroscience*. **185**, 50-60. doi: 10.1016/j.neuroscience.2011.04.032

Miura, M., Sasaki, M., Mizukoshi, K., Shibasaki, M., Izumi, Y., Shimosato, G., and Amaya, F. (2011) Peripheral sensitization caused by insulin-like growth factor 1 contributes to pain hypersensitivity after tissue injury. *Pain*. **152**, 888-895. doi: 10.1016/j.pain.2011.01.004

Moore, S.W., and Zaahl, M. (2012) The Hirschsprung's-multiple endocrine neoplasia connection. *Clinics (Sao Paulo*). **67**, 63-67. doi: 10.6061/clinics/2012(Sup01)12

Myint, A.M., Leonard, B.E., Steinbusch, H.W., and Kim, Y.K. (2005) Th1, Th2, and Th3 cytokine alterations in major depression. *J Affect Disord*. **88**, 167-173. doi: 10.1016/j.jad.2005.07.008

Nicot, A., Otto, T., Brabet, P., and Dicicco-Bloom, E.M. (2004) Altered social behavior in pituitary adenylate cyclase-activating polypeptide type I receptor-deficient mice. *J Neurosci*. **24**, 8786-8795. doi: 10.1523/jneurosci.1910-04.2004

Supplementary Material

Niklasson, M., Bergstrom, T., Zhang, X.Q., Gustafsdottir, S.M., Sjogren, M., Edqvist, P.H. et al. (2010) Enlarged lateral ventricles and aberrant behavior in mice overexpressing PDGF-B in embryonic neural stem cells. *Exp Cell Res*. **316**, 2779-2789. doi: 10.1016/j.yexcr.2010.07.009

O'Tuathaigh, C.M., O'Connor, A.M., O'Sullivan, G.J., Lai, D., Harvey, R., Croke, D.T., and Waddington, J.L. (2008) Disruption to social dyadic interactions but not emotional/anxiety-related behaviour in mice with heterozygous 'knockout' of the schizophrenia risk gene neuregulin-1. *Prog Neuropsychopharmacol Biol Psychiatry*. **32**, 462-466. doi: 10.1016/j.pnpbp.2007.09.018Oppenheim, R.W., Houenou, L.J., Parsadanian, A.S., Prevette, D., Snider, W.D., and Shen, L. (2000) Glial cell line-derived neurotrophic factor and developing mammalian motoneurons: regulation of programmed cell death among motoneuron subtypes. *J Neurosci*. **20**, 5001-5011. doi: 10.1523/jneurosci.20-13-05001.2000

Oglodek, E.A., Just, M.J., Szromek, A.R., and Araszkiewicz, A. (2016) Melatonin and neurotrophins NT-3, BDNF, NGF in patients with varying levels of depression severity. *Pharmacol Rep*. **68**, 945-951. doi: 10.1016/j.pharep.2016.04.003

Pellet-Many, C., Mehta, V., Fields, L., Mahmoud, M., Lowe, V., Evans, I. et al. (2015) Neuropilins 1 and 2 mediate neointimal hyperplasia and re-endothelialization following arterial injury. *Cardiovasc Res*. **108**, 288-298. doi: 10.1093/cvr/cvv229

Peplow, P.V., and Baxter, G.D. (2012) Gene expression and release of growth factors during delayed wound healing: a review of studies in diabetic animals and possible combined laser phototherapy and growth factor treatment to enhance healing. *Photomed Laser Surg*. **30**, 617-636. doi: 10.1089/pho.2012.3312

Peruga, I., Hartwig, S., Merkler, D., Thone, J., Hovemann, B., Juckel, G. et al. (2012) Endogenous ciliary neurotrophic factor modulates anxiety and depressive-like behavior. *Behav Brain Res*. **229**, 325-332. doi: 10.1016/j.bbr.2012.01.020

Powers, J.F., Picard, K.L., and Tischler, A.S. (2009) RET expression and neuron-like differentiation of pheochromocytoma and normal chromaffin cells. *Horm Metab Res*. **41**, 710-714. doi: 10.1055/s-0029-1224136

Pusic, K.M., Pusic, A.D., Kemme, J., and Kraig, R.P. (2014) Spreading depression requires microglia and is decreased by their M2a polarization from environmental enrichment. *Glia*. **62**, 1176-1194. doi: 10.1002/glia.22672

Reif, A., Schmitt, A., Fritzen, S., Chourbaji, S., Bartsch, C., Urani, A. et al. (2004) Differential effect of endothelial nitric oxide synthase (NOS-III) on the regulation of adult neurogenesis and behaviour. *Eur J Neurosci*. **20**, 885-895. doi: 10.1111/j.1460-9568.2004.03559.x

Saito, A., Higuchi, I., Nakagawa, M., Saito, M., Uchida, Y., Inose, M. et al. (2000) An overexpression of fibroblast growth factor (FGF) and FGF receptor 4 in a severe clinical phenotype of facioscapulohumeral muscular dystrophy. *Muscle Nerve*. **23**, 490-497. doi: 10.1002/(sici)1097-4598(200004)23:4<490::aid-mus6>3.0.co;2-k

Santos, M., D'Amico, D., Spadoni, O., Amador-Arjona, A., Stork, O., and Dierssen, M. (2013) Hippocampal hyperexcitability underlies enhanced fear memories in TgNTRK3, a panic disorder mouse model. *J Neurosci*. **33**, 15259-15271. doi: 10.1523/jneurosci.2161-13.2013

Sarin, S., Boivin, F., Li, A., Lim, J., Svajger, B., Rosenblum, N.D., and Bridgewater, D. (2014) β-Catenin overexpression in the metanephric mesenchyme leads to renal dysplasia genesis via cell-autonomous and non-cell-autonomous mechanisms. *Am J Pathol*. **184**, 1395-1410. doi: 10.1016/j.ajpath.2014.01.018

Sautter, N.B., Delaney, K.L., Hausman, F.A., and Trune, D.R. (2012) Tissue remodeling gene expression in a murine model of chronic rhinosinusitis. *Laryngoscope*. **122**, 711-717. doi: 10.1002/lary.22148

Scearce-Levie, K., Roberson, E.D., Gerstein, H., Cholfin, J.A., Mandiyan, V.S., Shah, N.M. et al. (2008) Abnormal social behaviors in mice lacking Fgf17. *Genes Brain Behav*. **7**, 344-354. doi: 10.1111/j.1601-183X.2007.00357.x

Supplementary Material

Score, J., Curtis, C., Waghorn, K., Stalder, M., Jotterand, M., Grand, F.H., and Cross, N.C. (2006) Identification of a novel imatinib responsive KIF5B-PDGFRA fusion gene following screening for PDGFRA overexpression in patients with hypereosinophilia. *Leukemia*. **20**, 827-832. doi: 10.1038/sj.leu.2404154

Sefat, F., Denyer, M.C., and Youseffi, M. (2014) Effects of different transforming growth factor beta (TGF-β) isomers on wound closure of bone cell monolayers. *Cytokine*. **69**, 75-86. doi: 10.1016/j.cyto.2014.05.010

Sherry, S.T., Ward, M.H., Kholodov, M., Baker, J., Phan, L., Smigielski, E.M., et al. (2001). dbSNP: the NCBI database of genetic variation. *Nucleic Acids Res*. **29**, 308–311. doi:10.1093/nar/29.1.308

Shi, Y., Shu, J., Liang, Z., Yuan, S., and Tang, S.J. (2016) EXPRESS: Oligodendrocytes in HIV-associated pain pathogenesis. *Mol Pain*. **12**: 1744806916656845. doi: 10.1177/1744806916656845

Slaets, H., Nelissen, S., Janssens, K., Vidal, P.M., Lemmens, E., Stinissen, P. et al. (2014) Oncostatin M reduces lesion size and promotes functional recovery and neurite outgrowth after spinal cord injury. *Mol Neurobiol*. **50**, 1142-1151. doi: 10.1007/s12035-014-8795-5

Slattery, M.L., Pellatt, D.F., Mullany, L.E., and Wolff, R.K. (2015) Differential gene expression in colon tissue associated with diet, lifestyle, and related oxidative stress. *PLoS One*. **10**: e0134406. doi: 10.1371/journal.pone.0134406

Sleeman, I.J., Boshoff, E.L., and Duty, S. (2012) Fibroblast growth factor-20 protects against dopamine neuron loss in vitro and provides functional protection in the 6-hydroxydopamine-lesioned rat model of Parkinson's disease. *Neuropharmacology*. **63**, 1268-1277. doi: 10.1016/j.neuropharm.2012.07.029

Srivastava, A.S., Shenouda, S., Mishra, R., and Carrier, E. (2006) Transplanted embryonic stem cells successfully survive, proliferate, and migrate to damaged regions of the mouse brain. *Stem Cells*. **24**, 1689-1694. doi: 10.1634/stemcells.2005-0531

Strickland, E.R., Woller, S.A., Hook, M.A., Grau, J.W., and Miranda, R.C. (2014) The association between spinal cord trauma-sensitive miRNAs and pain sensitivity, and their regulation by morphine. *Neurochem Int*. **77**, 40-49. doi: 10.1016/j.neuint.2014.05.005

Sun, L., Xu, L., Chang, H., Henry, F.A., Miller, R.M., Harmon, J.M., and Nielsen, T.B. (1997) Transfection with aFGF cDNA improves wound healing. *J Invest Dermatol*. **108**, 313-318. doi: 10.1111/1523-1747.ep12286471

Sun, Y., Jin, K., Childs, J.T., Xie, L., Mao, X.O., and Greenberg, D.A. (2006) Vascular endothelial growth factor-B (VEGFB) stimulates neurogenesis: evidence from knockout mice and growth factor administration. *Dev Biol*. **289**, 329-335. doi: 10.1016/j.ydbio.2005.10.016Tanaka, M., Hirabayashi, Y., Sekiguchi, T., Inoue, T., Katsuki, M., and Miyajima, A. (2003) Targeted disruption of oncostatin M receptor results in altered hematopoiesis. *Blood*. **102**, 3154-3162. doi: 10.1182/blood-2003-02-0367

Their, M., Hall, M., Heath, J.K., Pennica, D., and Weis, J. (1999) Trophic effects of cardiotrophin-1 and interleukin-11 on rat dorsal root ganglion neurons in vitro. *Brain Res Mol Brain Res*. **64**, 80-84. doi: 10.1016/S0169-328X(98)00329-5

Tian, W., Han, X.G., Liu, Y.J., Tang, G.Q., Liu, B. et al. (2013) Intrathecal epigallocatechin gallate treatment improves functional recovery after spinal cord injury by upregulating the expression of BDNF and GDNF. *Neurochem Res*. **38**, 772-779. doi: 10.1007/s11064-013-0976-5

Toriseva, M., Laato, M., Carpen, O., Ruohonen, S.T., Savontaus, E., Inada, M. et al. (2012) MMP-13 regulates growth of wound granulation tissue and modulates gene expression signatures involved in inflammation, proteolysis, and cell viability. *PLoS One*. **7**: e42596. doi: 10.1371/journal.pone.0042596

Supplementary Material

Trystula, M., Żychowska, M., Wilk-Franczuk, M., Kropotov, J.D., and Pachalska, M. (2017) CASE-REPORT Dysregulation of gene expression in a patient with depressive disorder after transient ischemic attack confirmed by a neurophysiological neuromarker. *Genet Mol Res*. **16**: gmr16019532. doi: 10.4238/gmr16019532

Tseng, P.T., Chen, Y.W., Tu, K.Y., Wang, H.Y., Chung, W., Wu, C.K. et al. (2016) State-dependent increase in the levels of neurotrophin-3 and neurotrophin-4/5 in patients with bipolar disorder: A meta-analysis. *J Psychiatr Res*. **79**, 86-92. doi: 10.1016/j.jpsychires.2016.05.009

Uutela, M., Wirzenius, M., Paavonen, K., Rajantie, I., He, Y., Karpanen, T. et al. (2004) GF-D induces macrophage recruitment, increased interstitial pressure, and blood vessel maturation during angiogenesis. *Blood*. **104**, 3198-1204. doi: 10.1182/blood-2004-04-1485

Wagatsuma, A. (2007) Endogenous expression of angiogenesis-related factors in response to muscle injury. *Mol Cell Biochem*. **298**, 151-159. doi: 10.1007/s11010-006-9361-x

Wang, S., and Albers, K.M. (2009) Behavioral and cellular level changes in the aging somatosensory system. *Ann N Y Acad Sci*. **1170**, 745-749. doi: 10.1111/j.1749-6632.2009.04011.x

Wang, G., Dai, D., Chen, X., Yuan, L., Zhang, A., Lu, Y., and Zhang, P. (2013) Upregulation of neuregulin-1 reverses signs of neuropathic pain in rats. *Int J Clin Exp Pathol*. **7**, 5916-5921.

Wang, T., Jing, X., DeBerry, J.J., Schwartz, E.S., Molliver, D.C., Albers, K.M., and Davis, B.M. (2013) Neurturin overexpression in skin enhances expression of TRPM8 in cutaneous sensory neurons and leads to behavioral sensitivity to cool and menthol. *J Neurosci*. **33**, 2060-2070. doi: 10.1523/jneurosci.4012-12.2013

Wang, H.J., Song, G., Liang, J., Gao, Y.Y., and Wang, C.J. (2017) Involvement of integrin β1/FAK signaling in the analgesic effects induced by glial cell line-derived neurotrophic factor in neuropathic pain. *Brain Res Bull*. **135**, 149-156. doi: 10.1016/j.brainresbull.2017.10.008

Wang, C., Wang, Q., Gao, W., Zhang, Z., Lou, Y., Jin, H. et al. (2018) Highly efficient local delivery of endothelial progenitor cells significantly potentiates angiogenesis and full-thickness wound healing. *Acta Biomater*. **69**, 156-169. doi: 10.1016/j.actbio.2018.01.019

Wehrhan, F., Grabenbauer, G.G., Rodel, F., Amann, K., and Schultze-Mosgau, S. (2004) Exogenous modulation of TGF-beta(1) influences TGF-betaR-III-associated vascularization during wound healing in irradiated tissue. *Strahlenther Onkol*. **180**, 526-533. doi: 10.1007/s00066-004-1212-7

Wu, C.H., Ho, W.Y., Lee, Y.C., Lin, C.L., and Hsieh, Y.L. (2016) EXPRESS: NGF-trkA signaling modulates the analgesic effects of prostatic acid phosphatase in resiniferatoxin-induced neuropathy. *Mol Pain*. 12: 1744806916656846. doi: 10.1177/1744806916656846

Xiyang, Y.B., Liu, S., Liu, J., Hao, C.G., Wang, Z.J., Ni, W. et al. (2009) Roles of platelet-derived growth factor-B expression in the ventral horn and motor cortex in the spinal cord-hemisected rhesus monkey. *J Neurotrauma*. **26**, 275-287. doi: 10.1089/neu.2007.0374

Xu, M.M., Zhang, D.M., Shi, R.X., Zhang, X.H., Wang, Y., Yu, M. et al. (2016) Effect of electroacupuncture intervention on behavior changes and levels of hippocampal transforming growth factor beta 3 and basic fibroblast growth factor proteins in depression rats. *Zhen Ci Yan Jiu*. **41**, 138-143.

Xu, Y.H., Yu, M., Wei, H., Yao, S., Chen, S.Y., Zhu, X.L., and Li, Y.F. (2017b) Fibroblast growth factor 22 is a novel modulator of depression through interleukin-1β. *CNS Neurosci Ther*. **23**, 907-916. doi: 10.1111/cns.12760

Yamano, S., Kuo, W.P., and Sukotjo C. (2013) Downregulated gene expression of TGF-βs in diabetic oral wound healing. *J Craniomaxillofac Surg*. **41**, e42-e48. doi: 10.1016/j.jcms.2012.08.001

Supplementary Material

Yan, J., Mitra, A., Hu, J., Cutrera, J.J., Xia, X., Doetschman, T. et al. (2016) Interleukin-30 (IL27p28) alleviates experimental sepsis by modulating cytokine profile in NKT cells. *J Hepatol*. **64**, 1128-1136. doi: 10.1016/j.jhep.2015.12.020

Yan, L., Shamir, A., Skirzewski, M., Leiva-Salcedo, E., Kwon, O.B., Karavanova, I. et al. (2018) Neuregulin-2 ablation results in dopamine dysregulation and severe behavioral phenotypes relevant to psychiatric disorders. *Mol Psychiatry*. **23**, 1233-1243. doi: 10.1038/mp.2017.22

Yang, J., Lindahl, M., Lindholm, P., Virtanen, H., Coffey, E., Runeberg-Roos, P., and Saarma, M. (2004) PSPN/GFRalpha4 has a significantly weaker capacity than GDNF/GFRalpha1 to recruit RET to rafts, but promotes neuronal survival and neurite outgrowth. *FEBS Lett*. **569,** 267-271. doi: 10.1016/j.febslet.2004.06.007

Yang, P., Schmit, B.M., Fu, C., DeSart, K., Oh, S.P., Berceli, S.A., and Jiang, Z. (2016) Smooth muscle cell-specific Tgfbr1 deficiency promotes aortic aneurysm formation by stimulating multiple signaling events. *Sci Rep*. **6**: 35444. doi: 10.1038/srep35444

Zaman, V., Boger, H.A., Granholm, A.C., Rohrer, B., Moore, A., Buhusi, M. et al. (2008) The nigrostriatal dopamine system of aging GFRalpha-1 heterozygous mice: neurochemistry, morphology and behavior. *Eur J Neurosci*. **28**, 1557-1568. doi: 10.1111/j.1460-9568.2008.06456.x

Zhan, J., Xiu, Y., Gu, J., Fang, Z., and Hu, X.L. (1999) Expression of RET proto-oncogene and GDNF deficit in Hirschsprung's disease. *J Pediatr Surg*. **34**, 1606-1609. doi: 10.1016/s0022-3468(99)90626-7

Zhang, M., Ueki, S., Kimura, S., Yoshida, O., Castellaneta, A., Ozaki, K.S. et al. (2013) Roles of dendritic cells in murine hepatic warm and liver transplantation-induced cold ischemia/reperfusion injury. *Hepatology*. **57**, 1585-1596. doi: 10.1002/hep.26129

Zhang, J., Chen, A., Wu, Y., and Zhao, Q. (2015) Placental growth factor promotes cardiac muscle repair via enhanced neovascularization. *Cell Physiol Biochem*. **36**, 947-955. doi: 10.1159/000430269

Zhao, P., Caretti, G., Mitchell, S., McKeehan, W.L., Boskey, A.L., Pachman, L.M. et al. (2006) Fgfr4 is required for effective muscle regeneration in vivo. Delineation of a MyoD-Tead2-Fgfr4 transcriptional pathway. *J Biol Chem*. **281**, 429-438. doi: 10.1074/jbc.M507440200

Zhou, K., Ma, Y., and Brogan, M.S. (2015) Chronic and non-healing wounds: The story of vascular endothelial growth factor. *Med Hypotheses*. **85**, 399-404. doi: 10.1016/j.mehy.2015.06.017

Zhu, L., Wei, T., Gao, J., Chang, X., He, H., Miao, M., and Yan, T. (2015) Salidroside attenuates lipopolysaccharide (LPS) induced serum cytokines and depressive-like behavior in mice. *Neurosci Lett*. **606**, 1-6. doi: 10.1016/j.neulet.2015.08.025

Zuo, P., Fu, Z., Tao, T., Ye, F., Chen, L., Wang, X. et al. (2013) The expression of glia maturation factors and the effect of glia maturation factor-γ on angiogenic sprouting in zebrafish. *Exp Cell Res*. **319**, 707-717. doi: 10.1016/j.yexcr.2013.01.004
