# Supplementary material for: Natural Selection Equally Supports the Human Tendencies in Subordination and Domination: A Genome-Wide Study With in silico Confirmation and in vivo Validation in Mice
Source: Front Genet. 2019 Feb 20;10:73. doi: 10.3389/fgene.2019.00073 (PMC6404730; doi:10.3389/fgene.2019.00073)
Supplement: Supplementary file 4 [file Data_Sheet_4.PDF]

Supplementary Material

Natural selection equally supports the human tendencies in subordination and domination: a genome-wide study with *in silico* confirmation and *in vivo* validation in mice

Irina Chadaeva, Petr Ponomarenko, Dmitry Rasskazov, Ekaterina Sharypova, Elena Kashina, Maxim Kleshchev, Mikhail Ponomarenko*, Vladimir Naumenko, Ludmila Savinkova, Nikolay Kolchanov, Ludmila Osadchuk, Alexandr Osadchuk

*** Correspondence:** Mikhail Ponomarenko (pon@bionet.nsc.ru)

Supplementary Method

An estimate of binding affinity of TATA-binding protein for 70 bp regions in front of transcription start sites of human genes

The input data are the 70 bp DNA sequence $\{s_{-70}\ldots s_i\ldots s_{-1}\}$, where s_0 is he transcription start site (TSS) and $s_i \in \{a, c, g, t\}$). We use the linear approximation for the three-step molecular mechanism of the TATA-binding protein (TBP) binding to the 70 bp region of the human gene promoters (Ponomarenko et al., 2008; Delgadillo et al., 2009). This mechanism can be described as follows: (i) TBP slides along DNA ↔ (ii) TBP stops at a probable TBP-binding site ↔ TBP–DNA complex is fixed by DNA bending at a right angle, i.e.,

$$-\ln(K_D) = 10.9 - 0.2\ \{\ln(K_{SLIDE}) + \ln(K_{STOP}) + \ln(K_{BEND})\}, \quad (1)$$

where 10.9 (ln units) is nonspecific TBP–DNA affinity (10^{-5} M); $-\ln(K_{STOP})$ is calculated using Bucher's matrix, $W_{i,s(i)}$ is weight of nucleotide *s(i)* at the *i*th position of the TBP-binding site (Bucher, 1990):

$$\boldsymbol{ln(K_{STOP})} = \max_{(+),(-)\ \boldsymbol{DNA\ chains}} \left\{\sum\nolimits_{\boldsymbol{j=-1}}^{\boldsymbol{13}} \boldsymbol{w_{j;s_{i+j}}}\right\}; \quad (2)$$

$-\ln(K_{SLIDE})$ is calculated using the abundance [TA] of dinucleotide TA and μ-value of the minor groove width of the DNA helix (Karas et al., 1996), namely:

$$-\ln(K_{SLIDE}) = MEAN_{15bp}\ \{0.8[TA] + 3.4\mu + 35.1\}; \quad (3)$$

$-\ln(K_{BEND})$ is calculated using the means for both DNA strands of the TBP-binding site at the maximal score value of Eq. (2), as follows:

$$-\ln(K_{BEND}) = MEAN_{TATA\text{-}box}\ \{0.9[TA, AA, TG, AG] + 2.5[TA, TC, TG] +14.4\}. \quad (4)$$

Using all the possible substitutions, $s_j \rightarrow \xi$, at each position j within the 26-bp DNA window scanning the 70 bp DNA under study, we estimated the standard deviation of the $-\ln[K_D]$ estimates (Eq. 1) as

$$\delta=[(\Sigma_{1\le i\le 26}\Sigma_{\xi\in\{a,c,g,t\}}[\ln(K_D(\{s_{i-13}\ldots\xi\ldots s_{i+12}\})/K_D(\{s_{i-13}\ldots{}_1 s_{i+j}\ldots s_{i+12}\})^2])/(3*26)]^{1/2} \quad (5)$$

Applying Eqs. (1–5) to both minor (min) and ancestral (wt) alleles of the DNA being studied, we calculated ($-\ln(K_D^{(min)}) \pm \delta_{(min)}$) and ($-\ln(K_D^{(wt)}) \pm \delta_{(wt)}$), respectively, and, after that, computed Fisher's Z-score:

$$Z = \mathrm{abs}[\ln(K_D^{(min)}/K_D^{(wt)})]/[\delta^2_{(min)}+\delta^2_{(wt)}]^{1/2}. \qquad (6)$$

Next, package R (Waardenberg et al., 2015) transforms this Z-score value into a p value, i.e., the probability of the hypothesis "H_0: $K_D^{(mut)} \neq K_D^{(wt)}$". At this statistically significant level $p > 0.95$, we made the final decision:

IF {*INEQUALITY* "$-\ln(K_D^{(min)}) > -\ln(K_D^{(wt)})$" is statistically significant},

THEN {*DECISION* is "the minor allele of the given gene is overexpressed relative to the ancestral one"};

ELSE [**IF** {*INEQUALITY* "$-\ln(K_D^{(min)}) < -\ln(K_D^{(wt)})$" is statistically significant},

THEN {*DECISION* is "the minor allele of this gene is underexpressed relative to the ancestral one"},]

OTHERWISE {*DECISION* is "alteration of the expression of this gene is insignificant"}.

This DECISION is the third line of textbox "Result" of our Web service SNP_TATA_Comparator [1] .

References

Bucher, P. (1990). Weight matrix descriptions of four eukaryotic RNA polymerase II promoter elements derived from 502 unrelated promoter sequences. *J Mol Biol*. **212**, 563-578. doi:10.1016/0022-2836(90)90223-9

Delgadillo, R.F., Whittington, J.E., Parkhurst, L.K., and Parkhurst, L.J. (2009). The TATA-binding protein core domain in solution variably bends TATA sequences via a three-step binding mechanism. *Biochemistry*. **48**, 1801-1809. doi:10.1021/bi8018724

Karas, H., Knuppel, R., Schulz, W., Sklenar, H., and Wingender, E. (1996). Combining structural analysis of DNA with search routines for the detection of transcription regulatory elements. *Comput Appl Biosci*. **12**, 441-446.

Ponomarenko, P.M., Savinkova, L.K., Drachkova, I.A., Lysova, M.V., Arshinova, T.V., Ponomarenko, M.P., and Kolchanov, N.A. (2008). A step-by-step model of TBP/TATA box binding allows predicting human hereditary diseases by single nucleotide polymorphism. *Dokl Biochem Biophys*. **419**, 88-92. doi:10.1134/S1607672908020117

Waardenberg, A.J., Basset, S.D., Bouveret, R., and Harvey, R.P. (2015). CompGO: an R package for comparing and visualizing Gene Ontology enrichment differences between DNA binding experiments. *BMC Bioinformatics*. **16**:275. doi:10.1186/s12859-015-0701-2.

[1]http://beehive.bionet.nsc.ru/cgi-bin/mgs/tatascan/start.pl
