# Supplementary material for: Natural Selection Equally Supports the Human Tendencies in Subordination and Domination: A Genome-Wide Study With in silico Confirmation and in vivo Validation in Mice
Source: Front Genet. 2019 Feb 20;10:73. doi: 10.3389/fgene.2019.00073 (PMC6404730; doi:10.3389/fgene.2019.00073)
Supplement: Supplementary file 5 [file Data_Sheet_5.PDF]

Supplementary Material

Natural selection equally supports the human tendencies in subordination and domination: a genome-wide study with *in silico* confirmation and *in vivo* validation in mice

Irina Chadaeva, Petr Ponomarenko, Dmitry Rasskazov, Ekaterina Sharypova, Elena Kashina, Maxim Kleshchev, Mikhail Ponomarenko*, Vladimir Naumenko, Ludmila Savinkova, Nikolay Kolchanov, Ludmila Osadchuk, Alexandr Osadchuk

*** Correspondence:** Mikhail Ponomarenko (pon@bionet.nsc.ru)

Supplementary Web service

How to use SNP_TATA_Comparator (the example of SNP rs886056538 of the human *PDYN* gene promoter predicted by this work for the human tendency in dominance)

We applied our Web service SNP_TATA_Comparator (Ponomarenko et al., 2015) to retrieve the 70 bp promoter sequence of each gene studied from public database Ensembl (Zerbino et al., 2015) using the BioPerl library (Stajich et al., 2002) and the Web service UCSC Genome Browser (Haeussler et al., 2015) as shown in Figures S1(D) and S1(A), respectively. In addition, we used two public databases dbSNP (Sherry et al., 2001) and ClinVar (Landrum et al., 2014) to find the descriptions of SNPs located within these DNA sequences according to the reference human genome and its variome (Colonna et al., 2014) as presented in Figures S1(B) and S1(C), respectively.

Next, by means of the textbox "Editable sequence" shown in this figure, we manually modified the ancestral allele of the DNA sequence in question into its minor allele in the cases of each SNP under study, which was retrieved from database dbSNP (Sherry et al., 2001).

Then, by clicking "Calculate," we obtained a statistical estimate of the significance of the difference between the affinity of TBP for these ancestral and minor variants of the promoter in question that appeared in the textbox "Result" as soon as our Web service calculated it, as described in the Supplementary method (Supplementary file 4). As one can see in this textbox, our results contain two pairs of ($-\ln(K_D^{(wt)}) \pm \delta_{(wt)}$) and ($-\ln(K_D^{(min)}) \pm \delta_{(min)}$) values of the TBP affinity for the ancestral and minor alleles of the promoter being studied, which both were accompanied by standard errors, respectively.

After that, we computed Fisher's Z-score, namely: $Z = abs[\ln(K_D^{(min)}/K_D^{(wt)})]/[\delta^2_{(min)}+\delta^2_{(wt)}]^{1/2}$.

Finally, using package R (Waardenberg et al., 2015), we transformed this Z-score value into a *p*-value, i.e., the probability of the hypothesis "H_0: $K_D^{(mut)} \neq K_D^{(wt)}$". At this statistically significant level $p > 0.95$, we made the final decision on "whether difference between these alleles is significant or insignificant" and the likelihood *p* value of observing this or greater difference between them for random reasons. To this end, we employed the standard statistical package R (Waardenberg et al., 2015) which returns *p*-values as output in response to a Z-score as its input, as illustrated in Figure S1(D). Using this *p*-value, we discarded all the SNPs whose effects were estimated as insignificant as shown in Figure S1(D), line "DECISION." Thus; we predicted the remaining SNPs to be the candidate SNP markers that can significantly increase or decrease the binding affinity of TBP for the analyzed promoters that can cause excess or deficiency of the protein products of the appropriate genes, respectively, as demonstrated experimentally (Mogno et al., 2010).

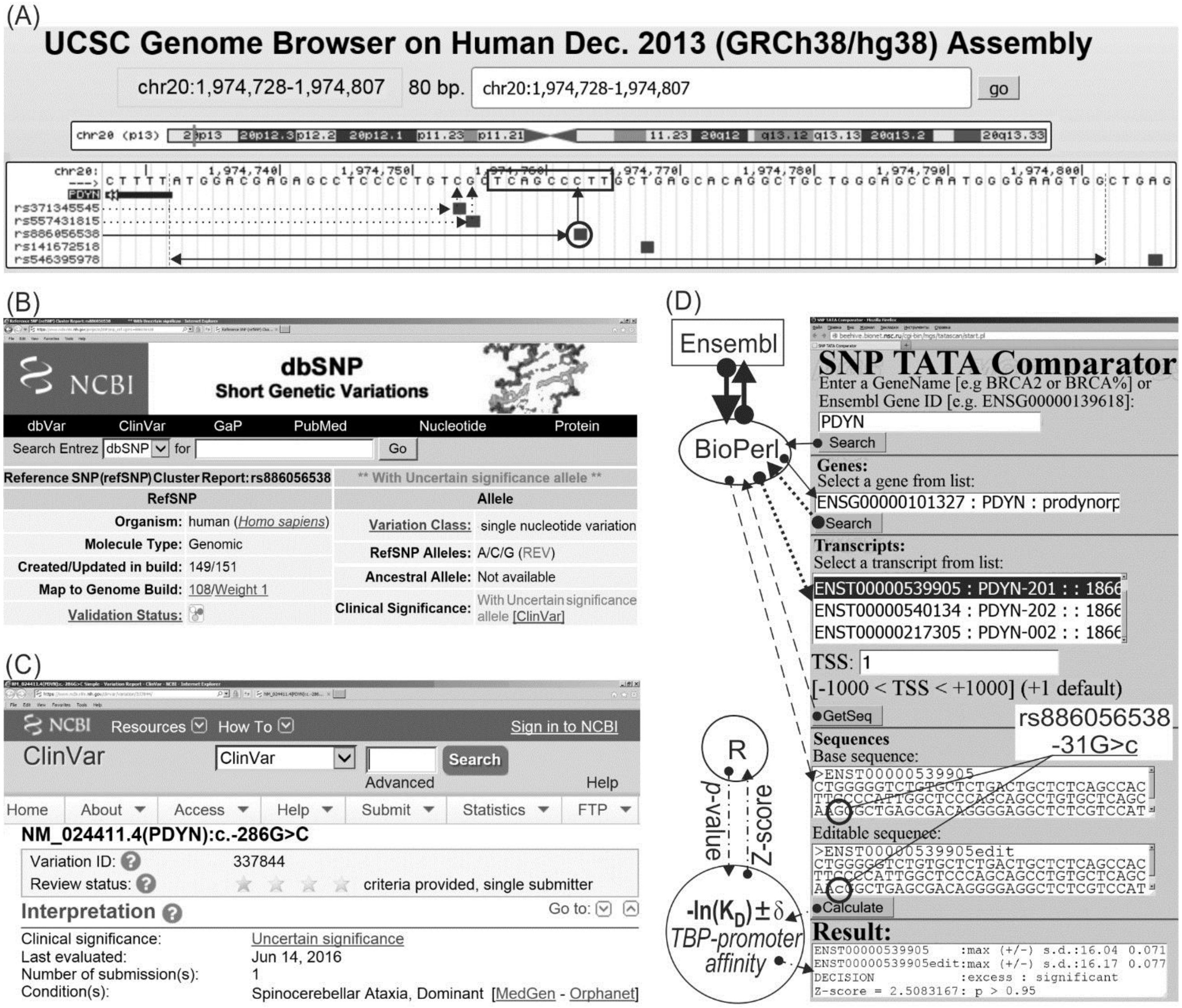

Figure S1: The candidate SNP marker rs886056538 predicted by this work for the human tendency in dominance. (A) Unannotated SNPs (analyzed in this study) in the 70 bp region [where all known TBP-binding sites (boxed) are located; double-headed arrow, ↔] of the human *PDYN* gene promoter using the UCSC Genome Browser (Haeussler et al., 2015). Solid arrow: SNP rs886056538 under study. Dotted arrows: two more candidate SNP markers of the human tendencies in dominance and subordination that were predicted in this work near the analyzed SNP rs886056538. (B) The description of rs886056538 within database dbSNP (Sherry et al., 2001). (C) The description of rs886056538 in database ClinVar (Landrum et al., 2014). (D) The results from our Web service (Ponomarenko et al., 2015) for SNP rs886056538 under study. Solid, dotted, and dashed arrows indicate queries for the gene list, a list of transcripts of a certain gene, and DNA sequence of the promoter corresponding to the specified transcript by means of the BioPerl library (Stajich et al., 2002) of database Ensembl (Zerbino et al., 2015) of the reference human genome (Colonna et al., 2014), respectively. Dash-and-dot arrows: estimates of significance of alteration of the PDYN level in patients with the minor allele (min) relative to the norm (ancestral allele, wt) expressed as a Z-score using package R (Waardenberg et al., 2015). Circles indicate the ancestral (wt) and minor (min) alleles of the SNP marker labeled by its dbSNP ID (Sherry et al., 2001).

References

Colonna, V., Ayub, Q., Chen, Y., Pagani, L., Luisi, P., Pybus, M., et al. (2014). Human genomic regions with exceptionally high levels of population differentiation identified from 911 whole-genome sequences. *Genome Biol*. **15**: R88. doi:10.1186/gb-2014-15-6-r88

Haeussler, M., Raney, B.J., Hinrichs, A.S., Clawson, H., Zweig, A.S., Karolchik, D., et al. (2015). Navigating protected genomics data with UCSC Genome Browser in a Box. *Bioinformatics*. **31**, 764–766. doi:10.1093/bioinformatics/btu712

Landrum, M.J., Lee, J.M., Riley, G.R., Jang, W., Rubinstein, W.S., Church, D.M., et al. (2014). ClinVar: public archive of relationships among sequence variation and human phenotype. *Nucleic Acids Res*. 42, D980-D985. doi:10.1093/nar/gkt1113

Mogno, I., Vallania, F., Mitra, R.D., and Cohen, B.A. (2010) TATA is a modular component of synthetic promoters. *Genome Res*. **20**, 1391–1397. doi:10.1101/gr.106732.110

Ponomarenko, M., Rasskazov, D., Arkova, O., Ponomarenko, P., Suslov, V., Savinkova, L., et al. (2015). How to use SNP_TATA_Comparator to find a significant change in gene expression caused by the regulatory SNP of this gene's promoter via a change in affinity of the TATA-binding protein for this promoter. *Biomed Res Int.* **2015**: 359835. doi:10.1155/2015/359835

Sherry, S.T., Ward, M.H., Kholodov, M., Baker, J., Phan, L., Smigielski, E.M., et al. (2001). dbSNP: the NCBI database of genetic variation. *Nucleic Acids Res*. **29**, 308–311. doi:10.1093/nar/29.1.308

Stajich, J.E., Block, D., Boulez, K., Brenner, S.E., Chervitz, S.A., Dagdigian, C., et al. (2002). The Bioperl toolkit: Perl modules for the life sciences. *Genome Res*. **12**, 1611-1618. doi: 10.1101/gr.361602

Waardenberg, A.J., Basset, S.D., Bouveret, R., and Harvey, R.P. (2015). CompGO: an R package for comparing and visualizing Gene Ontology enrichment differences between DNA binding experiments. *BMC Bioinformatics*. **16**:275. doi:10.1186/s12859-015-0701-2.

Zerbino, D.R., Wilder, S.P., Johnson, N., Juettemann, T., and Flicek, P.R. (2015) The Ensembl regulatory build. *Genome Biol*. **16**: 56. doi 10.1186/s13059-015-0621-5
