# Supplementary material for: Natural Selection Equally Supports the Human Tendencies in Subordination and Domination: A Genome-Wide Study With in silico Confirmation and in vivo Validation in Mice
Source: Front Genet. 2019 Feb 20;10:73. doi: 10.3389/fgene.2019.00073 (PMC6404730; doi:10.3389/fgene.2019.00073)
Supplement: Supplementary file 6 [file Data_Sheet_6.PDF]

Supplementary Material

Natural selection equally supports the human tendencies in subordination and domination: a genome-wide study with *in silico* confirmation and *in vivo* validation in mice

Irina Chadaeva, Petr Ponomarenko, Dmitry Rasskazov, Ekaterina Sharypova, Elena Kashina, Maxim Kleshchev, Mikhail Ponomarenko*, Vladimir Naumenko, Ludmila Savinkova, Nikolay Kolchanov, Ludmila Osadchuk, Alexandr Osadchuk

*** Correspondence:** Mikhail Ponomarenko (pon@bionet.nsc.ru)

Supplementary Experiment

Identification of inheritance of the mouse tendencies in dominance and subordination

This study was carried out in accordance with the recommendations of Directive 2010/63/EU of the European Parliament and of the Council of September 22, 2010, on the protection of animals used for scientific purposes. Manipulations of animals and experimental procedures were performed in compliance with the international rules according to the "Guidelines for the care and use of mammals in neuroscience and behavioral research." The research protocol was approved by the Interinstitutional Commission on Bioethics at the Institute of Cytology and Genetics of the Siberian Branch of the Russian Academy of Sciences, 10 Lavrentyev Avenue, Novosibirsk, 630090, Russia.

The analysis of the inheritance of agonistic behavior indicators and social dominance levels was conducted on 230 adult male mice that are the diallelic crosses of a set of five maternal inbred mouse strains (i.e., PT, DD, YT, A/He, and C57BL/6J) with two inbred paternal strains (BALB/cLac and CBA/Lac), which were analyzed in pairs. These paternal strains of mice have opposite tendencies (in dominance and subordination, respectively), as identified experimentally previously (Bragin et al., 2006; Osadchuk et al., 2009; Kleshchev et al., 2013). One can see all the 230 diallelic crosses in Table 1, where five rows and two columns describe F1 males. In each row of this table, there are offspring of mothers of the same inbred strain. Thus, the maternal nongenetic (pre- and postnatal) and cytoplasmic effects are the same for males of the same row in this table. To exclude nongenetic paternal postnatal effects on the offspring, pregnant female mice were isolated from male mice. Thus, by making up groups of males with minimum society (two males), one from each column of the same row, it is possible to estimate almost purely the influence of the paternal genotype on the level of social dominance.

All the mice (n = 230) were kept under standard conditions of a conventional animal facility of the ICG SB RAS, under artificial light with a photoperiod of 12:12 h; food and water were supplied *ad libitum*. Experimental F1 hybrid males at the age of 1 month were isolated from the mother into standard plastic cages 36 × 20 × 15 cm, where they were kept in same-sex groups of 4–8 males until they reached 3 months of age (90 days). To eliminate the effect of composition of the group and the influence of previous social contacts, 4–5 days before the behavioral experiment, the males were seated in single cells of the same size. The test males were placed in pairs in an experimental cage (28 × 14 × 10 cm), separated in the middle by a nontransparent plastic partition, with individual food and water in each of the two compartments. The animals

were kept for another 2 days to get used to the new conditions and develop the territory. On the third day, the partition was removed, and the experiment started, which lasted 5 days.

A total of 115 experimental pairs (230 F1 hybrids) were set up and distributed into five groups corresponding to the maternal inbred strains (see Table 1). For each pair of hybrids, 14 20-minute observations of agonistic behavior (agonistic tests) were carried out. During the first 4 days, tests were performed three times a day, and on the fifth day, only two times. In each test, agonistic behavior was recorded on a video camera. On the video of each test, the behavior of both males was documented via special protocols of the program The Observer XT 7.0 (version: 7.0, Noldus Information Technology, license # OB070-03670). We registered the following behavioral patterns:

1. An attack: an aggressive action of one of the males, aimed at a partner. For this type of behavior, the number of attacks and their total duration (in seconds) for a 20-minute test were determined.

2. Vertical (protective) submission pose (VSP): rearing on the hind feet with the ventral part of the body to the attacking male. We determined the number of such poses during a 20-minute test.

This way, we identified the social rank for each male within the appropriate pair according to asymmetry in agonistic behavior, in particular, by means of attacks and submissive poses, as shown in Figure 4. The results are listed in Table 3.

References

Bragin, A.V., Osadchuk, L.V., and Osadchuk, A.V. (2006) The experimental model of establishment and maintenance of social hierarchy in laboratory mice. *Zh Vyssh Nerv Deiat Im I P Pavlova*. **56**, 412-419.

Kleshchev, M.A., Gutorova, N.V., and Osadchuk L.V. (2013) Genetic features of developmental patterns of agonistic behavior in laboratory male mice under social hierarchy conditions. *Ecol Genet*. **XI**, 64-72.

Osadchuk, L.V., Bragin, A.V., and Osadchuk, A.V. (2009) Interstrain differences in social and time patterns of agonistic behavior in male laboratory mice. *Zh Vyssh Nerv Deiat Im I P Pavlova*. **59**, 473-481.
